# Genome-Wide Identification and Analysis of the Heat-Shock Protein Gene Superfamily in *Bemisia tabaci* and Expression Pattern Analysis under Heat Shock

**DOI:** 10.3390/insects13070570

**Published:** 2022-06-23

**Authors:** Hao-Yuan Zheng, Peng-Hao Qin, Kun Yang, Tong-Xian Liu, You-Jun Zhang, Dong Chu

**Affiliations:** 1 Shandong Engineering Research Center for Environment-Friendly Agricultural Pest Management, College of Plant Health and Medicine, Qingdao Agricultural University, Qingdao 266109, China; zhy920676850@163.com (H.-Y.Z.); penghao0401@163.com (P.-H.Q.); yangkun@qau.edu.cn (K.Y.); txliu@nwafu.edu.cn (T.-X.L.); 2 Department of Plant Protection, Institute of Vegetables and Flowers, Chinese Academy of Agricultural Sciences, Beijing 100081, China; zhangyoujun@caas.cn

**Keywords:** *Bemisia tabaci* MED, expression pattern, heat shock proteins, thermal stress

## Abstract

**Simple Summary:**

*Bemisia tabaci* MED is an invasive pest that had caused considerable economic damage in the past decades. Its successful colonization is closely related to heat-shock proteins (HSPs), which are related to heat resistance. In this study, 33 *BtaHsp*s were identified based on the sequenced genome of *B. tabaci* MED belonging to six HSP families, among which 22 *BtaHsp*s were newly identified. Analysis of the secondary structure and evolutionary relationship showed that they were all closely related. In addition, *BtaHsp90A3* of the HSP90 family was screened by analyzing the expression level changes of these genes under 42 °C heat shock and RNAi was performed on the *BtaHsp90A3*. The results showed that the silencing of *BtaHsp90A3* is closely related to the heat resistance of *B. tabaci* MED. Taken together, this study conducted an in-depth identification of *BtaHsp*s that clarifies their evolutionary relationships and their response to thermal stress in *B. tabaci* MED.

**Abstract:**

The thermal tolerance of *Bemisia tabaci* MED, an invasive whitefly species with worldwide distribution, plays an important role in its ecological adaptation during the invasion process. Heat-shock proteins (HSPs) are closely related to heat resistance. In this study, 33 *Hsp*s (*BtaHsp*s) were identified based on sequenced genome of *B. tabaci* MED belonging to six HSP families, among which 22 *Hsp*s were newly identified. The secondary structures of a further 22 *BtaHsp*s were also predicted. The results of RT-qPCR showed that heat shock could affect the expression of 14 of the 22 *Hsp*s newly identified in this study. Among them, the expression level of six *Hsp*s increased under 42 °C treatment. As the unstudied gene, *BtaHsp90A3* had the highest increase rate. Therefore, *BtaHsp90A3* was chosen for the RNAi test, and silencing *BtaHsp90A3* by RNAi decreased the survival rate of adult *B. tabaci* at 42 °C. The results indicated that only a few *Hsp*s were involved in the thermal tolerance of host whitefly although many *Hsp*s would response under heat stress. This study conducted a more in-depth and comprehensive identification that demonstrates the evolutionary relationship of *BtaHsp*s and illustrates the response of *BtaHsp*s under the influence of thermal stress in *B. tabaci* MED.

## 1. Introduction

In many insects, heat-shock proteins (HSPs) can overexpress in response to a variety of environmental stresses such as heat [1], cold [2], dehydration [3], UV exposure [4], osmolarity [5], and organic pollutants [6]. Among these abiotic stressors, thermal stress is perhaps the most important factor that commonly activates the increased expression of HSPs in insects [7,8,9]. HSPs exist widely in insects and may play an important role in their heat adaptation, heat tolerance, and heat protection [10,11]. For instance, HSPs play important roles as molecular chaperones in promoting correct protein folding and preventing denatured protein aggregation [12]. Based on molecular weight and homology, HSPs constitute a supergene family that was divided into six families (small HSPs, HSP40, HSP60, HSP70, HSP90, and HSP100) [13,14].

The sweet potato whitefly, *Bemisia tabaci* (Gennadius) species complex, transmits more than 320 plant viruses [15]. One member of this complex species, *B. tabaci* MED has invaded many countries, including China, and has caused considerable economic damage to many important crops [16]. It has been reported that temperature tolerance may have been an important factor in the successful colonization of *B. tabaci* MED in many locations [17,18,19]. The HSP90, HSP70, and sHSP genes were considered to be related to the high temperature tolerance in invasive *B. tabaci* MED [20]. For example, *B. tabaci* MED is more tolerant to short temperature stress than the indigenous *B. tabaci* ZHJ1. Meanwhile, the *Hsp* expression of *B. tabaci* MED was higher than in the indigenous species under maximal temperature, and the onset and maximal temperature induction of *Hsp* expression was generally 2–4 °C higher than in the indigenous species ZHJ1 [21], which is helpful in understanding the relationship between *Hsp*s and the distribution changes of *B. tabaci* MED under global climate warming. In addition, *Hsp* expression of viruliferous *B. tabaci* was more easily upregulated by temperature stress [22]. Previous studies had identified and analyzed 26 *Hsp*s based on the genome of *B. tabaci* MEAM1 [23], and also identified two *Hsp70*s and three *sHsp*s in *B. tabaci* MED [19,24]. However, the information about evolutionary relationships, gene structures, and gene functions of the HSP superfamily in *B. tabaci* MED still remains poorly understood.

To reveal the evolutionary characteristics of *Hsp*s in *B. tabaci* MED and the responses of *Hsp*s to temperature stresses, 22 *Hsp*s were identified from *B. tabaci*, including 11 HSP70 genes, four HSP90 genes, one HSP60 gene, one HSP100 gene, four HSP40 genes, and one sHSP gene. By constructing a phylogenetic tree with all these *B. tabaci* MED *Hsp*s and *Hsp*s from outgroup insects, the phylogenetic relationships of *Hsp*s were explored. The expression pattern of *BtaHsp*s was examined by RT-qPCR by determining the expression level of *BtaHsp*s under treatment at 42 °C. In addition, the functions of HSPs were further clarified by silencing the *Hsp*s whose expression increased under heat treatment. The present results could improve understanding of the mechanisms of thermotolerance in *B. tabaci* at the molecular level.

## 2. Materials and Methods

### 2.1. Insect Collection and Rearing Conditions

The *B. tabaci* population in this study was collected from Lingshui, Hainan, China in 2017, and was determined as cryptic species MED using the mtDNA *COI gene* (GenBank accession no. GQ371165) [25]. The whiteflies were reared on tobacco (*Nicotiana tabacum* L.) (breed: NC89 cultivar) in a thermostatic chamber at 27 ± 1 °C with a 16L:8D photoperiod and 60% relative humidity (RH). Before this experiment, the *B. tabaci* MED population had been reared in the laboratory for more than 40 generations.

### 2.2. Identification of HSP Genes from Genome Database

The genome of *B. tabaci* MED, based on the third generation sequencing technology, was generated with the PacBio Sequel Sequencing technology platform (Biomarker Technologies, Beijing, China). Three hundred male and female adult pairs of the *B. tabaci* MED population collected from Lingshui, Hainan, China in 2017 were used as the sequencing samples. Sequenced genes with *Hsp* functions were selected as candidate genes based on the genome annotation library. Afterwards, sequences of all candidate genes were subjected to the National Center for Biotechnology Information (NCBI) BlastP and BlastN and Conserved Domain Database [26] to screen and identify the candidate genes. Finally, Compute pI/Mw tool of ExPASy (https://web.expasy.org/compute_pi/ (accessed on 20 October 2021)) [27] was used to calculate the molecular weight and isoelectric point of the *Hsp*s in this study, and subcellular localization was predicted by CELLOv2.5 (http://cello.life.nctu.edu.tw/ (accessed on 20 October 2021)) [28].

### 2.3. Classification and Nomenclature of HSP Genes

There were 31 *Hsp*s identified and named in previous studies of *B. tabaci.* The sequences of these genes were also obtained in these studies [19,23,24]. The members of *Hsp70*, *Hsp90*, *Hsp60,* and *sHsp* families of *Athalia rosae*, *Drosophila ananassae*, *Nilaparvata lugens*, *Plutella xylostella,* and *Tribolium castaneum* [23] were regarded as the outgroup of the phylogenetic tree of this study. In order to comprehensively identify *B. tabaci* HSP families, BlastP in NCBI was used to find gene sequences of *Hsp40*, *Hsp100,* and other families (except for the HSP genes that had been found previously) [19,23,24]. These sequences were found in the published genomic databases of *A. rosae*, *D. ananassae,* and *N. lugens*. Then, protein sequences of candidate *Hsp*s in this study and previously studied *Hsp*s were aligned by Muscle [29] of MEGA7 with the default option. Subsequently, the neighbor-joining (NJ) method was used to construct the phylogenetic tree of all *Hsp*s in MEGA7 [30] with the following parameters: Poisson correction model, pairwise deletion, and bootstraps test with 1000 replications (random seed) [23]. According to the grouping results in the phylogenetic tree and CDD prediction of each gene in NCBI, the *Hsp*s screened were classified and further named according to their molecular weight [13,31]. The sHSP genes were named according to their molecular weight [32]. The genes in the HSP70 superfamily were divided into *Hsc70* and *Hsp70* according to constitutive and inducible types, respectively [33]. The HSP90 genes were named using the method described for HSP90A and HSP90B to indicate cytosolic and ER HSP90 homologs, respectively [34]. Other family members are directly named after their family names.

### 2.4. Structural Information Prediction and Analysis

The conserved motifs of 22 *Hsp*s were detected by Multiple Em for Motif Elicitation (MEME) an online program [35] with the following parameters: Select the site distribution, Zero or One Occurrence per sequence; Select the number of motifs, 20; How wide can motifs be, 30 to 70 residues for HSP90, HSP70, and HSP60 family members, 10 to 40 for sHSP family members, leave other options as default [23]. Comparing the motif results of complete sequences of candidate genes, those with exactly the same motif composition and sequence were removed as redundant genes. At the same time, this method also verified the accuracy of our preliminary screening and identification. In addition, Gene Structure Display Server (http://gsds.gao−lab.org/ (accessed on 23 October 2021)) [36] was used to graphically portray the numbers and positions of coding sequence (cds)/intron by using gff file of the *B. tabaci* genome. In addition, Clustalw (Multiple Sequence Alignment—CLUSTALW (genome.jp) (accessed on 23 October 2021)), ExPASy (https://swissmodel.expasy.org/interactive (accessed on 23 October 2021)) [37], and ESPript (https://espript.ibcp.fr/ESPript/cgi-bin/ESPript.cgi (accessed on 23 October 2021)) were used to predict the secondary structures (α-helixs and *β*-sheets) of candidate genes. ALN format file and PDB format file were separately obtained using Clustalw and ExPASy, respectively, and then, the two files of each superfamily were separately put into ESPript.

### 2.5. Heat-Stress Treatments

For each experiment replicate, 30 female adults were collected into one ventilated plastic pipette, and were maintained at 42 °C for 3.5 h in a constant-temperature incubator. Thirty adults exposed to 27 °C were included as a control replicate. Each treatment included four biological replicates. Once the experiment finished, treated adults were frozen in liquid nitrogen and stored at −80 °C. Then, the total RNA of 20 surviving adults was extracted for each experiment replicate, using TRIzol Reagent (Thermo Fisher Scientific, Waltham, MA, USA) according to the manufacturer’s protocol. The concentration and purity of extracted RNA (A260/280 and A260/230) were detected by using NanoDrop. RNA samples were stored at −80 °C until needed. PrimeScript RT reagent Kit with gDNA Eraser (Perfect Real Time) (Takara Biotechnology, Qingdao, China) was used to reverse transcribe RNA into cDNA, and RT primer mix of random 6-mers and oligo-dT primer was used to uniformly synthesize all types of cDNA in the sample.

### 2.6. Quantitative Reverse Transcription PCR (RT-qPCR)

The obtained cDNA concentrate was then diluted 30 times and used as a template for RT-qPCR. RT-qPCR reaction system (20 μL): 10 μL TBGreen (Takara Biotechnology, Qingdao, Shandong, China), 7.2μL DEPC water, 0.8 μL primer (10 μM), 2 μL cDNA (30-fold dilution). The RT-qPCR experiment was performed on the JENA qTOWER 2.2 system (Biometra, Analytik Jena, Göttingen, Germany). The reaction procedure was as follows: 95 °C for 30 s, followed by 40 cycles (95 °C for 10 s and 60 °C for 30 s). After the cycling procedure, the temperature was increased from 60 to 95 °C (0.6 °C s^−1^) to obtain the melting curves by promoting denaturation of the double-stranded DNA. The gene-specific primers were designed by primer premier 6, and all primers are listed in Table S1. In each RT-qPCR experiment, each gene was run in three biological replicates with three technical replicates. Two single-copy genes *EF-1α* (forward primer 5′-3′: TAGCCTTGTGCCAATTTCCG; reverse primer 5′-3′: TCCTTCAGCATTACCGTCC) [38] and whitefly *β-actin* (forward primer 5′-3′: TCTTCCAGCCATCCTTCTTG; reverse primer 5′-3′: CGGTGATTTCCTTCTGCATT) [17] were used as endogenous control genes to normalize all data, and the expression level of both control genes was checked at tested temperature.

### 2.7. RNA Interference and Survival Rate Analysis

T7 RNAi Transcription Kit (Vazyme Biotech Co., Ltd., TR102-01, Nanjing, China) was used to synthesize double-stranded RNA (dsRNA) with specific primers. Adults were fed with 20% sucrose with 500 ng/μL dsRNA dilution. A sucrose diet containing 500 ng/μL dsHSP and dsGFP was used for the treatment group and control group, respectively. First, 150 adults were fed on a 20% sucrose diet with dsRNA for 2 days, then 30 adults per replicate were randomly selected for RT-qPCR to detect the interference efficiency, and 80 adults were subjected to 42 °C treatment for 2 h. The same approaches were performed on the negative control. Each treatment was assayed in three replicates. The mortality of adults was counted after high-temperature treatment. After recovery at 25 °C for 1 h, the survival number of adults was counted to ensure that there was no false death among these individuals.

### 2.8. Data Statistical Analysis

Relative expression of double parameters was measured and geometrically averaged [39], and RT-qPCR data were analyzed by 2^−ΔΔCt^ [40]. The data obtained from three independent biological replicates were used to conduct statistical analysis. The survival rate and RT-qPCR data were tested for normality using Kolmogorov−Smirnov test, then the homogeneity of group variances was tested using Levene’s test. All the data followed normal distributions which were analyzed using Student’s t-test using IBM SPSS 21.0 (IBM Corporation, Chicago, IL, USA). The heatmap of gene expression patterns was generated by R (version 4.0.4) package heatmap (1.0.8). Since the expression levels of different genes varied greatly after heat stress, it was difficult to express them clearly in heat maps using original data. Therefore, the original data processed by log function with base 2 was used in heat maps.

## 3. Results

### 3.1. Identification of HSP Genes in the Genome of B. tabaci MED

Thirty three *Hsp*s (*BtaHsps*) were identified based on sequenced genome of *B*. *tabaci* MED (Chu et al., unpublished data) and verified by BlastP, BlastN, and CDD search. Of the 33 *Hsp*s, 22 were newly identified through a method of establishing an unrooted phylogenetic tree and comparing their common motif regions, molecular weight, and other details. Subsequently the newly founded 22 HSP genes were classified into six superfamilies, including sHSP, HSP40, HSP60, HSP70, HSP90, and HSP100, including one sHSP gene, four HSP40 genes, one HSP60 gene, 11 HSP70 genes, four HSP90 genes, and one HSP100 gene. The *B*. *tabaci* HSP genes were named as BtaHSP; the family name was behind it, and then the individual numbers were signed at the end. The detailed information on the *Hsp* such as CDS, molecular weight, isoelectric point, and subcellular localization is shown in Table 1. Most of the HSP genes are located in the cytoplasm and nucleus; a minority are located in the extracellular, ER, plasma membrane, and mitochondria.

### 3.2. Phylogenetic Analysis for Classification Validation

**Figure 1:**
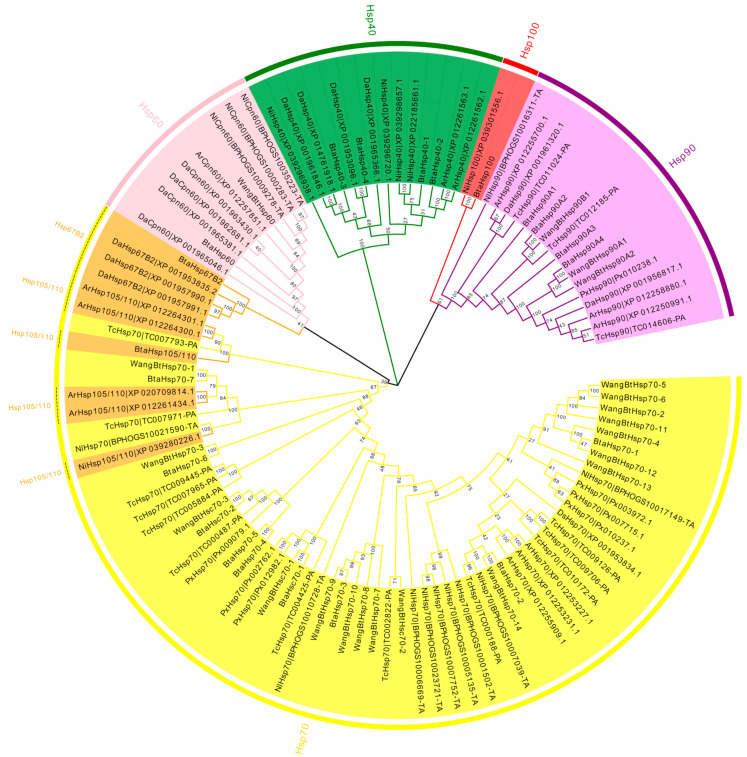
Phylogenetic relationships of *Hsp* from *Bemisia tabaci*, *Plutella xylostella*, *Tribolium castaneum*, *Drosophila ananassae*, *Athalia rosae*, and *Nilaparvata lugens*. The unrooted phylogenetic tree was constructed using MEGA7 by the neighbor-joining method and the bootstrap test was set as 1000 replicates. The colored shadow represents the different *BtaHsp* families.

**Figure 2:**
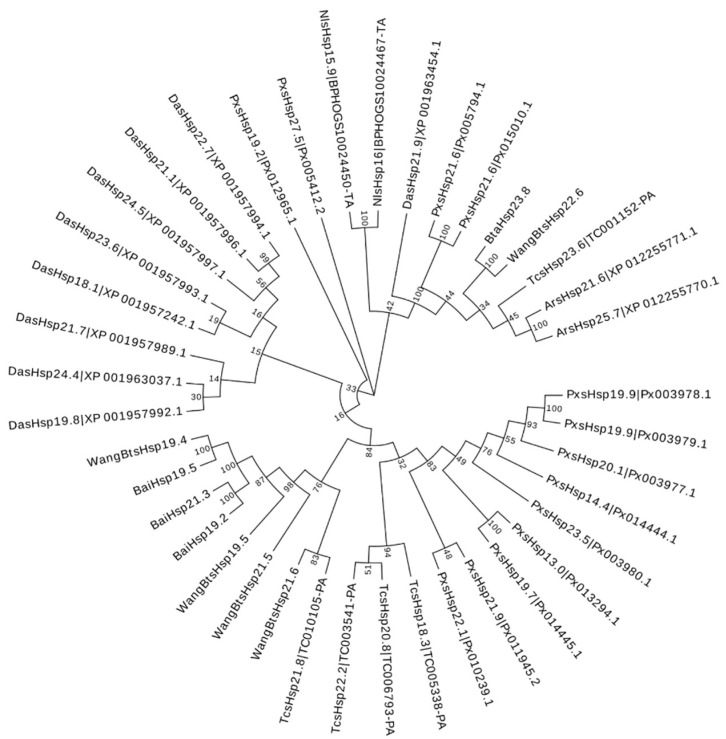
Phylogenetic relationships of *sHsp*s from *Bemisia tabaci*, *Plutella xylostella*, *Tribolium castaneum*, *Drosophila ananassae*, *Athalia rosae*, and *Nilaparvata lugens*. The unrooted phylogenetic tree was constructed using MEGA7 by the neighbor-joining method and the bootstrap test was set as 1000 replicates. The colored shadow represents the different *BtaHsp* families.

**Figure 3:**
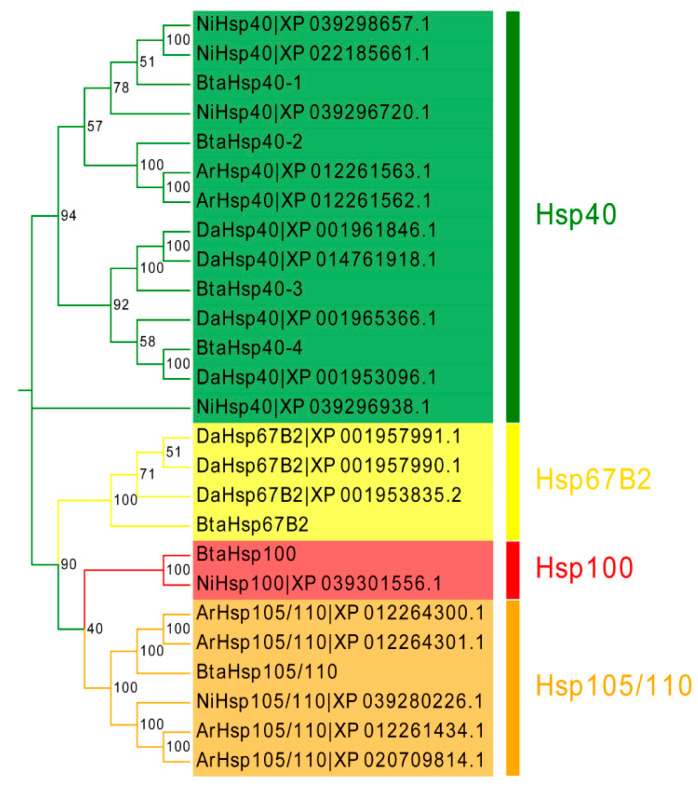
Phylogenetic relationships of *Hsp40*, *Hsp100*, *Hsp105/110*, and *Hsp67B2* from *Bemisia tabaci*, *Drosophila ananassae*, *Athalia rosae*, and *Nilaparvata lugens*. The unrooted phylogenetic tree was constructed using MEGA7 by the neighbor-joining method. The bootstrap test set as 1000 replicates.

**Figure 4:**
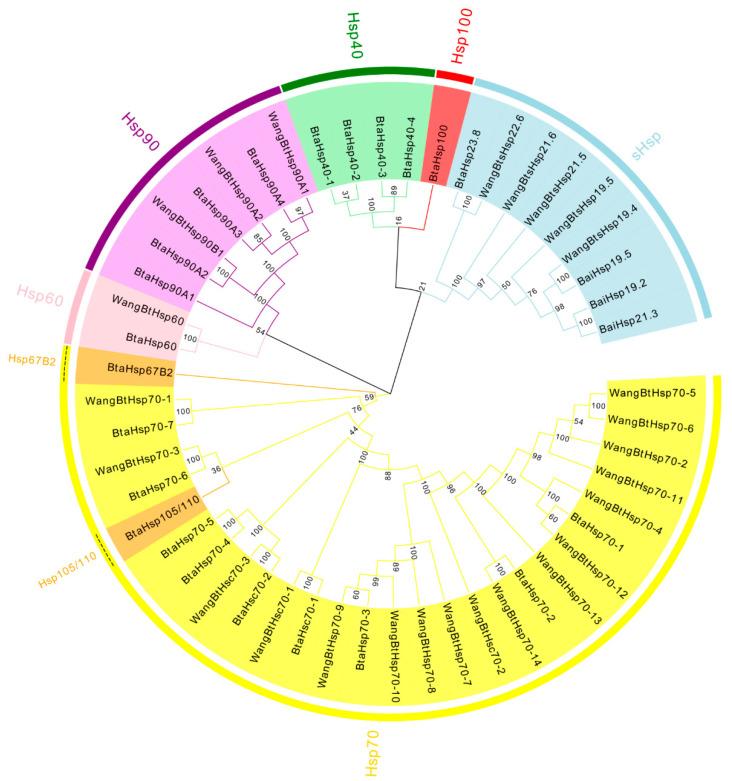
Phylogenetic relationships of 29 *BtHsp* of *Bemisia tabaci* (Wang et al., 2019; Bai et al., 2019) and 22 *BtaHsp*s of this study. The unrooted phylogenetic tree was constructed using MEGA7 by the neighbor−joining method and the bootstrap test was set as 1000 replicates. The colored shadow represents the different *BtaHsp* families.

To assess the phylogenetic relationship between *Hsp*s of *B*. *tabaci* and other outgroup insects, HSP genes of different insects were put together for unrooted phylogenetic trees to further identify our classification result. As shown in Figure 1, all the HSP genes are divided into *sHsp*, *Hsp40*, *Hsp60*, *Hsp70*, *Hsp90*, and *Hsp100*, and they were clustered together to include the HSP family. Since the sHSP family gene sequence is short and the difference is too large compared with other HSP family genes, the phylogenetic tree of *sHsp* was constructed alone (Figure 2). In addition, *Hsp40*, *Hsp100*, *Hsp67B2*, and *Hsp105/110* of *A. rosae*, *D. ananassae,* and *N. lugens* were put together with *B*. *tabaci* to further verify the reliability of classification (Figure 3). This was done to accurately verify that these HSP superfamilies remain conserved in different insects and to verify the accuracy of candidate gene annotations. Then, 29 *BtHsp*s identified by previous studies [23,24] were put together with our 22 *BtaHsp*s to construct the phylogenetic tree (Figure 4). These results are consistent with the previous screening and classification results using genome annotation library and NCBI CDD. At the same time, the maximum-likelihood (ML) method was used to reconstruct the phylogenetic tree of the above content, and the results were consistent with those of the NJ method, proving that our results were reliable (Supplementary Figures S1–S4).

### 3.3. Phylogenetic and Gene Structure Analysis of BtaHSP Genes

**Figure 5:**
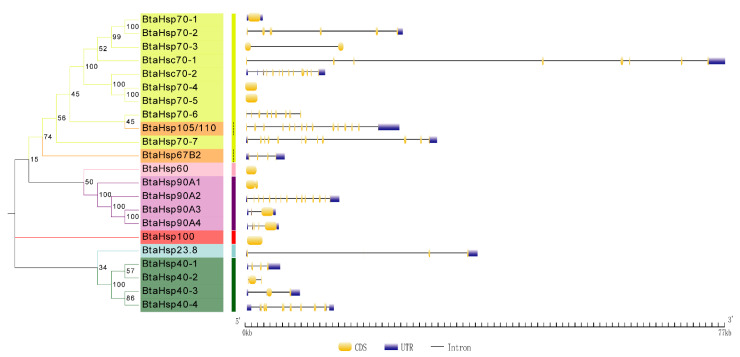
Phylogenetic relationships and gene structures analysis of the *Bemisia tabaci* HSP (BtaHSP) gene superfamily. The unrooted phylogenetic tree was constructed using MEGA7 by the neighbor-joining method and the bootstrap test was set as 1000 replicates. The colored shadow represents the different *BtaHsp* families. CDS/intron structures of BtaHSP genes. The yellow boxes, gray lines, and blue boxes, respectively, represent the cds, intron, and untranslated regions (UTR).

The Gene Structure Display Server (GSDS) was used to portray the gene structure information and this was compared based on the phylogenetic classification. As shown in Figure 5, HSP70, *BtaHsp70-1*, *BtaHsp70-4,* and *BtaHsp70-5* gene have no introns, while *BtaHsp70-2*, *BtaHsp70-3*, *BtaHsp70-6*, and *BtaHsp70-7* have 6, 1, 9, and 11 introns, respectively. Hsc70 genes covered 7 (*BtaHsc70-1*) to 12 (*BtaHsc70-2*) introns. In addition, there were also great differences in genetic structure among members of the HSP90 family and HSP40 family; 1, 14, 2, and 4 introns were contained in *BtaHsp90A1*, *BtaHsp90A2*, *BtaHsp90A3,* and *BtaHsp90A4*, respectively, and 3, 2, 2, and 8 introns were contained in *BtaHsp40-1*, *BtaHsp40-2*, *BtaHsp40-3*, and *BtaHsp40-4*, respectively. 

### 3.4. Phylogenetic and Gene Secondary Structure Analysis of BtaHSP Genes

**Figure 6:**
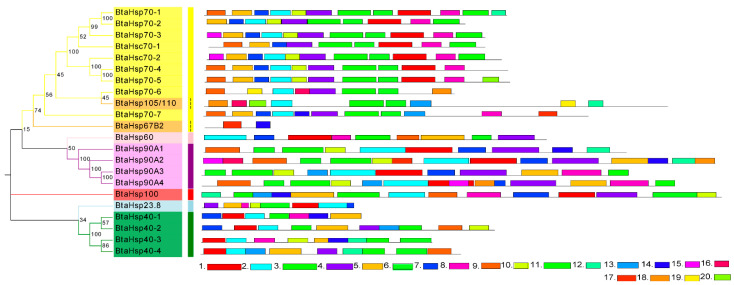
Phylogenetic relationships and protein motif analysis of the *Bemisia tabaci* heat shock protein (BtaHSP) gene superfamily. The unrooted phylogenetic tree was constructed using MEGA7 by the neighbor-joining method and the bootstrap test was set as 1000 replicates. The colored shadow represents the different *BtaHsp* families. The MEME database identified all the motifs with the complete amino acid sequences of *BtaHsp*s. Lengths of each BtaHSP motif were demonstrated proportionally.

The motif and secondary structure were analyzed to obtain the BtaHSP superfamily genes’ structural diversity. Firstly, MEME was used to search 20 motifs in each HSP family of 22 candidate *BtaHSP* genes. Motifs are shown in Figure 6 and also listed in Supplement Data Sheet 1. As displayed in Figure 6, HSP genes from the same superfamily always share similar motifs. The result of motif prediction shows that the distribution of family motifs is relatively conserved. It can also be used to provide a rough indication of the evolutionary relationships between family members in terms of genetic structure.

Furthermore, in order to compare the secondary structure more accurately, four gene families of 29 genes obtained by predecessors were added to each gene family obtained in this study for analysis and comparison. The secondary structure of *BtasHsp*, *BtaHsp40*, *BtaHsp60*, *BtaHsp70,* and *BtaHsp90* families is displayed in Figures S5–S9. There is one *α*-crystalline domain with chaperone function located in the N-terminal, one in the C-terminal region, and six *β*-sheet sandwich structures in each member of *B. tabaci* sHSP family whole region. HSP40s include the C-terminal Zn-finger domain, one highly conserved J domain on N-terminal, and substrate recognition domain on C-terminal. Among them, the J domain contains four *α*-helices, whose ring regions contain highly conserved histidine, proline, and aspartic acid residues. There are two GroEL-like equatorial domains and one GroEL-like apical domain in the BtaHSP60. The HSP70 family has two characteristic domains, one is an ATPase functional domain located at N-terminal, and the other is the C-terminal polypeptide-binding functional domain. The N-terminal ATPase domain consists of two subdomains connected by two *α*-helices with a gap between them. These two subdomains are a tight structure of a *β*-sandwich containing eight *β*-folded chains. The C terminal is an *α*-helical relaxation structure. Like HSP70 chaperones, *BtaHsp105/110*s have an N-terminal nucleotide-binding domain (NBD) and a C-terminal substrate-binding domain (SBD). The *BtaHsp90*s contained a histidine-kinase-like adenosine triphosphatase (ATPase) domain and chaperone motifs in the N-terminal domain and the C-terminal domain, respectively. 

### 3.5. Elevated Differential Expression of BtaHSP Genes at High Temperature

**Figure 7:**
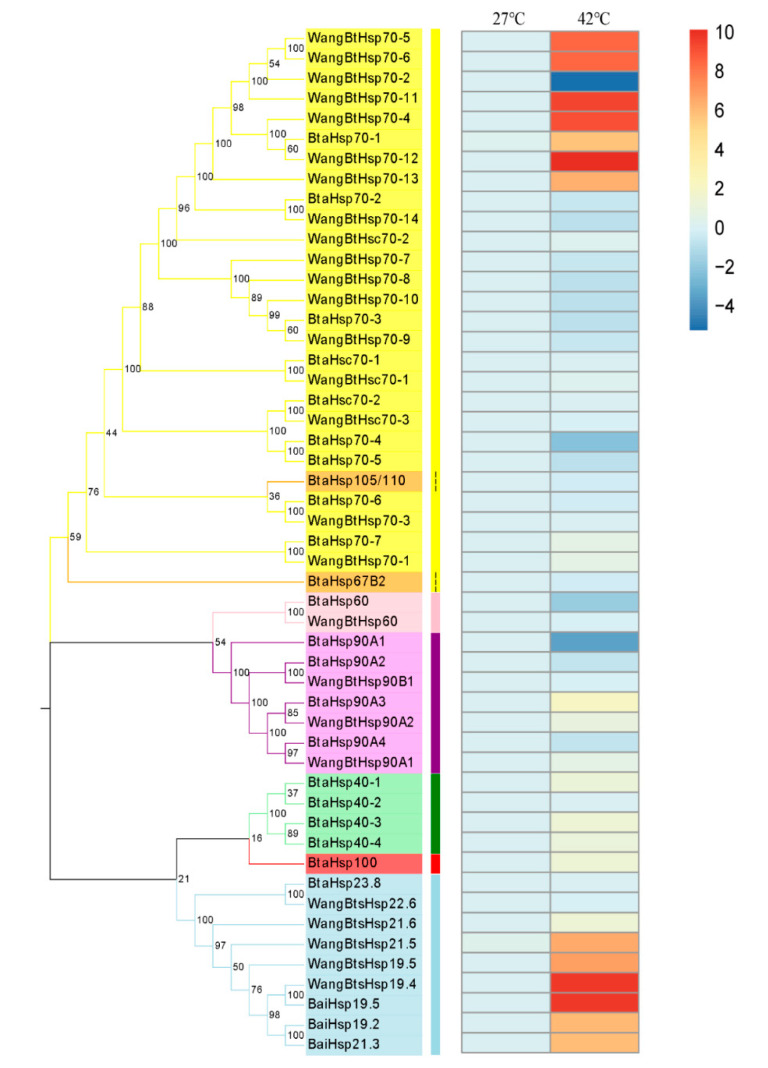
Phylogenetic relationships and gene expression under 42 °C treatment for 3.5 h of the *Bemisia tabaci* HSP (BtaHSP) gene superfamily. The unrooted phylogenetic tree was constructed using MEGA7 by the neighbor-joining method and the bootstrap test was set as 1000 replicates. The colored shadow represents the different HSP families. The heatmap showed the RT-qPCR analysis results of HSP genes in *B. tabaci* subjected to heat stress. The colors of the bar vary from blue to red and represent the scale of relative expression levels. The two columns represent the two processing temperatures, and each row represents one HSP gene member.

The investigation of the expression changes of different HSP genes at high temperature was revealed by RT-qPCR of all the *B*. *tabaci* HSP genes including 29 HSP genes identified by predecessors and 22 HSP genes obtained in this study (Figure 7). Although the time and temperature of heat shock were different from those of previous studies, the expression patterns of the same genes were basically the same after heat shock. Among the 22 HSP genes, the expression of *BtaHsp70-1*, *BtaHsp40-1*, *BtaHsp40-3*, *BtaHsp40-4*, *BtaHsp100,* and *BtaHsp90A3* increased significantly (*p* < 0.05, Student’s *t*-test). In particular, the expression of *BtaHsp70-1* had the highest increase rate (nearly 53 times of the normal state), and the expression of *BtaHsp90A3* had the second highest increase rate (nearly four times of the normal state).

### 3.6. Effect of HSP Gene on High-Temperature Stress

**Figure 8:**
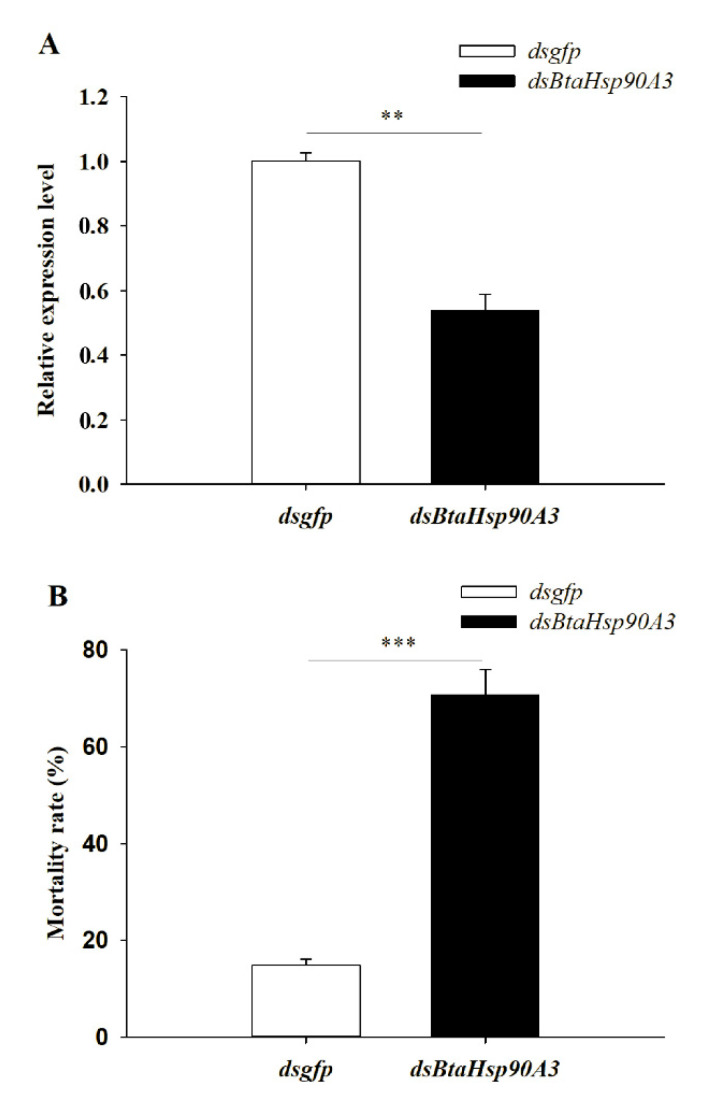
(**A**) RNAi efficiency of *BtaHsp90A3* after feeding double-stranded RNA (dsRNA). (**B**) Survival rates: percentage of surviving adults after 42 °C treatment for 2 h. The survival rate of *Bemisia tabaci* treated with *dsgfp* and *dsBtaHsp90A3* in different treatment groups. Significant differences are calculated by Student’s *t*-test, ** *p* < 0.01, *** *p* < 0.001.

In this study *BtaHsp90A3* was selected as a candidate gene for RNA interference to further determine the role of *BtaHsp* in *B*. *tabaci* high-temperature stress. Changes in gene expression showed that *BtaHsp90A3* could be the gene that responded positively to heat stress, which implies it has a vital function. The *BtaHsp90A3* expression level of *B. tabaci* fed *dsBtaHsp90A3* (0.54 ± 0.049) was significantly lower than that of those fed *dsgfp* (1.00 ± 0.025) (*t*_0.025/4_ = 8.363, *p* < 0.01, Student’s *t*-test) (Figure 8A). Furthermore, the mortality of *B*. *tabaci* fed on *dsBtaHsp90A3* (70.67% ± 5.25%) was significantly higher than that of those fed on *dsgfp* (14.93% ± 1.17%) (*t*_0.025/4_ = 10.383, *p* < 0.001, Student’s *t*-test) (Figure 8B).

## 4. Discussion

### 4.1. Expansion of HSP Gene Superfamily in B. tabaci

Previously, 26 HSP superfamily genes have been identified based on the *B. tabaci* MEAM1 genome [23] and five based on the *B. tabaci* MED samples [19,24]. The sequence of two *Hsp*s reported by Bai et al. (2021) [19] was not disclosed, so it is not discussed in detail. In this study, only 11 of the 31 HSP genes identified in previous studies were found in our *B*. *tabaci* MED genome (Chu et al., unpublished data), but there were also 22 HSP genes of our genome that were different from these 31 genes. The main differences may be associated with the different source of the genome.

In this study, 22 newly found *BtaHsp*s were screened by genome annotation library and comparative genomics. The 22 *Hsp* genes encoding six insect HSP families were identified in the *B*. *tabaci* genome by integrated bioinformatics methods. These results indicate that the number of HSP genes is extended in *B*. *tabaci*. The results show that *Hsp70* is the largest clade of *B*. *tabaci Hsp*, which is consistent with the previous reports that the HSP70 family is one of the main and most abundant HSP families [41]. In addition, we selected *Hsp*s of three species, *A. rosae*, *D. ananassae*, and *N. lugens,* as the outgroup of the phylogenetic tree and used *B*. *tabaci Hsp40*, *Hsp100*, *Hsp67B2,* and *Hsp105/110* for genomic gene blast. Based on the phylogenetic tree, *Hsp105/110* and *Hsp67B2* were found to cluster with HSP70 family genes, which was consistent with *Hsp105/110* belonging to the divergent subgroup of the HSP70 family [42], suggesting that *Hsp67B2* belongs to the HSP70 family. Moreover, the results indicate that *Hsp40* in large numbers exists in the above-mentioned three insect species, while the other three HSP genes are rare and the number is small. This may be caused by gene loss events in the evolution of different species [23]. This phenomenon can be related to previous reports, that HSP70 is the most important and abundant HSP [41] and that HSP40 is necessary for HSP70 to function normally [43]. 

### 4.2. Conserved-Sequence Characteristics of Members of the B. tabaci HSP Superfamily

In general, HSPs from the same family have a relatively high structural identity. The prediction results of the secondary structure showed that HSPs in the same family have similar motifs. Different types of HSPs have different domains, which play important roles in their function. As the earliest family to extend from the evolutionary branch [23], the HSP70 family has the longest evolutionary history and may be more complex in structure. Three of our nine HSP70 genes have no introns, which are typical structures in prokaryotic genes [44], and introns in eukaryotes are gradually transitioned from prokaryotic to eukaryotic [45]. This suggests that the evolution and expansion of the HSP70 family in *B*. *tabaci* has already occurred.

HSP70 contains one highly conserved 44 kDa ATPase domain at the N-terminal and one 25 kDa domain at the C-terminal [46]. Furthermore, C-terminal motifs vary with subcellular localization [47]. It has been reported that *WangBtHsp70-1* and *WangBtHsp70-3* are isolated from other *Hsp70* in the phylogenetic tree due to the absence of any known specific motif in the C-terminal [23]. When observing the motifs of the HSP70 family in the phylogenetic tree constructed from 22 BtaHSPs identified in this study, *BtaHsp105/110*, *BtaHsp67B2*, and *BtaHsp70-7* were also found to be isolated from other HSP70 due to the absence of any known specific motifs at the C-terminus. HSP40 consists of a J domain, G/F domain, zinc-finger domain, and hydroxyl terminal region. HSP40 forms a heterogeneous family whose members have at least a conserved J-domain [48,49]. The HPD motif is present in all J domains known so far [50,51]. The characteristic motif of zinc-finger structure is XX-cysteine-x-glycine-x-glycine (CXXCXGXGX), and the presence of a zinc-finger structure is the basis of HSP40 classification [52]. HSP40 was identified by an HPD motif located at the N-terminal, while classification was dependent upon the presence of a zinc-finger motif and a G/F domain [52]. Taken together, either the N-terminus or the C-terminus motif can be used for the identification and classification of genes of *B*. *tabaci* HSP superfamily. From the above information, we can propose that the differentiation and conservation of gene structure are closely related to the evolution and expansion of the *B*. *tabaci* HSP gene family.

### 4.3. Specific BtaHSP Genes Are Important in Coping with Temperature Stress

Previous studies have demonstrated that HSPs contribute to temperature tolerance, and our study obtained 22 BtaHSPs. Therefore, we obtained the expression pattern of the HSP gene superfamily of *B*. *tabaci* under heat stress by RT-qPCR. HSP70 is a highly conserved protein that acts as a molecular chaperone, and it is a strong indicator of the response of *B*. *tabaci* to heat shock [41,53]. The results showed that most BtaHSP70 genes were induced by heat stress, which was consistent with reported results [23]. As a helper protein of HSP70, HSP40 can promote the ATPase activity of HSP70 [43]. It is speculated that HSP40 is necessary for HSP70 to function properly. Both *Hsp70* and *Hsp40* are highly conserved, and have high homology in different species [54], so such a mechanism of action is likely to apply to *B*. *tabaci*. Our results showed that most HSP40 genes of *B. tabaci* were induced by heat stress. Although the specific regulatory role of *B*. *tabaci* HSP40 on HSP70 is still unclear, in the current study most HSP40s and HSP70 was induced by high temperature. However, members of the *Hsc70* family have been reported not to respond to heat stress [55], which is consistent with the description of *Hsc70* in the current study.

The expression of *BtaHsp70-1* was highest among the candidate genes (nearly 53 times of the normal state). The function of *BtaHsp70-1* had been well-studied previously [56]. Therefore, the expression of *BtaHsp90A3* (the second-highest increase rate, nearly four times of the normal state) was chosen for the RNA interference test to further study the role of BtaHSP in *B*. *tabaci* under high-temperature stress. After silencing of *BtaHsp90A3*, the heat tolerance of *B. tabaci* decreased with the decrease of the expression level of the gene, which indicated that *BtaHsp90A3* contributes to temperature tolerance.

As the most conserved gene in the HSP family, HSP70 has been extensively studied [19]. Studies have shown that the HSP70 members have the function of receiving the denatured protein caused by stress and then transporting it to HSP90 for repair to complete the resisting process [57]. HSP90 is highly conserved in evolution, and plays a crucial role in different cellular processes, especially reflected in signal transduction and gene transcription [58,59]. Furthermore, HSP90 interacts with intermediates at later stages than HSP70 to make the protein-folding process complete [60]. Therefore, it can be speculated that HSP70 requires HSP90 for complete functionality. No studies on RNA interference in the HSP90 family of *B. tabaci* MED have been reported. Therefore, this study provides a new idea for the prevention and control of *B. tabaci* MED by targeting the RNAi of the HSP90 family gene.

## 5. Conclusions

The 22 *Hsp*s identified in this study were verified by phylogenetic tree construction and secondary structure prediction analysis. Furthermore, we have confirmed that the secondary structure of the genes is closely related to their evolutionary relationships. In this study, we linked the response of these genes, especially *BtaHsp90A3* of the HSP90 family, to heat stress. Therefore, it can be inferred that searching for genes related to Hsp70 function from the above-mentioned members of the HSP family is feasible. It can provide new ideas about the regulation mechanism of HSP gene expression in *B*. *tabaci*.

## Figures and Tables

**Table 1:** Information on HSP genes superfamily in *Bemisia tabaci*.

Family	Gene Identifier	Gene Name	CDS	Mw (kDa)	pI	Subcelluar Location	Strand
sHSP	OM176688	*BtaHsp23.8*	91–720	23.8	6.92	Extracellular/nuclear	Plus
HSP40	OM176682	*BtaHsp40-1*	202–957	28.9	6.55	Nuclear	Plus
	OM176693	*BtaHsp40-2*	1–1350	50.2	9.31	Nuclear	Plus
	OM176684	*BtaHsp40-3*	232–1284	38.8	9.11	Nuclear	Plus
	OM176675	*BtaHsp40-4*	380–1579	44.4	6.35	Nuclear	Plus
HSP60	OM176680	*BtaHsp60*	1–1668	58.9	5.38	Cytoplasmic/nuclear	Plus
HSP70	OM176673	*BtaHsp70-1*	228–2180	72	8.22	Mitochondrial/nuclear	Plus
	OM176691	*BtaHsp70-2*	7–1659	60.7	5.6	Cytoplasmic/nuclear	Plus
	OM176686	*BtaHsp70-3*	1–1776	65.7	5.92	Cytoplasmic/nuclear	Plus
	OM176692	*BtaHsc70-1*	1–1758	65.3	5.21	ER/nuclear	Plus
	OM176674	*BtaHsc70-2*	394–2253	67.5	5.3	Nuclear	Plus
	OM176678	*BtaHsp70-4*	1–1908	68.7	4.94	Cytoplasmic/nuclear	Plus
	OM176681	*BtaHsp70-5*	1–1926	70.7	5.5	Cytoplasmic/nuclear	Plus
	OM176685	*BtaHsp70-6*	1–1572	57.6	5.57	Plasma membrane	Plus
	OM176676	*BtaHsp70-7*	207–2618	89.9	5.43	Cytoplasmic/nuclear	Plus
	OM176689	*BtaHsp105/110*	1–2904	107.6	5.32	Cytoplasmic/ER	Plus
	OM176683	*BtaHsp67B2*	420–917	18.6	8.91	Mitochondrial	Plus
HSP90	OM176677	*BtaHsp90A1*	1–1902	72.4	5.11	Cytoplasmic/nuclear	Plus
	OM176694	*BtaHsp90A2*	135–2462	88.7	5.09	Cytoplasmic/cytoskeletal/nuclear	Plus
	OM176687	*BtaHsp90A3*	308–2242	74.2	5.07	Cytoplasmic/nuclear	Plus
	OM176690	*BtaHsp90A4*	207–2402	84	5.01	Cytoplasmic/nuclear	Plus
HSP100	OM176679	*BtaHsp100*	1–2472	92.4	5.96	Cytoplasmic	Plus

## Data Availability

The data presented in this study are available within the article and Supplementary Materials.

## Supplementary Materials

The following supporting information can be downloaded at: https://www.mdpi.com/article/10.3390/insects13070570/s1, Figure S1: Phylogenetic relationships of *Hsp*s from *Bemisia tabaci*, *Plutella xylostella*, *Tribolium castaneum*, *Drosophila ananassae*, *Athalia rosae*, and *Nilaparvata lugens.* Figure S2: Phylogenetic relationships of *sHsp*s from *Bemisia tabaci*, *Drosophila ananassae*, *Athalia rosae*, and *Nilaparvata lugens.* Figure S3: Phylogenetic relationships of *Hsp40*, *Hsp100*, *Hsp105/110*, *Hsp67B2* from *Bemisia tabaci*, *Drosophila ananassae*, *Athalia rosae*, and *Nilaparvata lugens.* Figure S4: Phylogenetic relationships and gene structures analysis of the *Bemisia tabaci* HSP (BtaHSP) gene superfamily. Figures S5−S9: The secondary structures of *Bemisia tabaci* sHSPs, HSP40s, HSP60s, HSP70s, and HSP90s. Supplementary Data Sheet 1: Sequence logos for the conserved motifs of sHSPs in the *Bemisia tabaci BtasHsp* motif. Supplementary Data Sheet 2: Information of *Hsp* sequences in the six surveyed species. Table S1: Gene-specific primers for RT-qPCR used in this study.
