# Supplementary material for: Genome-Wide Identification and Analysis of the Heat-Shock Protein Gene Superfamily in Bemisia tabaci and Expression Pattern Analysis under Heat Shock
Source: Insects. 2022 Jun 23;13(7):570. doi: 10.3390/insects13070570 (PMC9319060; doi:10.3390/insects13070570)
Supplement: Supplementary file 1 [file insects-13-00570-s001.zip › insects-1777694-supplementary.pdf]

Supplementary Information

Genome-wide identification and analysis of Hsp gene superfamily in *Bemisia tabaci* and expression patterns analysis under heat shock

Hao-Yuan Zheng[1], Peng-Hao Qin[1], Kun Yang[1], Tong-Xian Liu[1], You-Jun Zhang[2] and Dong Chu[1,*]

1 Key Lab of Integrated Crop Pest Management of Shandong Province, College of Plant Health and Medicine, Qingdao Agricultural University, Qingdao, 266109, China.

2 Department of Plant Protection, Institute of Vegetables and Flowers, Chinese Academy of Agricultural Sciences, Beijing, 100081, China.

Running title: Identification and analysis of *Bemisia tabaci* HSP genes

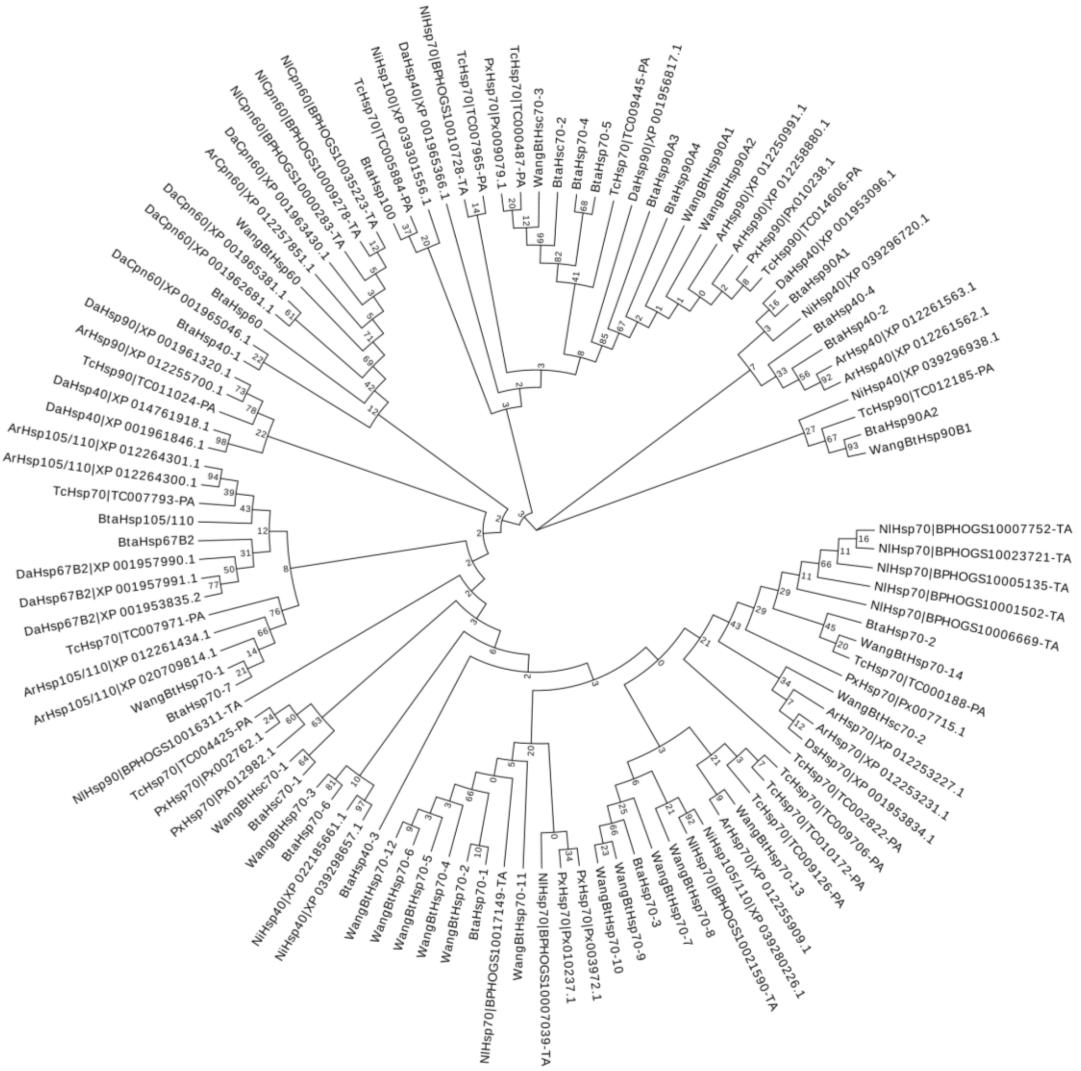

Supplementary Figure S1. Phylogenetic relationships of *Hsp*s from *Bemisia tabaci*, *Plutella xylostella*, *Tribolium castaneum*, *Drosophila ananassae*, *Athalia rosae*, and *Nilaparvata lugens*. The unrooted phylogentic tree was constructed using MEGA7 by Maximum Likelihood method with JTT model. The bootstrap test was set as 1000 replicates.

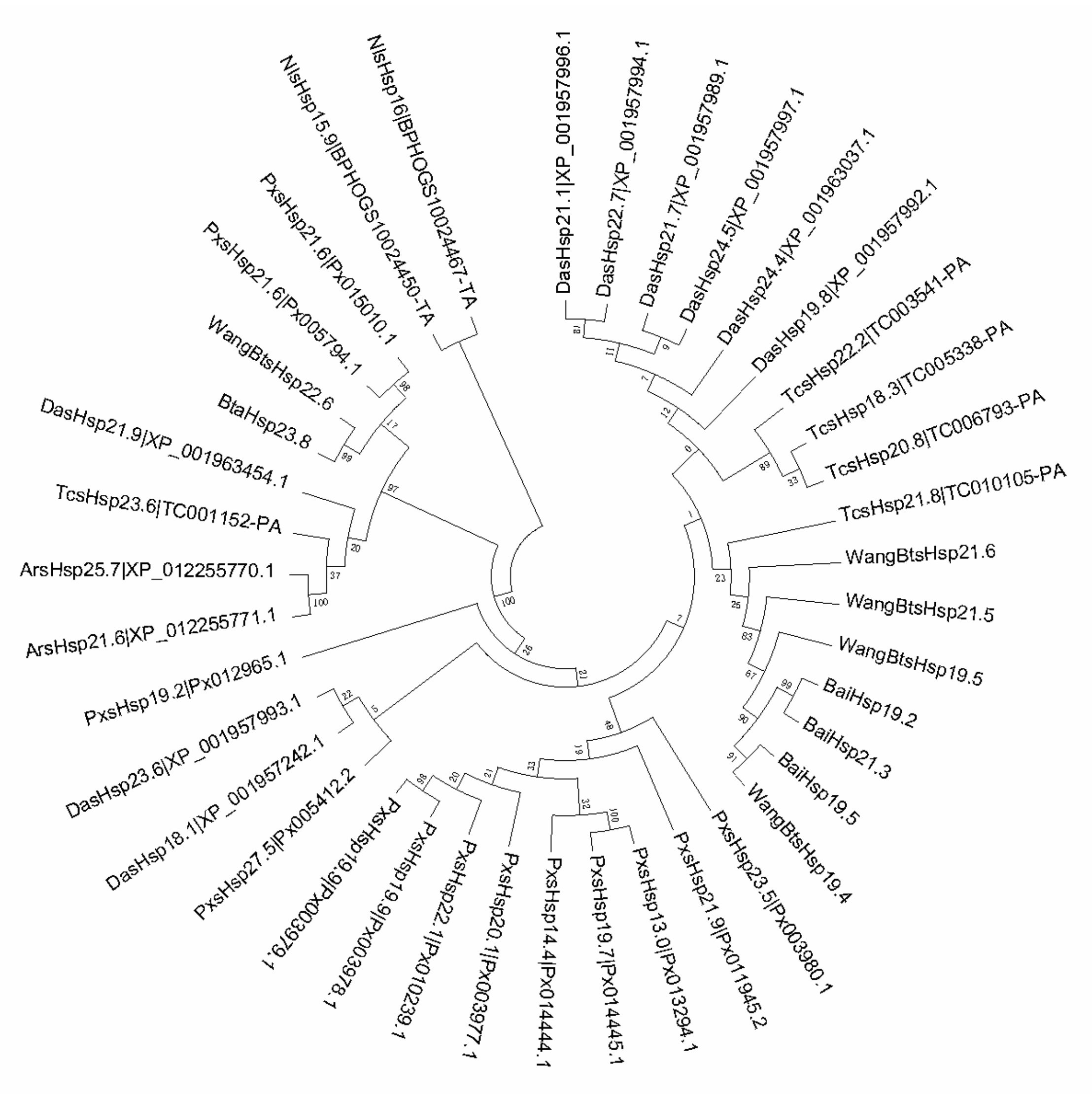

Supplementary Figure S2. Phylogenetic relationships of *sHsp*s from *Bemisia tabaci, Plutella xylostella, Tribolium castaneum, Drosophila ananassae, Athalia rosae,* and *Nilaparvata lugens*. The unrooted phylogentic tree was constructed using MEGA7 by Maximum Likelihood method with JTT model. The bootstrap test was set as 1000 replicates.

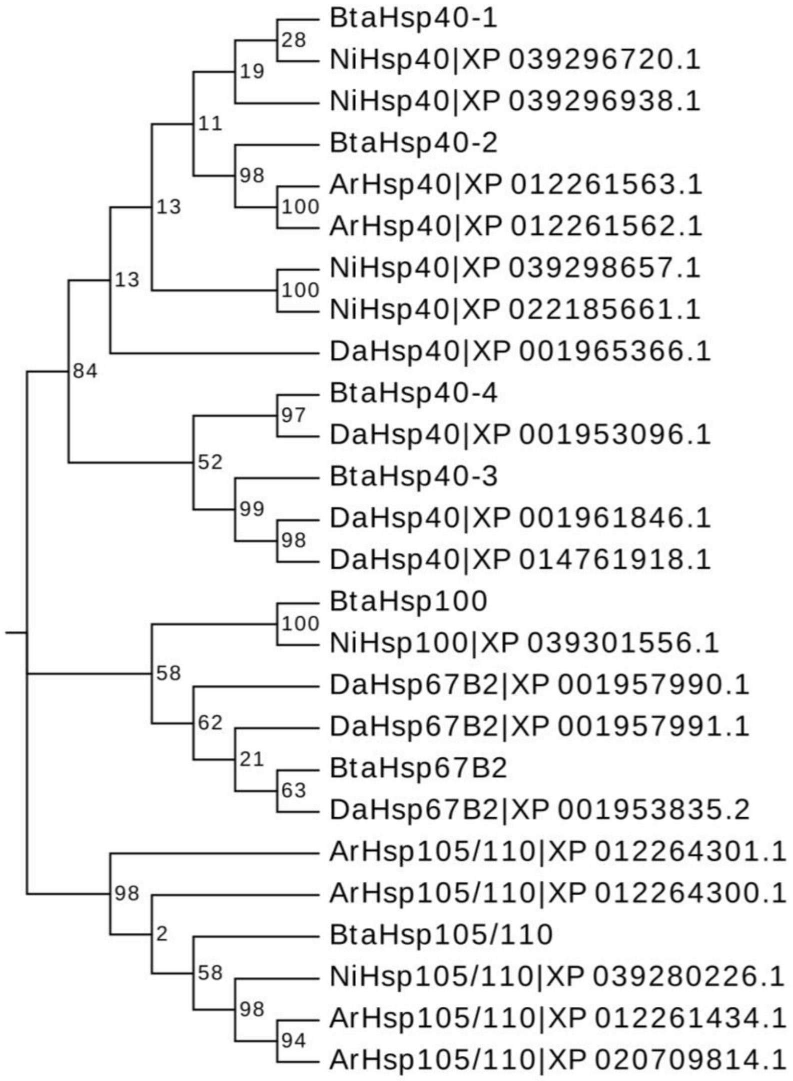

Supplementary Figure S3. Phylogenetic relationships of *Hsp40, Hsp100, Hsp105/110, Hsp67B2* from *Bemisia tabaci, Drosophila ananassae*, *Athalia rosae*, and *Nilaparvata lugens*. The unrooted phylogentic tree was constructed using MEGA7 by Maximum Likelihood method with JTT model. The bootstrap test was set as 1000 replicates.

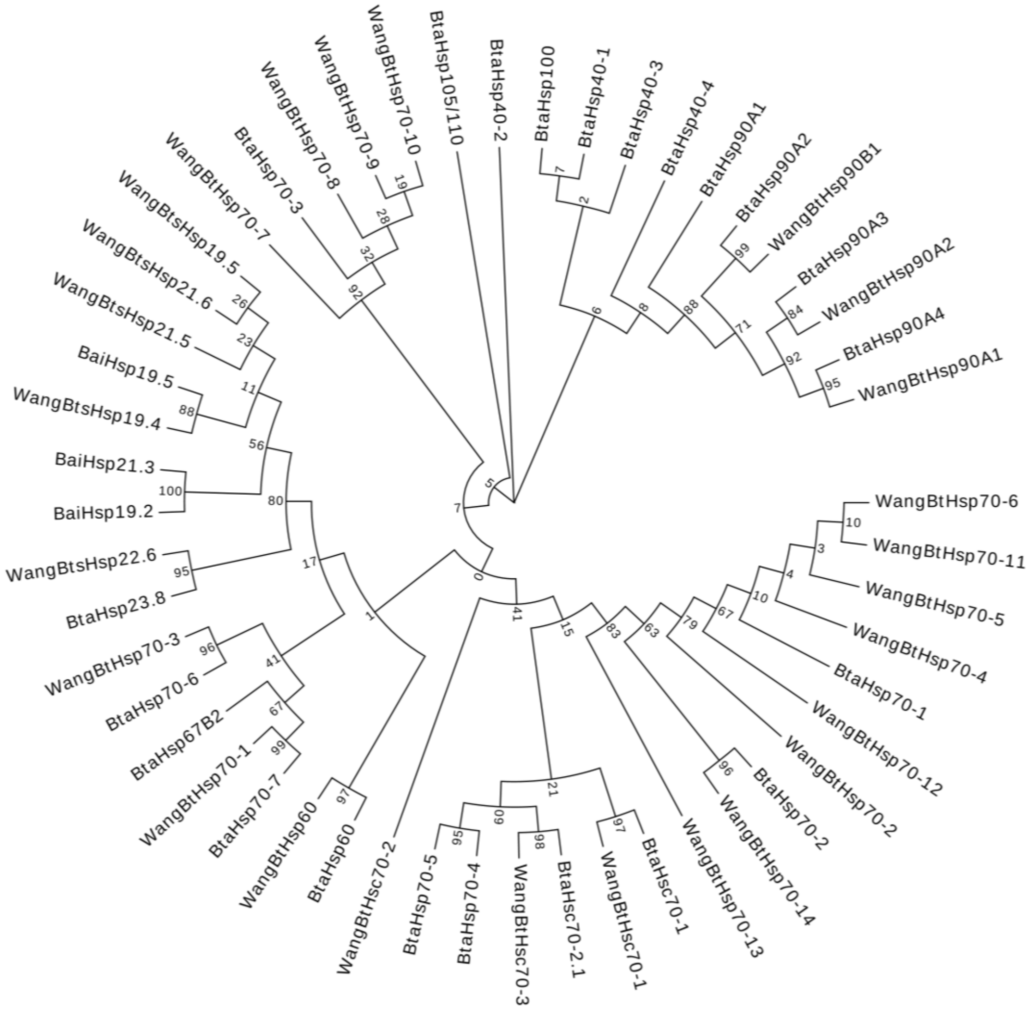

Supplementary Figure S4. Phylogenetic relationships and gene structures analysis of the *Bemisia tabaci* HSP (BtaHSP) gene superfamily. The unrooted phylogentic tree was constructed using MEGA7 by Maximum Likelihood method with JTT model. The bootstrap test was set as 1000 replicates.

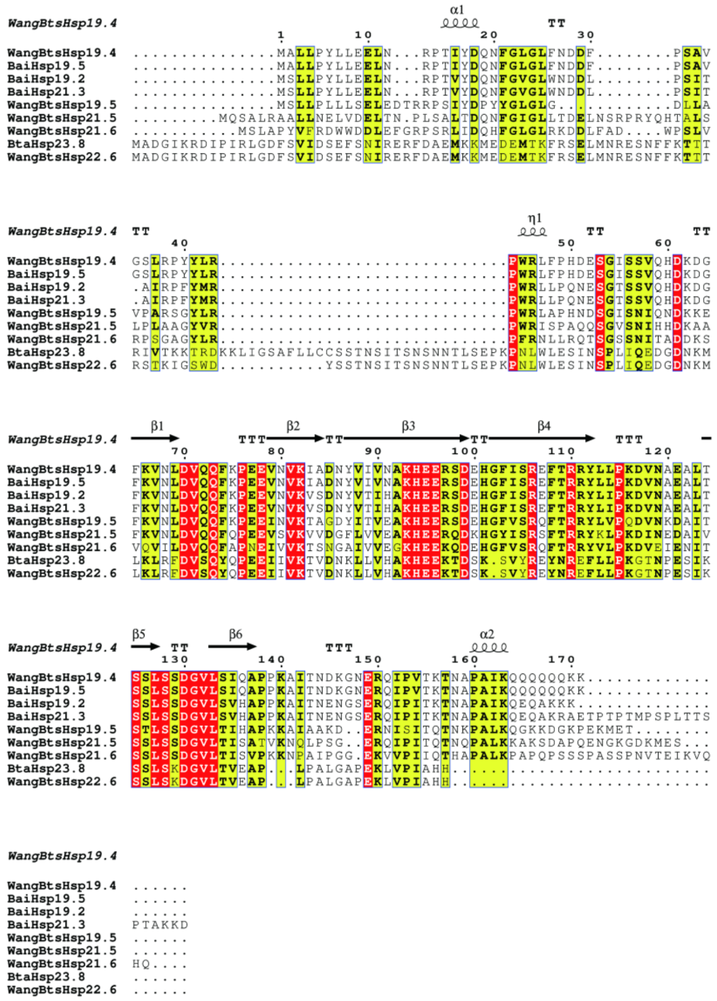

Supplementary Figure S5. The secondary structures of *Bemisia tabaci* sHSPs. α-helices and β-sheets were represented by yellow boxes and blue arrows, respectively. The conserved domain of sHSPs were marked with same color shadow under the sequences.

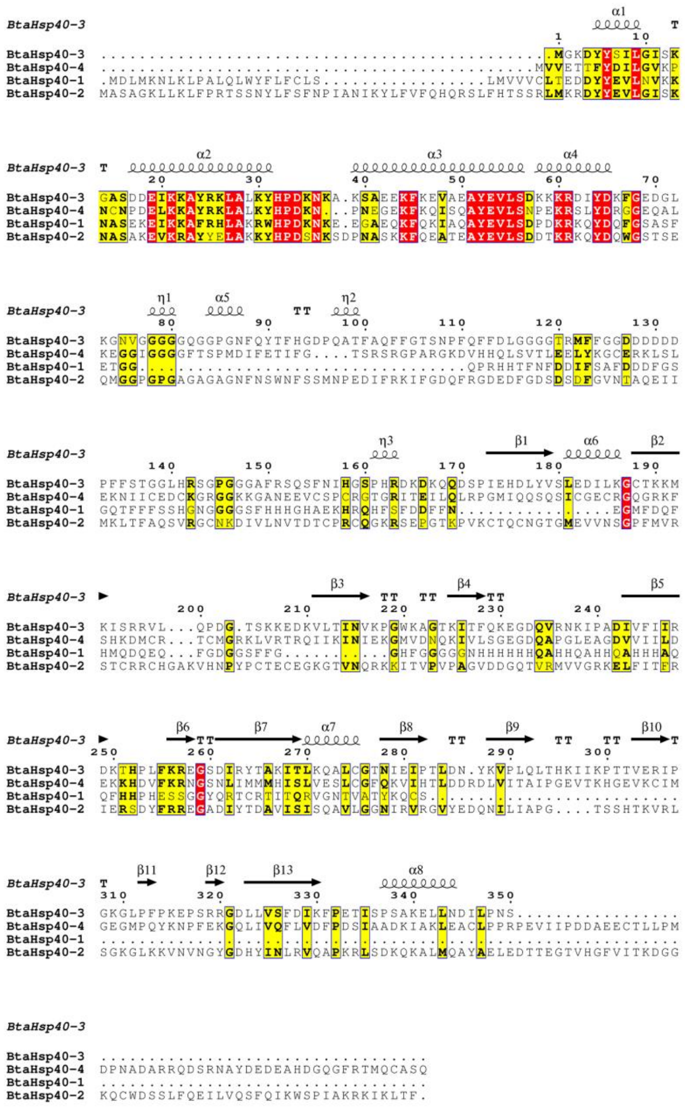

Supplementary Figure S6. The secondary structures of *Bemisia tabaci* HSP40s. α-helices and β-sheets were represented by yellow boxes and blue arrows shown above the sequence, respectively. The conserved domain of HSP40s were marked with a line under the sequences.

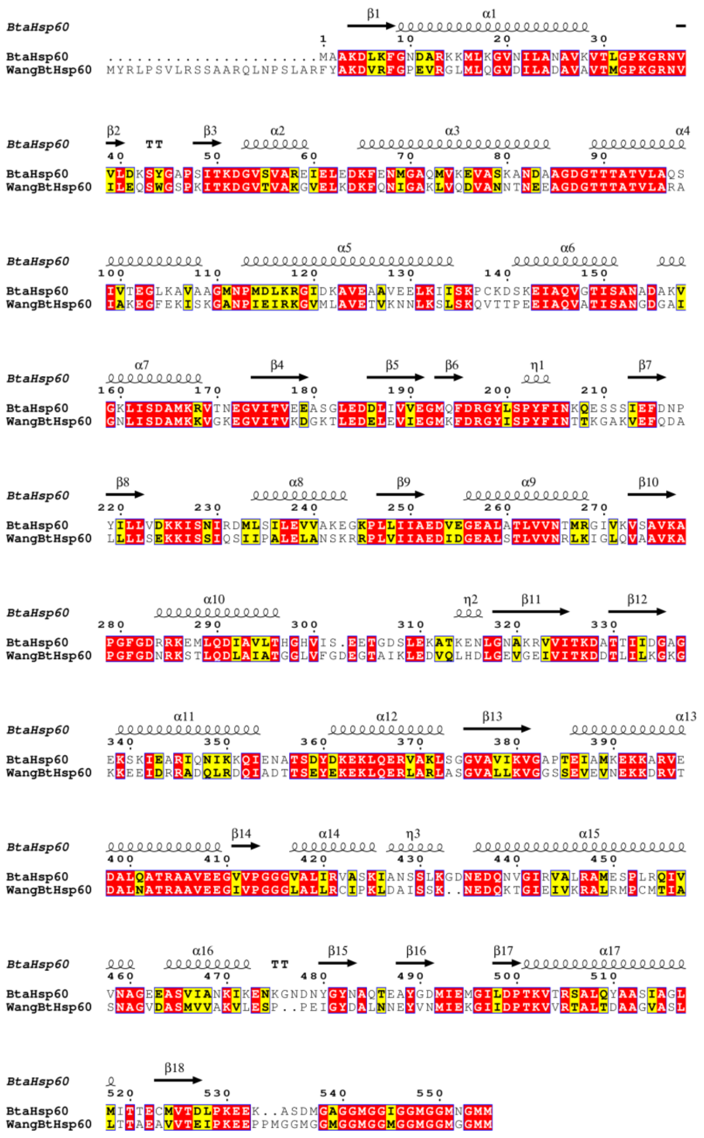

Supplementary Figure S7. The secondary structures of *Bemisia tabaci* HSP60s. α-helices and β-sheets were represented by yellow boxes and blue arrows shown above the sequence, respectively. The conserved domain of Hsp60 proteins were marked with a line under the sequences.

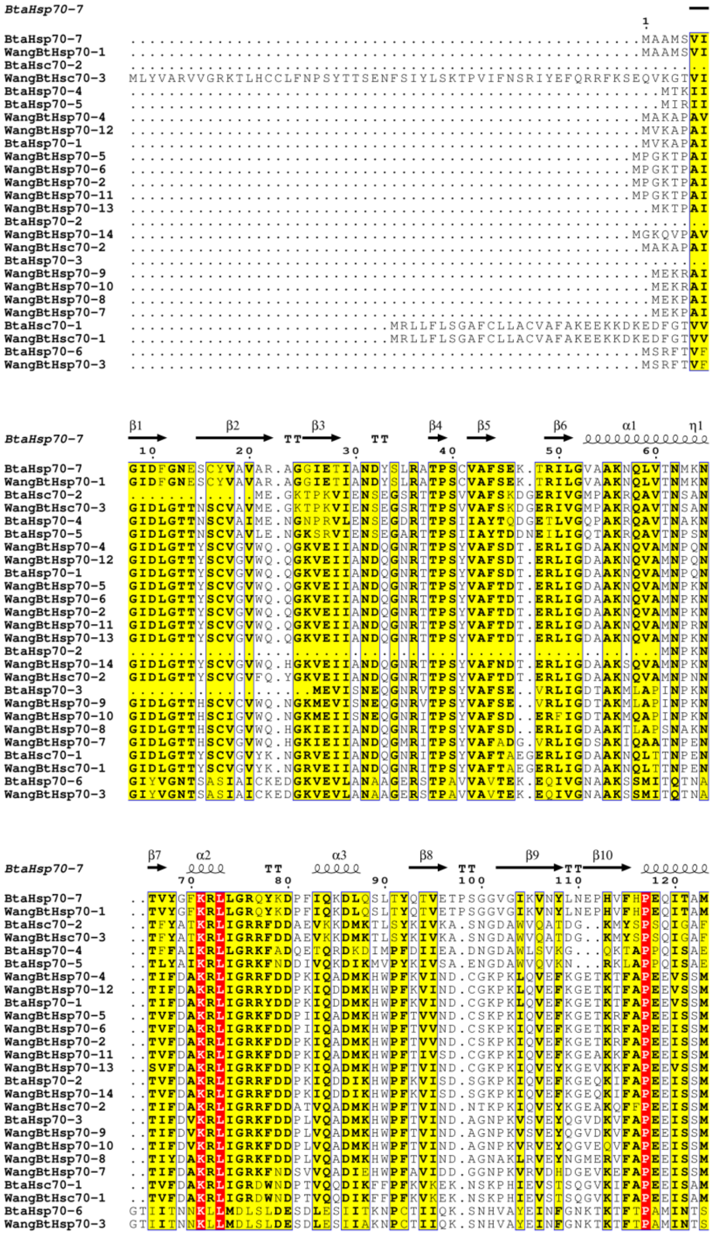

BtaHsp70-7
BtaHsp70-7
WangBtHsp70-1
BtaHsc70-2
WangBtHsc70-3
BtaHsp70-4
BtaHsp70-5
WangBtHsp70-4
WangBtHsp70-12
BtaHsp70-1
WangBtHsp70-5
WangBtHsp70-6
WangBtHsp70-2
WangBtHsp70-11
WangBtHsp70-13
BtaHsp70-2
WangBtHsp70-14
WangBtHsc70-2
BtaHsp70-3
WangBtHsp70-9
WangBtHsp70-10
WangBtHsp70-8
WangBtHsp70-7
BtaHsc70-1
WangBtHsc70-1
BtaHsp70-6
WangBtHsp70-3
β1 β2 β3 β4 β5 β6 α1 η1
β7 α2 α3 β8 β9 β10

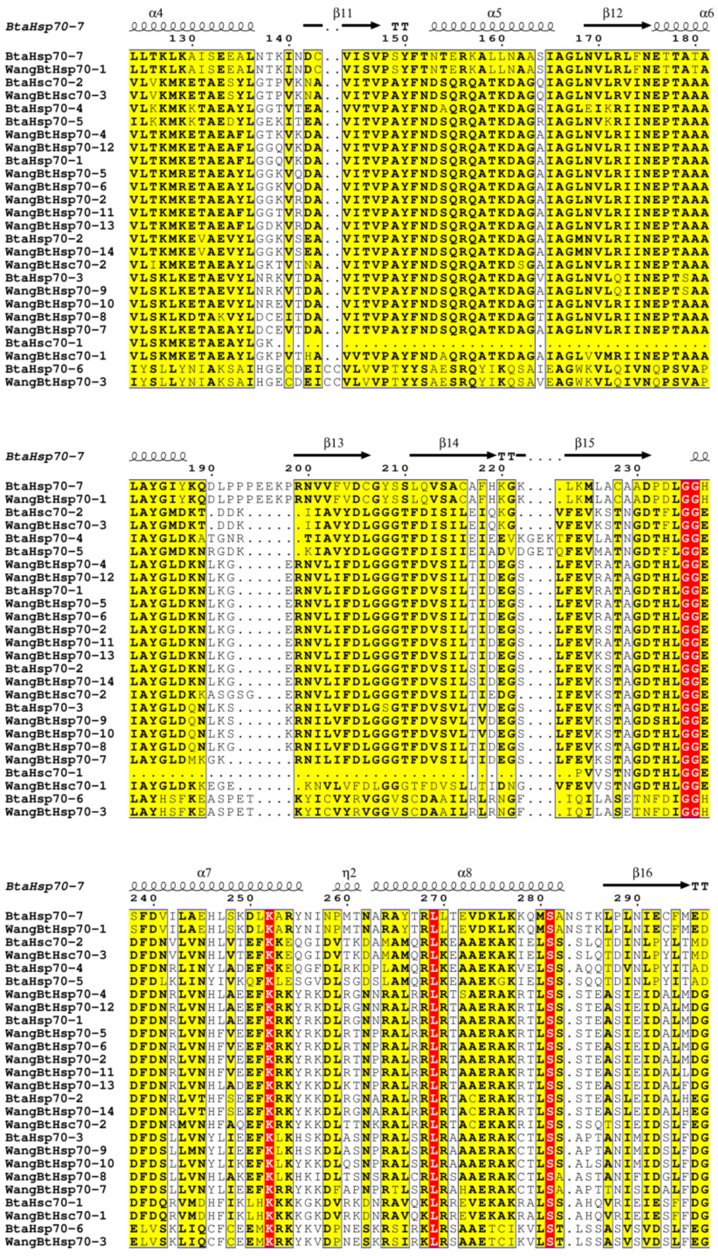

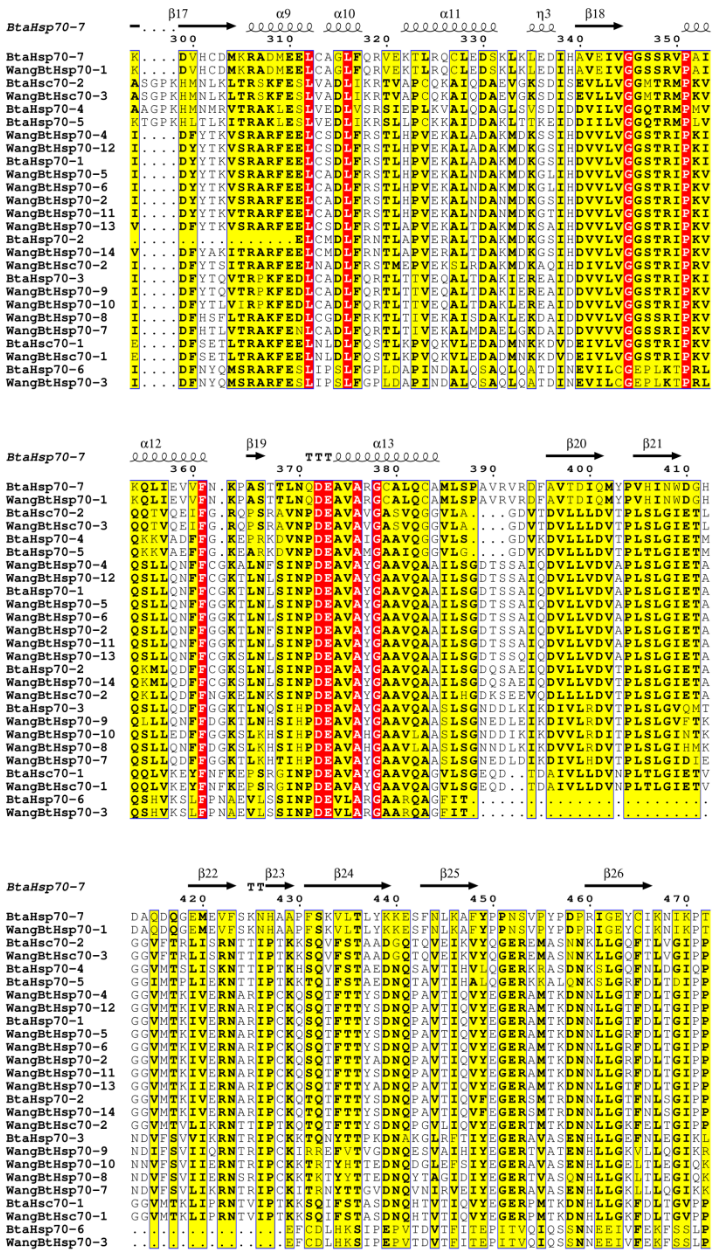

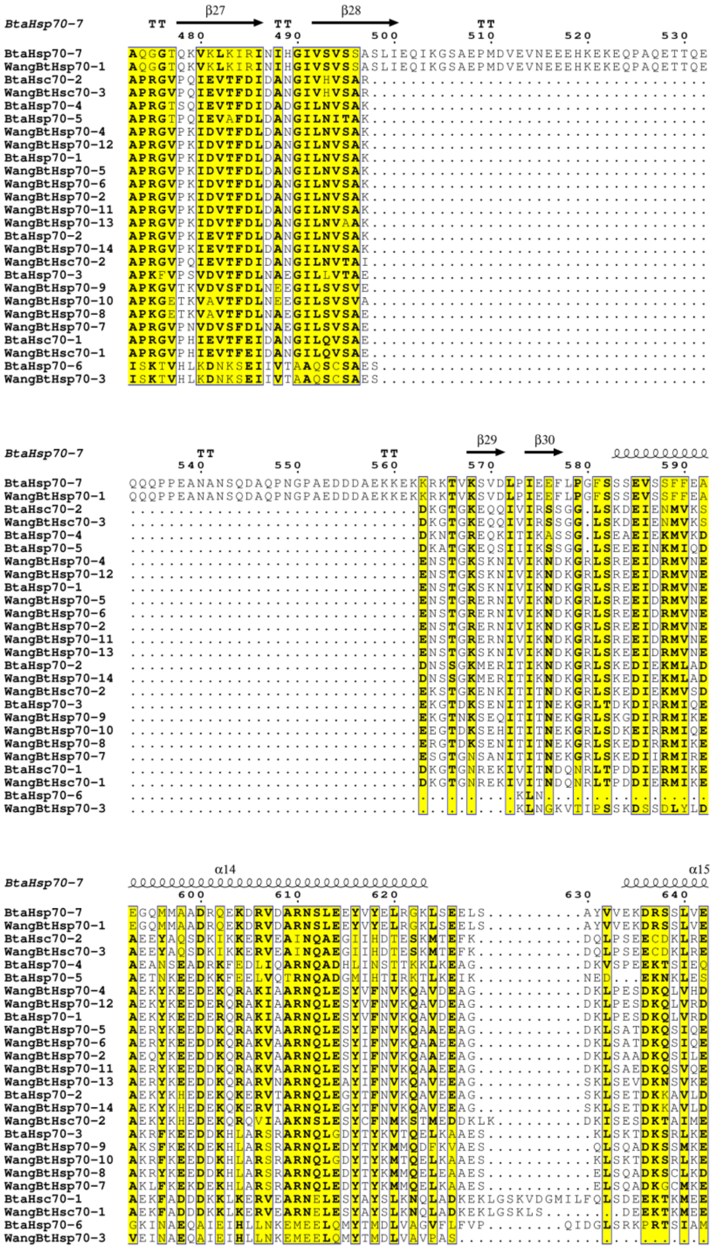
BtaHsp70-7
β27
β28
β29
β30
α14
α15
BtaHsp70-7
WangBtHsp70-1
BtaHsc70-2
WangBtHsc70-3
BtaHsp70-4
BtaHsp70-5
WangBtHsp70-4
WangBtHsp70-12
BtaHsp70-1
WangBtHsp70-5
WangBtHsp70-6
WangBtHsp70-2
WangBtHsp70-11
WangBtHsp70-13
BtaHsp70-2
WangBtHsp70-14
WangBtHsc70-2
BtaHsp70-3
WangBtHsp70-9
WangBtHsp70-10
WangBtHsp70-8
WangBtHsp70-7
BtaHsc70-1
WangBtHsc70-1
BtaHsp70-6
WangBtHsp70-3

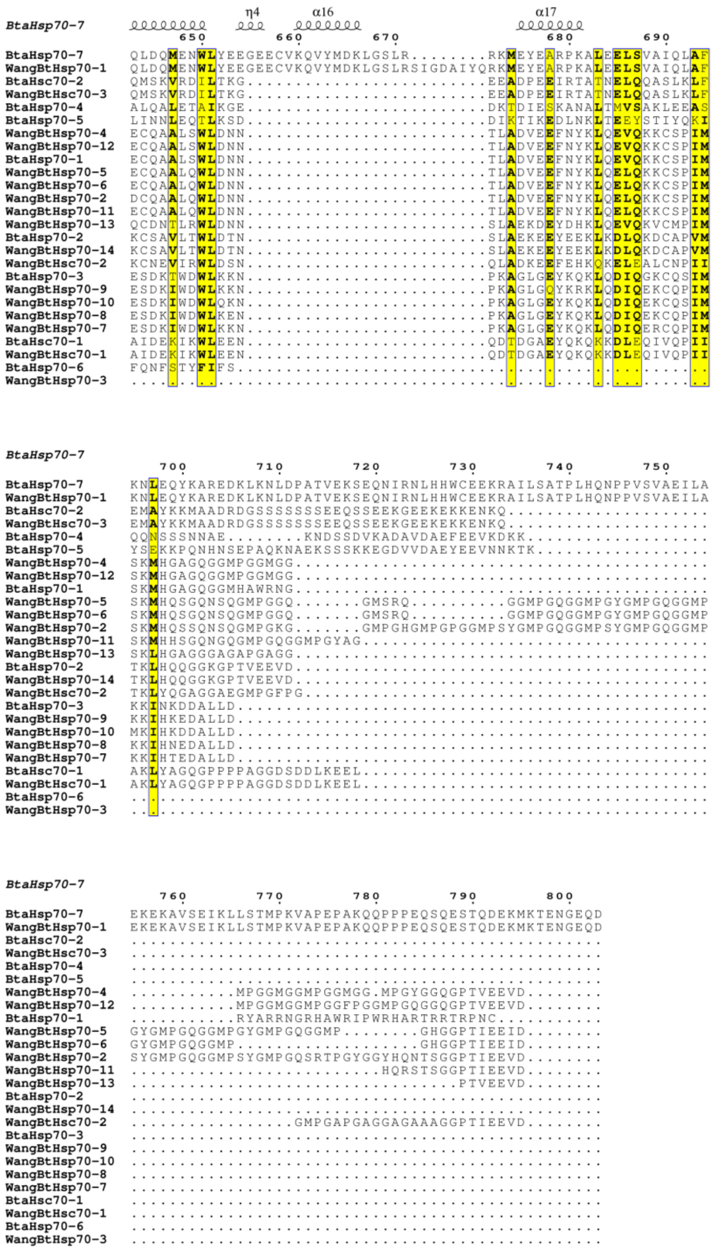

Supplementary Figure S8. The secondary structures of *Bemisia tabaci* HSP70s. α-helices and β-sheets were represented by yellow boxes and blue arrows shown above the sequence, respectively. The conserved domain of HSP70s were marked with a line under the sequences.

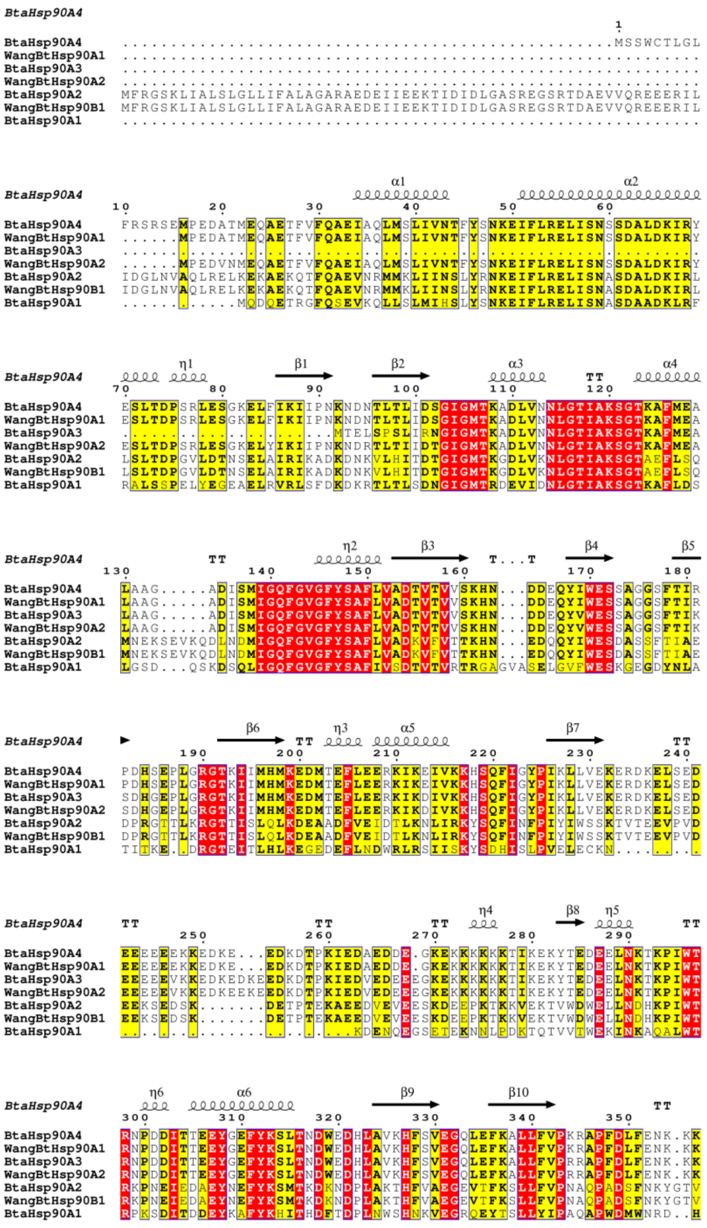
BtaHsp90A4
WangBtHsp90A1
BtaHsp90A3
WangBtHsp90A2
BtaHsp90A2
WangBtHsp90B1
BtaHsp90A1

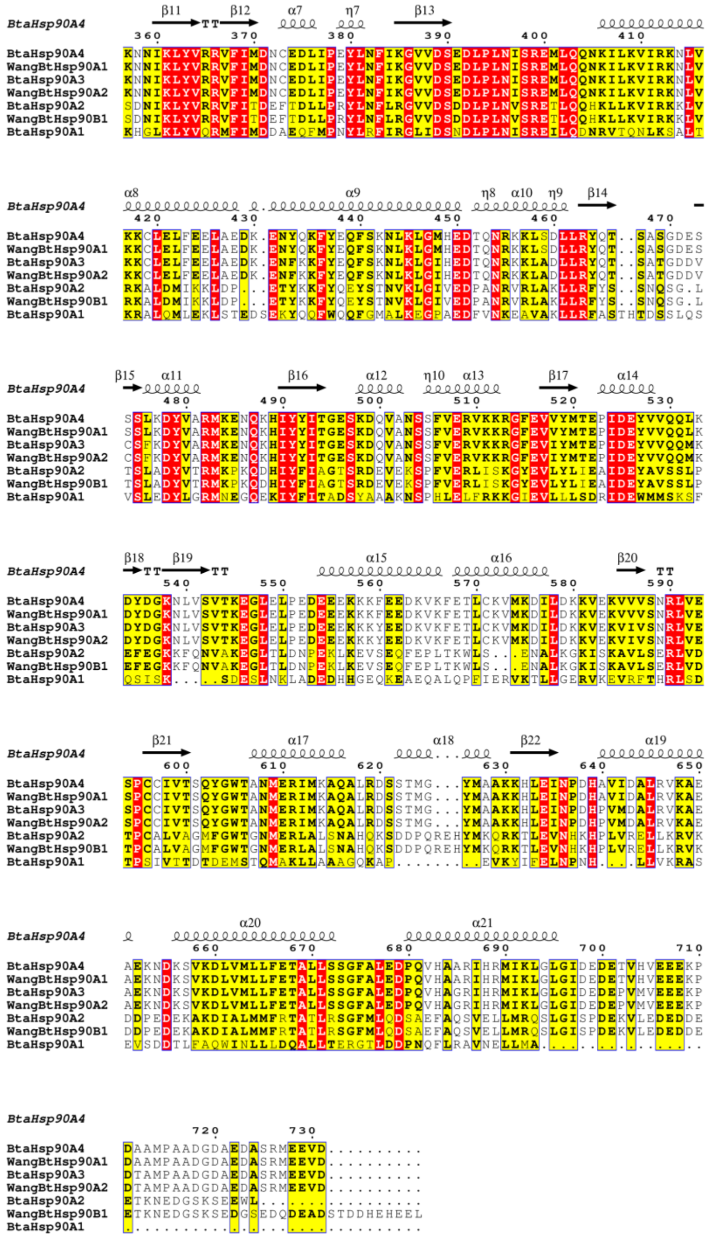

Supplementary Figure S9. The secondary structures of *Bemisia tabaci* HSP90s. α-helices and β-sheets were represented by yellow boxes and blue arrows shown above the sequence, respectively. The conserved domain of HSP90s were marked with a line under the sequences.

Supplementary data sheet 1

1. Sequence logos for the conserved motifs of sHSPs in *Bemisia tabaci*

BtasHsp Motif

Motif 1

E-value 5.2e-138

Width 39

Sites 9

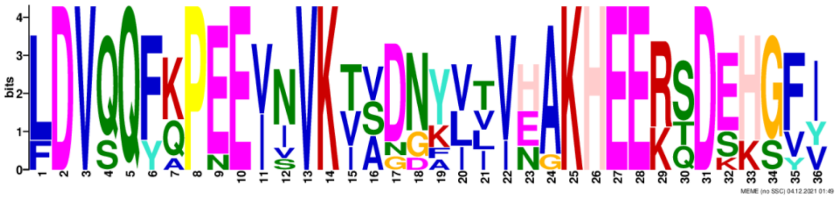

Motif 2

E-value 2.6e-133

Width 39

Sites 9

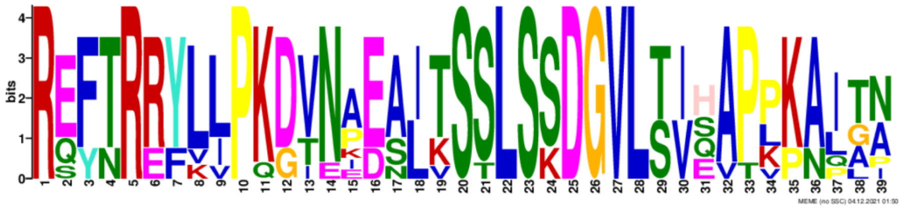

Motif 3

E-value 9.8e-077

Width 40

Sites 7

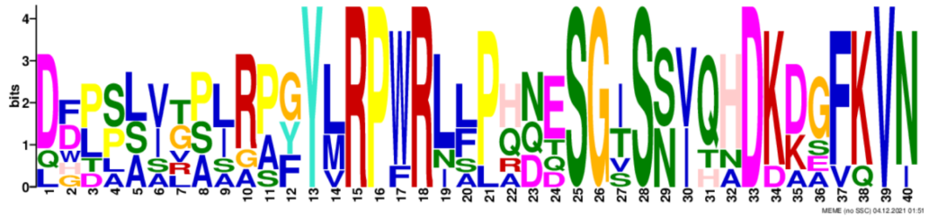

Motif 4

E-value 1.5e-041

Width 19

Sites 9

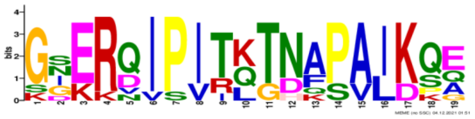

Motif 5

E-value 6.6e-035

Width 24

Sites 9

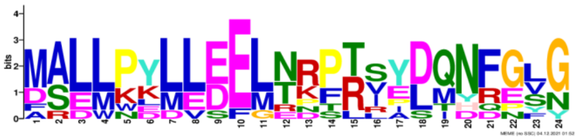

Motif 6

E-value 3.1e-010

Width 40

Sites 2

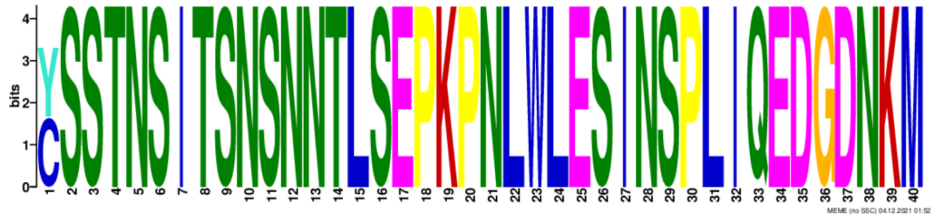

Motif 7

E-value 7.5e+000

Width 10

Sites 2

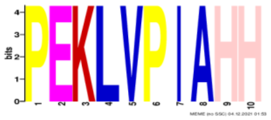

Motif 8

E-value 1.0e+002

Width 10

Sites 2

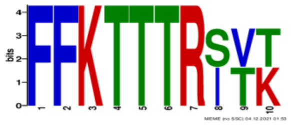

Motif 9

E-value 6.2e+003

Width 10

Sites 2

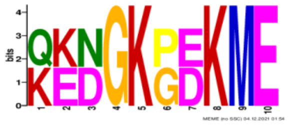

Motif 10

E-value 5.7e+004

Width 14

Sites 2

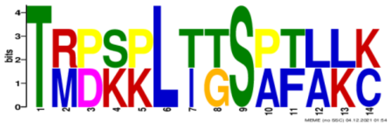

Motif 11

E-value 7.8e+005

Width 10

Sites 2

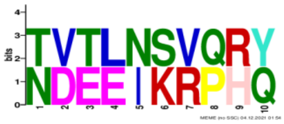

Motif 12

E-value 8.2e+005

Width 10

Sites 2

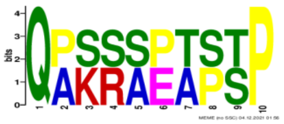

2. Sequence logos for the conserved motifs of HSP40s in *Bemisia tabaci*

BtaHsp40 Motif

Motif 1

E-value 1.7e-031

Width 34

Sites 4

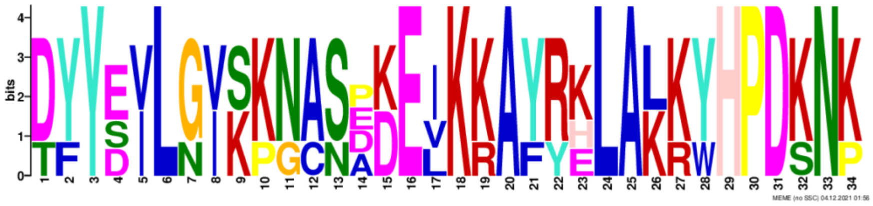

Motif 2

E-value 2.5e-019

Width 30

Sites 4

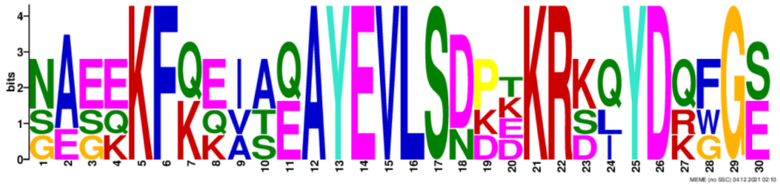

Motif 3

E-value 4.3e-002

Width 34

Sites 3

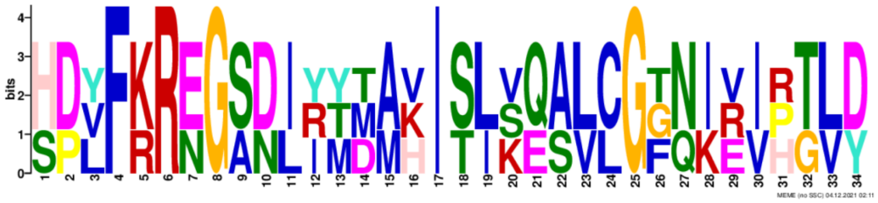

Motif 4

E-value 3.7e+001

Width 30

Sites 2

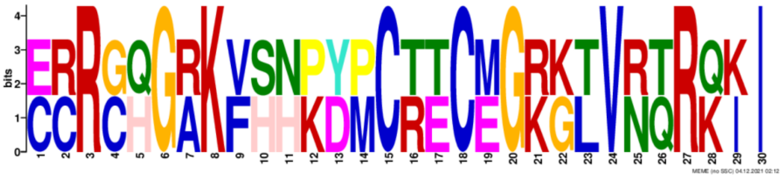

Motif 5

E-value 6.3e+001

Width 48

Sites 4

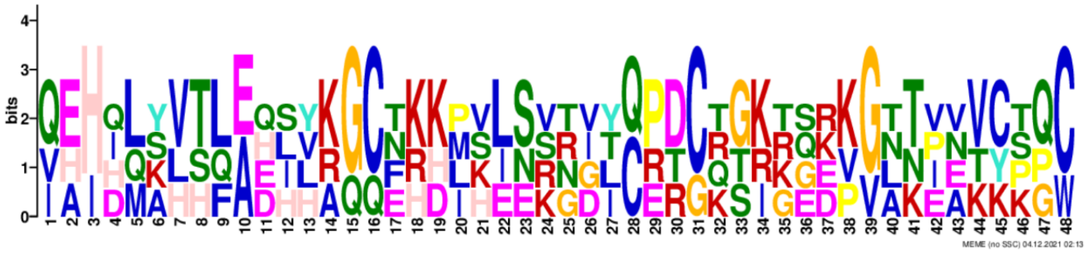

Motif 6

E-value 1.3e+002

Width 47

Sites 2

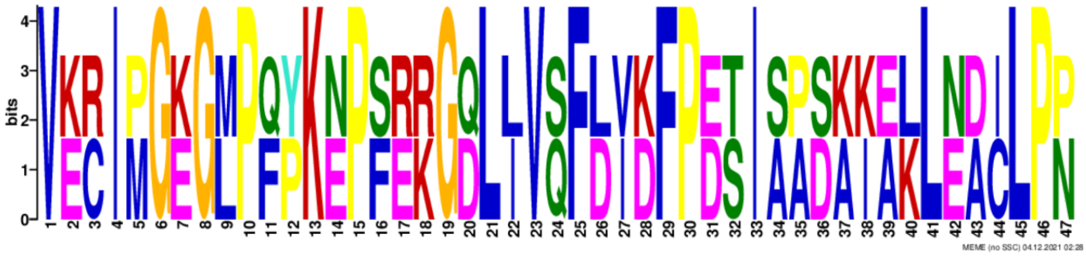

Motif 7

E-value 1.5e+003

Width 30

Sites 2

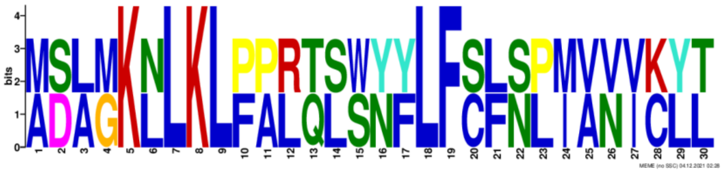

Motif 8

E-value 2.7e+004

Width 31

Sites 2

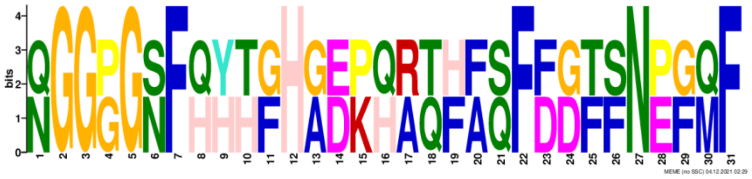

Motif 9

E-value 1.1e+004

Width 36

Sites 2

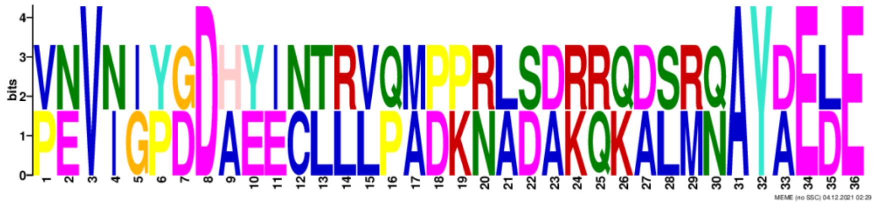

Motif 10

E-value 1.2e+004

Width 30

Sites 2

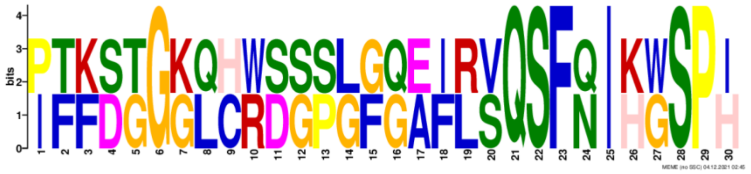

Motif 11

E-value 1.9e+004

Width 30

Sites 2

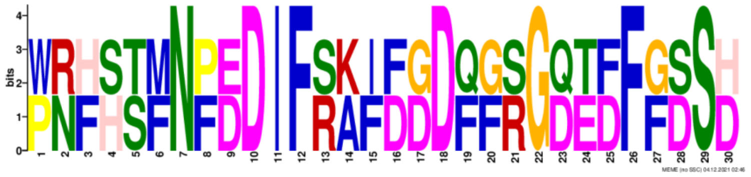

Motif 12

E-value 1.0e+005

Width 30

Sites 2

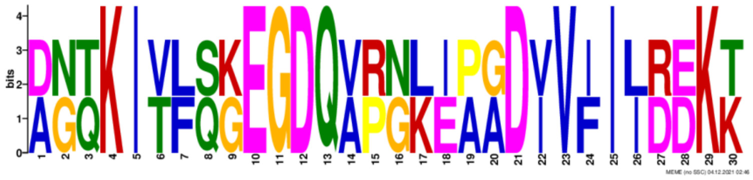

Motif 13

E-value 4.0e+005

Width 30

Sites 2

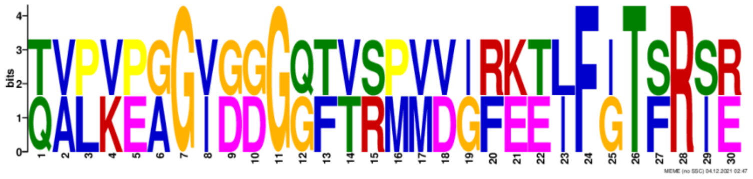

Motif 14

E-value 9.3e+005

Width 30

Sites 2

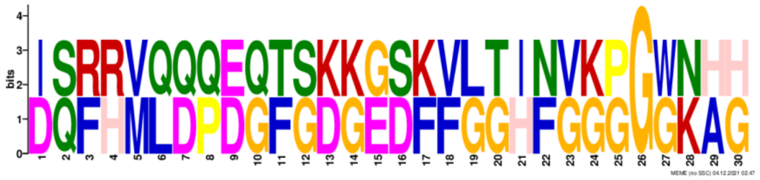

3. Sequence logos for the conserved motifs of HSP60s in *Bemisia tabaci*

BtaHsp60 Motif

Motif 1

E-value 4.2e-008

Width 70

Sites 2

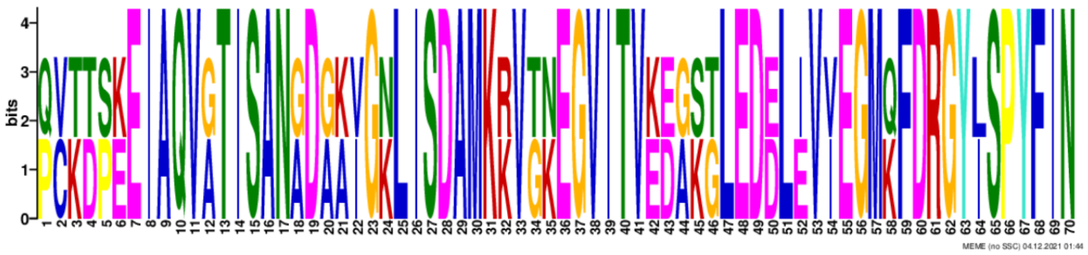

Motif 2

E-value 2.0e-006

Width 68

Sites 2

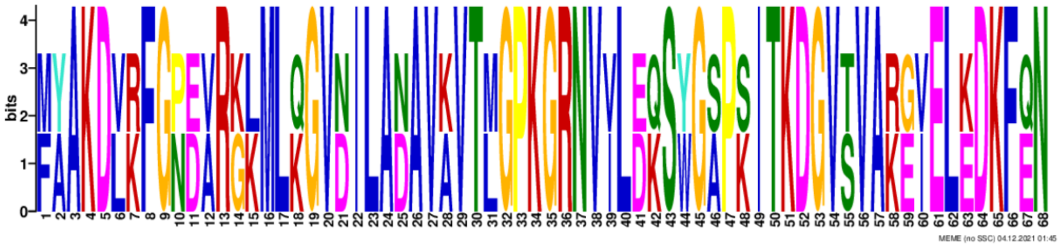

Motif 3

E-value 1.6e-003

Width 56

Sites 2

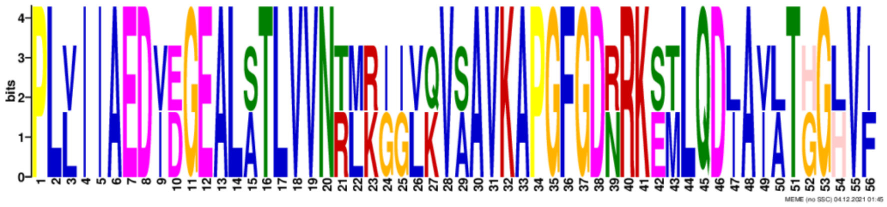

Motif 4

E-value 3.5e-003

Width 59

Sites 2

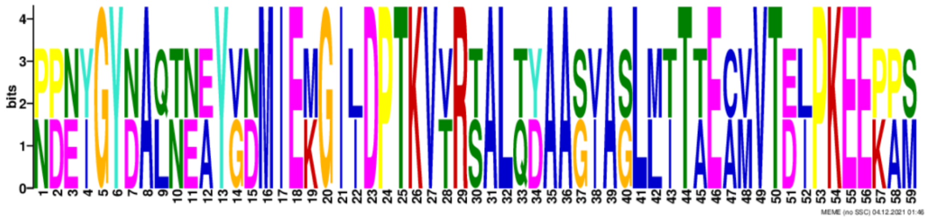

Motif 5

E-value 4.2e-002

Width 70

Sites 2

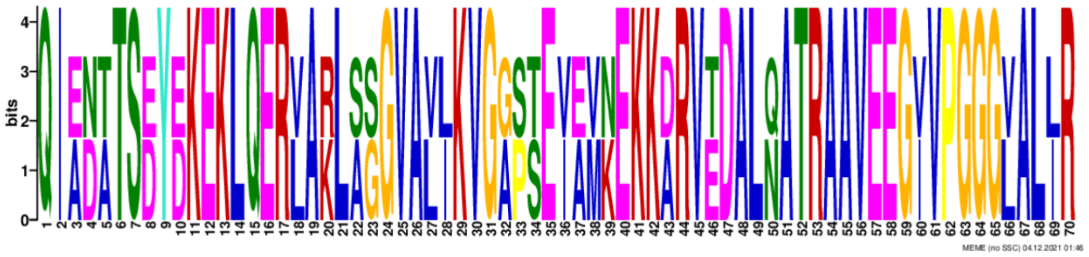

Motif 6

E-value 2.0e+001

Width 31

Sites 2

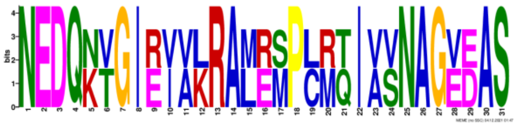

Motif 7

E-value 1.5e+002

Width 32

Sites 2

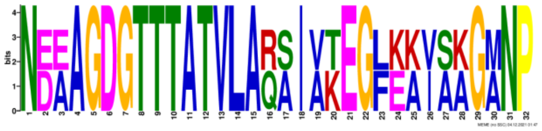

Motif 8

E-value 4.6e+002

Width 30

Sites 2

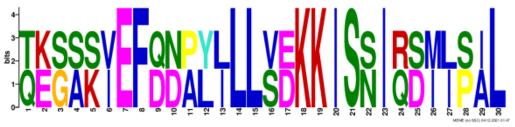

Motif 9

E-value 3.3e+003

Width 36

Sites 2

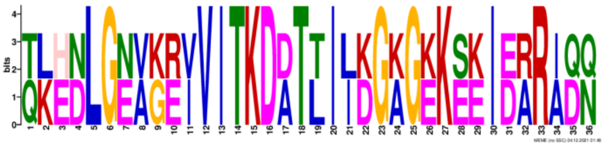

4. Sequence logos for the conserved motifs of HSP70s in *Bemisia tabaci*

BtaHsp70 Motif

Motif 1

E-value 7.3e-179

Width 70

Sites 7

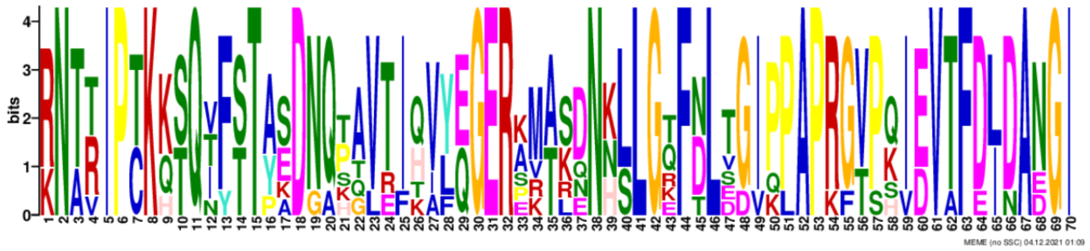

Motif 2

E-value 5.0e-147

Width 43

Sites 9

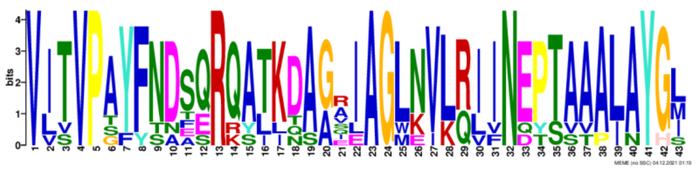

Motif 3

E-value 1.8e-149

Width 70

Sites 10

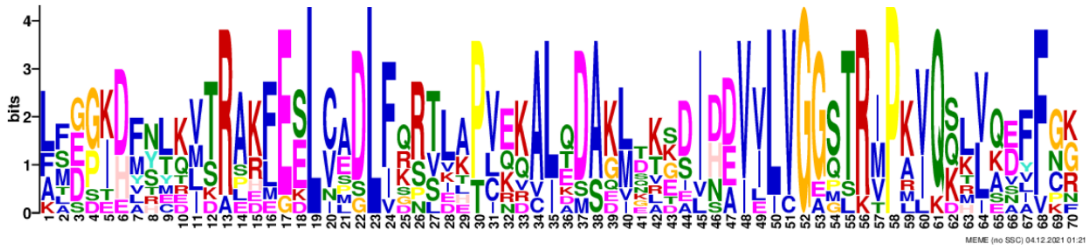

Motif 4

E-value 7.4e-122

Width 54

Sites 9

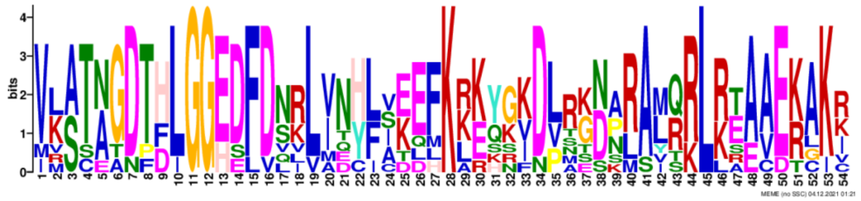

Motif 5

E-value 9.3e-088

Width 42

Sites 8

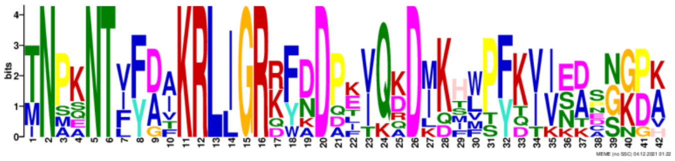

Motif 6

E-value 1.2e-064

Width 41

Sites 10

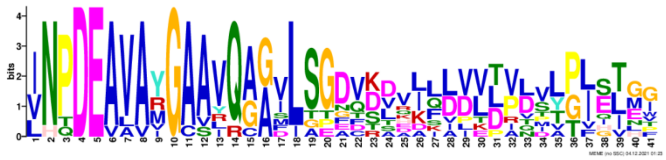

Motif 7

E-value 4.4e-049

Width 30

Sites 8

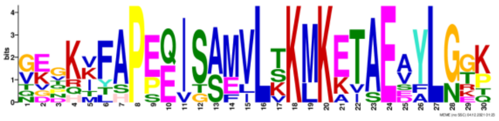

Motif 8

E-value 1.5e-046

Width 41

Sites 8

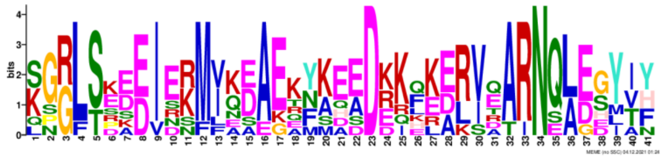

Motif 9

E-value 3.8e-046

Width 39

Sites 6

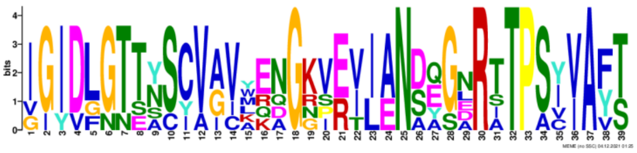

Motif 10

E-value 4.4e-033

Width 30

Sites 6

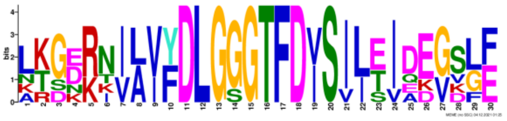

Motif 11

E-value 1.4e-012

Width 55

Sites 5

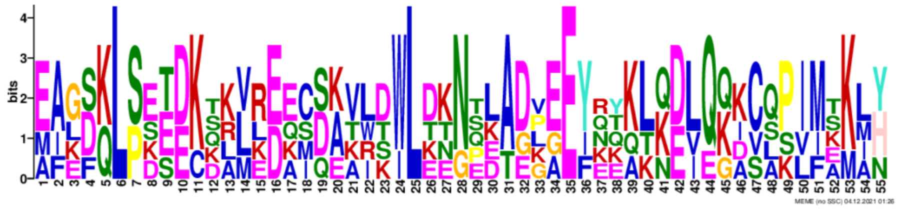

Motif 12

E-value 8.9e+001

Width 30

Sites 2

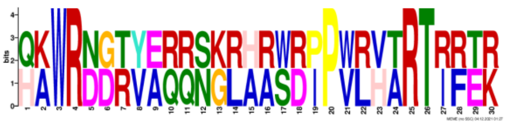

Motif 13

E-value 1.3e+003

Width 43

Sites 2

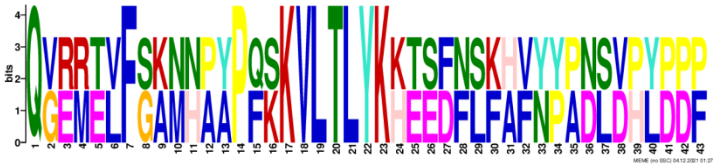

Motif 14

E-value 1.5e+004

Width 30

Sites 2

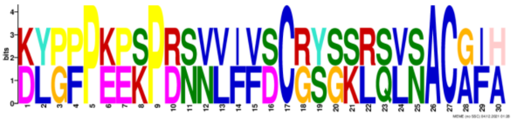

Motif 15

E-value 4.4e+004

Width 30

Sites 2

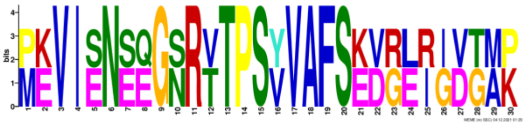

Motif 16

E-value 4.6e+004

Width 30

Sites 2

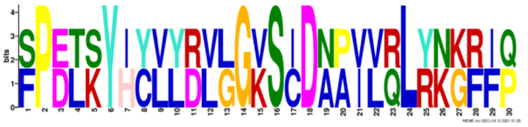

Motif 17

E-value 4.9e+004

Width 38

Sites 2

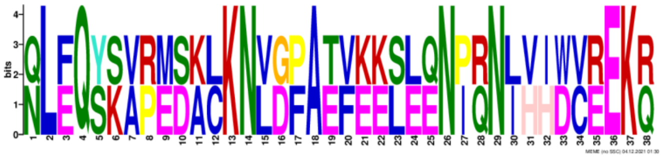

Motif 18

E-value 2.8e+004

Width 39

Sites 2

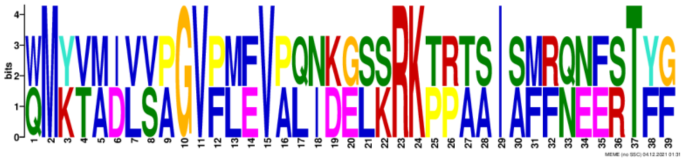

Motif 19

E-value 4.1e+004

Width 30

Sites 2

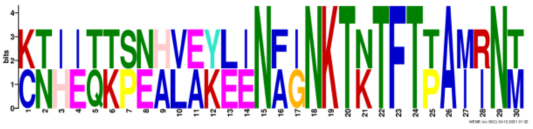

Motif 20

E-value 1.2e+005

Width 34

Sites 2

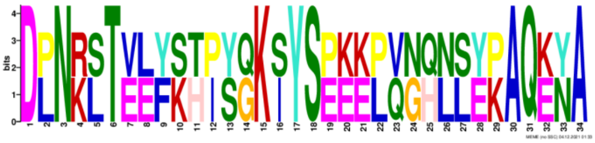

5. Sequence logos for the conserved motifs of HSP90s in *Bemisia tabaci*

BtaHsp90 Motif

Motif 1

E-value 3.2e-064

Width 70

Sites 4

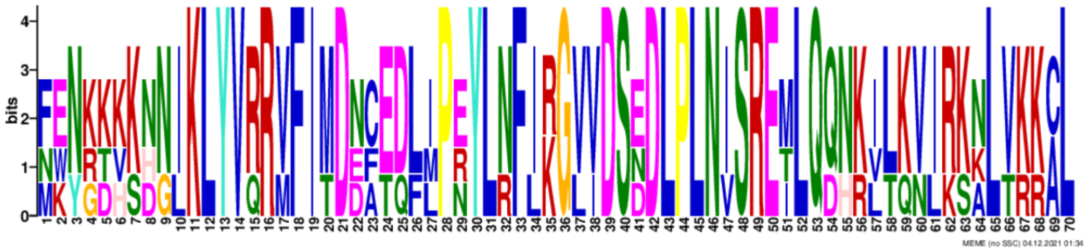

Motif 2

E-value 1.4e-059

Width 68

Sites 4

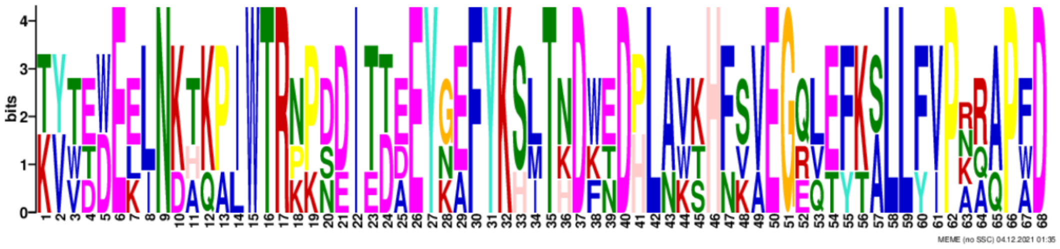

Motif 3

E-value 4.1e-049

Width 61

Sites 4

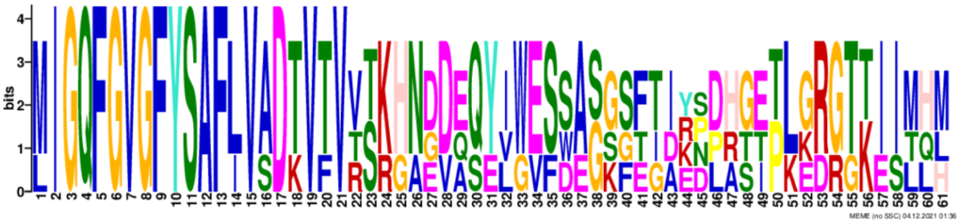

Motif 4

E-value 1.6e-045

Width 70

Sites 4

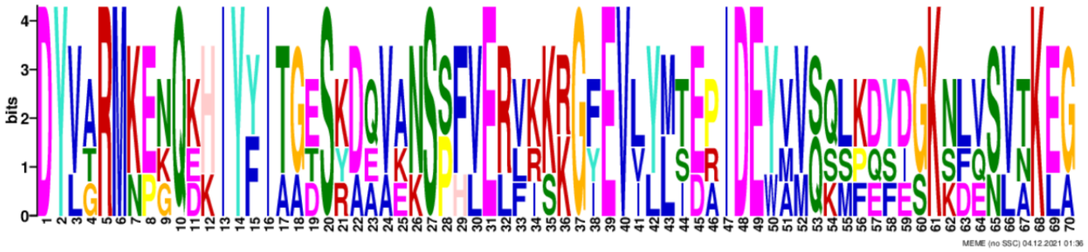

Motif 5

E-value 2.6e-029

Width 55

Sites 3

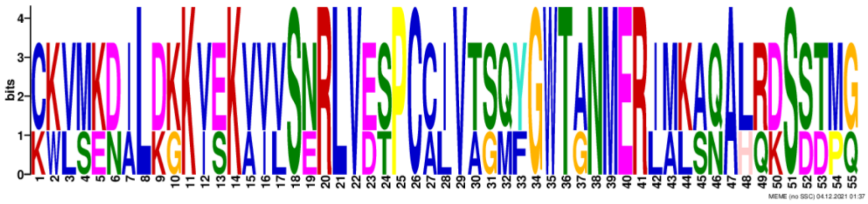

Motif 6

E-value 3.8e-027

Width 31

Sites 4

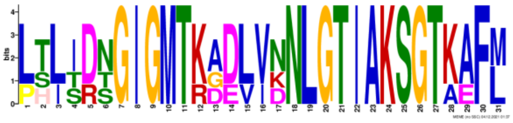

Motif 7

E-value 4.6e-023

Width 41

Sites 4

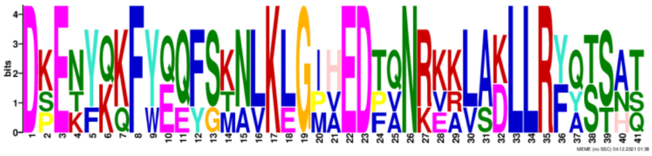

Motif 8

E-value 1.1e-015

Width 59

Sites 2

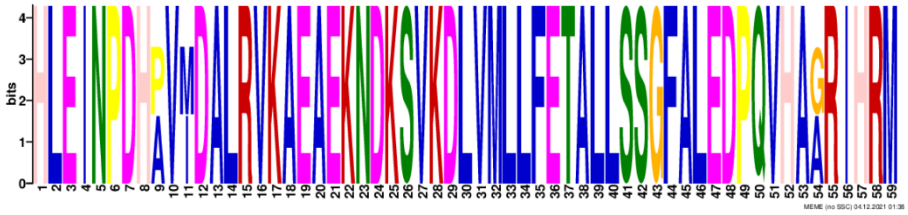

Motif 9

E-value 1.8e-018

Width 51

Sites 3

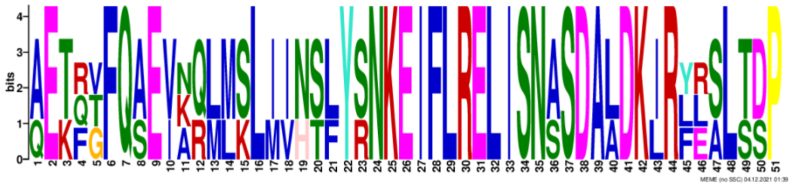

Motif 10

E-value 2.4e-008

Width 30

Sites 4

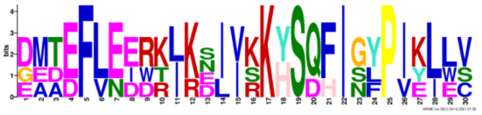

Motif 11

E-value 3.6e-003

Width 30

Sites 2

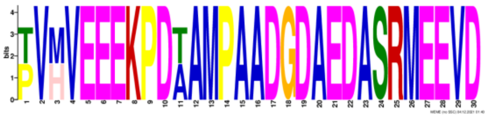

Motif 12

E-value 2.8e+004

Width 34

Sites 2

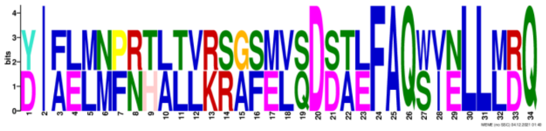

Motif 13

E-value 3.4e+005

Width 30

Sites 2

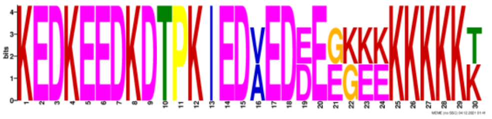

Motif 14

E-value 1.9e+005

Width 30

Sites 2

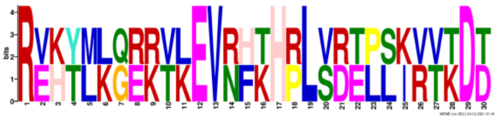

Motif 15

E-value 3.1e+006

Width 30

Sites 2

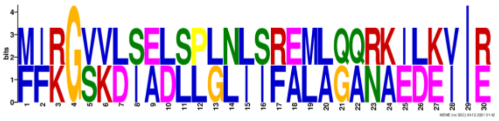

Motif 16

E-value 3.1e+006

Width 30

Sites 2

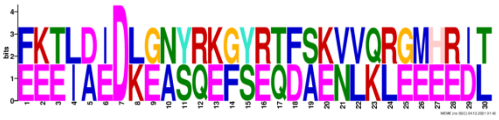

Motif 17

E-value 3.1e+006

Width 30

Sites 2

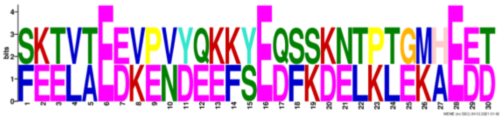

Motif 18

E-value 3.1e+006

Width 30

Sites 2

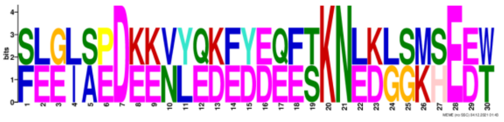

6. Sequence logos for the conserved motifs of HSP100s in *Bemisia tabaci*

BtaHsp100 Motif

Motif 1

E-value 1.2e-014

Width 57

Sites 2

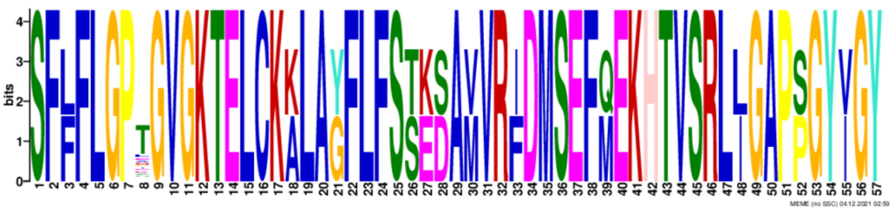

Motif 2

E-value 1.7e-006

Width 57

Sites 2

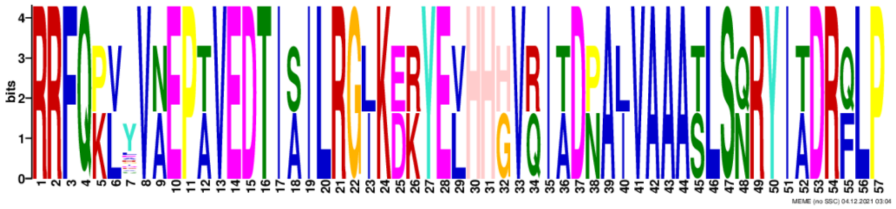

Motif 3

E-value 1.3e-005

Width 66

Sites 2

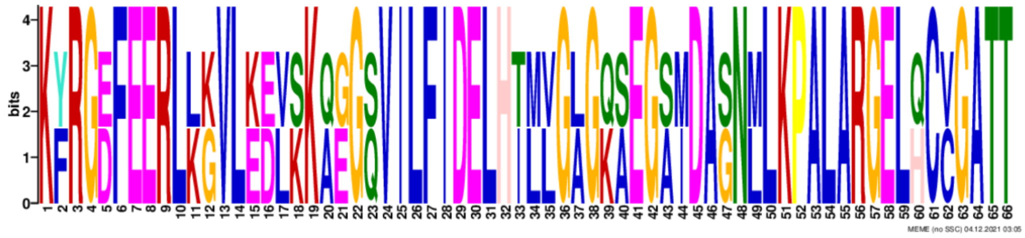

Motif 4

E-value 3.4e-005

Width 64

Sites 2

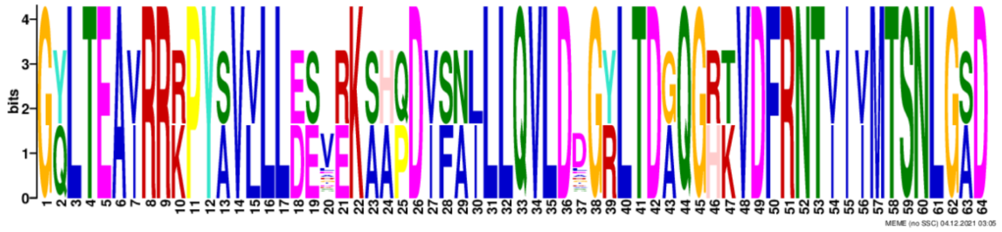

Motif 5

E-value 5.4e-002

Width 68

Sites 2

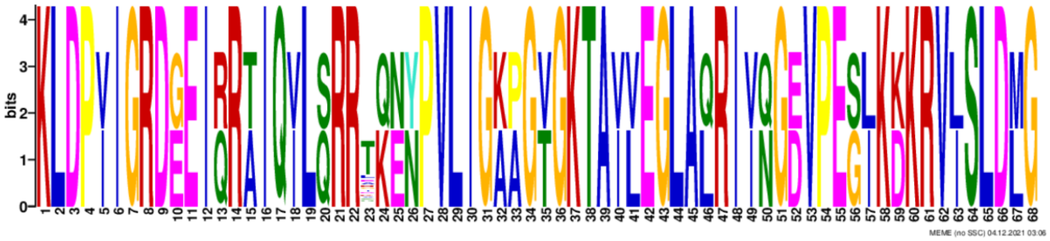

M

otif 6

E-value 9.4e-002

Width 70

Sites 2

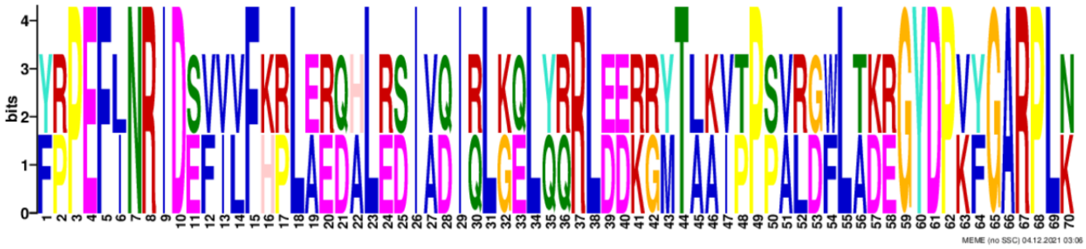

Motif 7

E-value 2.4e-001

Width 56

Sites 2

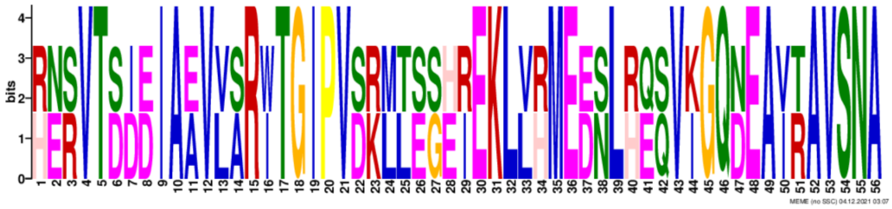

Motif 8

E-value 4.5e+001

Width 53

Sites 2

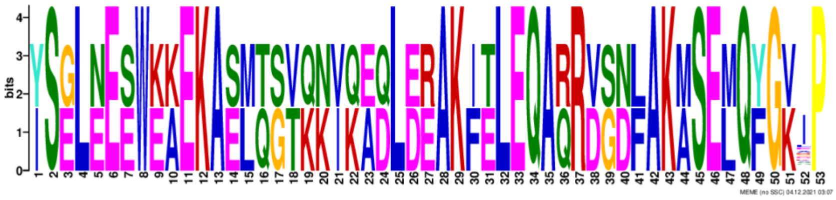

Motif 9

E-value 4.6e+003

Width 45

Sites 2

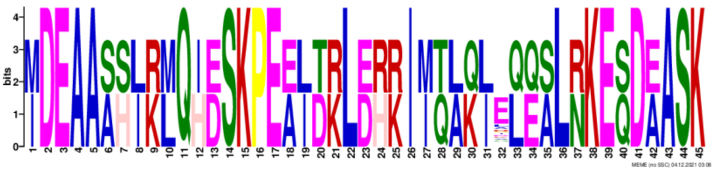

Motif 10

E-value 3.3e+004

Width 30

Sites 2

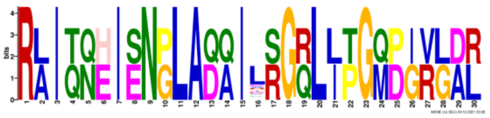

Motif 11

E-value 1.8e+004

Width 30

Sites 2

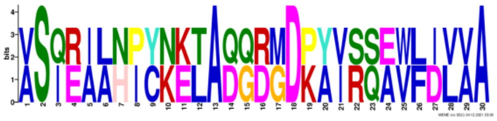

Motif 12

E-value 5.6e+005

Width 30

Sites 2

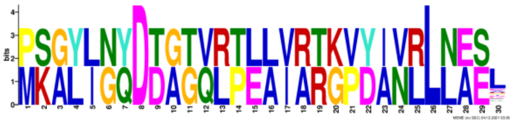

Motif 13

E-value 5.6e+005

Width 30

Sites 2

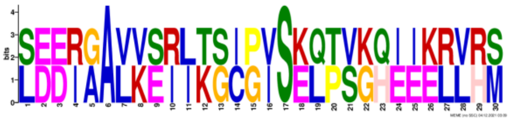

Motif 14

E-value 5.6e+005

Width 30

Sites 2

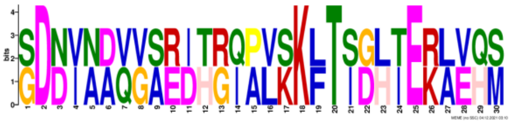

Supplementary data sheet 2

Information of *Hsp* sequences in the six surveyed species

Bemisia tabaci

>BtaHsp70-1

MVKAPAIGIDLGTTYSCVGVWQQGKVEIIANDQGNRTTPSYVAFSDTERL
IGDAAKNQVAMNPQNTIFDAKRLIGRRYDDPKIQDDMKHWPFKVINDC
GKPKLQVEFKGETKTFAPEEVSSMVLTKMKETAEAFLGGQVKDAVITVP
AYFNDSQRQATKDAGAIAGLNVLRIINEPTAAALAYGLDKNLKGERNVL
IFDLGGGTFDVSILTIDEGSLFEVRATAGDTHLGGEDFDNRLVNHLAEEFK
RKYRKDLRGNNRALRRLRTAAERAKRTLSSSTEASIEIDALMDGIDYYTK
VSRARFEELCSDLFRSTLHPVEKALADAKMDKGSIHDVVLVGGSTRIPKIQ
SLLQNFFCGKTLNLSINPDEAVAYGAAVQAAILSGDTSSAIQDVLLVDVA
PLSLGIETAGGVMTKIVERNARIPCKQSQTFTTYSDNQPAVTIQVYEGERA
MTKDNNLLGTFDLTGIPPAPRGVPKIDVTFDLDANGILNVSAKENSTGKS
KNIVIKNDKGRLSREEIDRMVNEAEKYKEEDERQRAKIAARNQLESYVFN
VKQAVDEAGDKLPESDKQLVRDECQAALSWLDNNTLADVEEFNYKLQE
VQKKCSPIMSKMHGAGQGGMHAWRNGRYARRNGRHAWRIPWRHAR
TRRTRPNC

>BtaHsp70-2

MNPKNTVFDAKRLIGRRFDDPKIQDDIKHWPFKVISDSGKPKIQVEFKGE
QKIFAPEEISSMVLTKMKEVAEVYLGGKVSEAVITVPAYFNDSQRQATKD
AGAIAGMNVLRIINEPTAAALAYGLDKNLKGERNVLIFDLGGGTFDVSIL

SIDEGSLFEVKSTAGDTHLGGEDFDNRLVTHFSEEFKRKYKKDLRGNARA

LRRLRTACERAKRTLSSSTEASLEIDALHEGELCMDLFRNTLAPVERALTD

AKMDKGSIHDVVLVGGSTRIPKVQKMLQDFFCGKSLNLSINPDEAVAYG

AAVQAAILSGDQSAEIQDVLLVDVTPLSLGIETAGGVMTKIVERNARIPC

KQTQTFTTYSDNQPAVTIQVFEGERSMTRDNNLLGTFNLSGIPPAPRGVP

KIEVTFDLDANGILNVSAKDNSSGKMERITIKNDKGRLSKEDIEKMLADA

EKYKHEDEKQKERVTARNQLEGYTFNVKQAVEEAGSKLSETDKKAVLD

KCSAVLTWLDTNSLAEKEEYEEKLKDLQKDCAPVMTKLHQQGGKGPTV

EEVD

>BtaHsp70-3

MEVISNEQGNRVTPSYVAFSEVRLIGDTAKMLAPINPKNTIFDVKRLIGRK

FDDPLVQADMKHWPFTVINDAGNPKVSVEYQGVDKVFAPEEISSMVLSK

LKETAEVYLNRKVTDAVITVPAYFNDSQRQATKDAGVIAGLNVLQIINEP

TSAAIAYGLDQNLKSKRNILVFDLGSGTFDVSVLTVDEGSLFEVKSTAGDT

HLGGEDFDSLLVNYLIEEFKLKHSKDLASNPRALSRLRAAAERAKCTLSS

APTANIMIDSLFDGIDFYTQVTRPKFEDLCADLFQRTLTTVEQALTDAKIE

REAIDDVVLVGGSTRIPKIQSLLQDFFGGKTLNQSIHPDEAVAYGAAVQA

ASLSGNDDLKIKDIVLRDVTPLSLGVQMTNDVFSVVIKRNTRIPCKKTQN

YTTPKDNAKGLRFTIYEGERAVASENHLLGEFNLEGIKLAPKFVPSVDVTF

DLNAEGILLVTAEEKGTDKSENITITNEKGRLTDKDIRRMIQEAKRFKEED

DKHLARSRARNQLGDYTYKVTQELKAAESKLSKTDKSRLKEESDKTWD

WLKKNPKAGLGEYKQKLQDIQGKCQSIMKKINKDDALLD

>BtaHsc70-1

MRLLFLSGAFCLLACVAFAKEEKKDKEDFGTVVGIDLGTTYSCVGVYKN

GRVEIIANDQGNRITPSYVAFTAEGERLIGDAAKNQLTTNPENTVFDAKR

LIGRDWNDPTVQQDIKFFPFKVKEKNSKPHIEVSTSQGVKIFAPEEISAMV

LSKMKETAEAYLGKPVVSTNGDTHLGGEDFDQRVMDHFIKLHKKKKGK

DVRKDNRAVQKLRREVEKAKRALSSAHQVRIEIESFFDGEDFSETLTRAKF

EELNLDLFQSTLKPVQKVLEDADMNKKDVDE

IVLVGGSTRIPKVQQLVKEYFNFKEPSRGINPDEAVAYGAAVQAGVLSGE

QDTDAIVLLDVNPLTLGIETVGGVMTKLIPRNTVIPTKKSQIFSTASDNQH

TVTIQVYEGERPMTKDNHLLGKFDLTGVPPAPRGVPHIEVTFEIDANGIL

QVSAEDKGTGNREKIVITNDQNRLTPDDIERMIKEAEKFADDDKKLKERV

EARNELESYAYSLKNQLADKEKLGSKVDGMILFQLSDEEKTKMEEAIDEK

IKWLEENQDTDGAEYQKQKKDLEQIVQPIIAKLYAGQGPPPPAGGDSDD

LKEEL

>BtaHsc70-2

MEGKTPKVIENSEGSRTTPSVVAFSKDGERIVGMPAKRQAVTNSANTFYA

TKRLIGRRFDDAEVKKDMKTLSYKIVKASNGDAWVQATDGKMYSPSQIG

AFVLVKMKETAESYLGTPVKNAVITVPAYFNDSQRQATKDAGQIAGLNV

LRVINEPTAAALAYGMDKTDDKIIAVYDLGGGTFDISILEIQKGVFEVKST

NGDTFLGGEDFDNVLVNHLVTEFKKEQGIDVTKDAMAMQRLKEAAEK

AKIELSSSLQTDINLPYLTMDASGPKHMNLKLTRSKFESLVADLIKRTVAP

CQKAIQDAEVGKSDISEVLLVGGMTRMPKVQQTVQEIFGRQPSRAVNPD

EAVAVGASVQGGVLAGDVTDVLLLDVTPLSLGIETLGGVFTRLISRNTTIP

TKKSQVFSTAADGQTQVEIKVYQGEREMASNNKLLGQFTLVGIPPAPRG

VPQIEVTFDIDANGIVHVSARDKGTGKEQQIVIRSSGGLSKDEIENMVKSA

EEYAQSDKIKKERVEAINQAEGIIHDTESKMTEFKDQLPSEECDKLREQMS

KVRDILTKGEEADPEEIRTATNELQQASLKLFEMAYKKMAADRDGSSSSS

SSEEQSSEEKGEEKEKKENKQ

>BtaHsp70-4

MTKIIGIDLGTTNSCVAIMENGNPRVLENSEGDRTTPSIIAYTQDGETLVG

QPAKRQAVTNAKNTFFAIKRLLGRKFADQETQRDKDIMPFDIIEADNGD

AWLSVKGQKTAPPQISAEVLKKMKKTAEAYLGGTVTEAVVTVPAYFND

AQRQATKDAGRIAGLEIKRIINEPTAAAIAYGLDKATGNRTIAVYDLGGG

TFDISIIEIEEVKGEKTFEVLATNGDTHLGGEDFDNRLINYLADEFKKEQGF

DLRKDPLAMQRLKEAAEKAKVELSSAQQTDVNLPYITADAAGPKHMN

MRVTRAKLESLVEDLVSRSIEPLKVALQDAGLSVSDIDDVILVGGQTRMP

MVQKKVADFFGKEPRKDVNPDEAVAIGAAVQGGVLSGDVKDVLLLDVT

PLSLGIETMGGVMTSLISKNTTIPTKHSQVFSTAEDNQSAVTIHVLQGERK

RASDNKSLGQFNLDGIQPAPRGTSQIEVTFDIDADGILNVSAKDKNTGRE

QKITIKASSGLSEAEIEKMVKDAEANSEADRKFEDLIQARNQADHLINSTT

KKLKEAGDKVSPEEKTSIEQALQALETAIKGEDKTDIESKANALTMVSAK

LEEASQQNSSSNNAEKNDSSDVKADAVDAEFEEVKDKK

>BtaHsp70-5

MIRIIGIDLGTTNSCVAVLENGKSRVIENSEGARTTPSIIAYTDDNEILIGQT

AKRQAVTNPSNTLYAIKRLIGRKFNDDIVQKDIKMVPYKIVSAENGDAW

VQVKNRKLAPPQISAEILKKMKKTAEDYLGEKITEAVITVPAYFNDSQRQ

ATKDAGRIAGLNVKRIINEPTAAALAYGMDKNRGDKKIAVYDLGGGTF

DISIIEIADVDGETQFEVMATNGDTFLGGEDFDLKLINYIVKQFKLESGVDL

SGDSLAMQRLKEAAEKGKIELSSSQQTDINLPYITADKTGPKHLTLKITRA

KLESLVEDLIKRSLLPCKKAIDDAKLTTKEIDDIILVGGQTRMPLVQKKVA

EFFGKEARKDVNPDEAVAMGAAIQGGVLGGDVKDVLLLDVTPLTLGIET

MGGIMTPLIEKNTTIPTKKTQIFSTAEDNQTAVTIHALQGERKKALQNKSL

GRFDLTDIPPAPRGTPQIEVAFDLDANGILNITAKDKATGKEQSIIIKSSGG

LSEEEINKMIQDAETNKEEDKKFEELVQTRNQADGMIHTIRKTLKEIKNE

DEKNKLESLINNLEQTLKSDDIKTIKEDLNKLTEEYSTIYQKIYSEKKPQNH

NSEPAQKNAEKSSSKKEGDVVDAEYEEVNNKTK

>BtaHsp70-6

MSRFTVFGIYVGNTSASIAICKEDGKVEVLANAAGERSTPAVVAVTEKEQI

VGNAAKSSMITQTNAGTIITNNKLLMDLSLDESDLESIITKNPCTIIQKSNH

VAYEINFGNKTKTFTPAMINTSIYSLLYNIAKSAIHGECDEICCVLVVPTYY

SAESRQYIKQSAIEAGWKVLQIVNQPSVAPLAYHSFKEASPETKYICVYRV

GGVSCDAAILRLRNGFIQILASETNFDIGGHELVSKLIQCFCEEMKRKYKV

DPNESKRSIRKLRSAAETCIKVLSTLSSASVSVDSLFEGIDFNYQMSRARFES

LIPSLFGPLDAPINDALQSAQLQATDINEVILCGEPLKTPRLQSHVKSLFPN

AEVLSSINPDEVLARGAARQAGFITEFCDLHKSIPEPVTDVTFITEPITVQIQ

SSNNEEIVFEKFSSLPISKTVHLKDNKSEIIVTAAQSCSAESKLNGKINAEQ

AIEIHLLNKEMEELQMYTMDLVAGVFLFVPQIDGLSRKPRTSIAMFQNFST

YFIFS

>BtaHsp105/110

MDWRKYLLAILLSVAFVAVHVSGLAVMSIDLGSEWMKVAIVSPGVPMEV

ALNKESKRKTPAAISFRNEERTFGDDALNVAVRFPDLSYHYLLDLLGKSID

NPVVQLYKKRFPFYNLEADPNRSTVLFKHPSGKSYSPEELVGQLLYKAQE

YAQNSANQVVTEVVLTVPGFFNDFERRSLITAAELAGLKVLQLINDYSAV

ALNYGIFRRKEFADSAQYVMFFDMGASSTTATIVQYQTVKVKDRGIVETL

PQASVIGVGFDRTLGGLEMTLRLRDYLAQK

FNEMKKTDIDVTQNPRAMAKLFKEAGRVKTVLSANADHFAQIEGLLEGI

DFRLKVTREEFEKLCADLFDRVKAPVEKALKMSGLTTDILNAVVLVGAG

TRVPAVQDRLSAAVKRDLARNLNTDEAAVMGAVYRAADLSTGFKVQK

FLVKDAVIYPIQVHFDRDGDASSKQVRRTLFGAMNPYPQKKVLTLYKHT

EDFSFHVNYADLDHLDPFAVEALGSLNLTKVNLKGVPEALAKFSTEGHE

LKGIKAHFHMDDSGILSLPNVELVAEKTISAEEESPLSKLGSTISKLFSGSEN

SKDNPDVRPVTEEAEENEKENKTEQAKEKPESKEPPPADGAKKNDTEKE

KKDAKDETPKKPKIVTLKEPIKAEIEDLGIPVLSGDAYKATSELIEAYNKAE

LEKAHQEKALNLLESAVISYKQKLQDDEFVSFANTDEVDKIKARSNELSE

WLDEEGYSADTKTLTKKLDEFDTLVKPVLEKKKNHETTPEALEKLENAIN

KTNTFTTAIRNMTATAEGDLPPFYSETEMDGIDKLITDIQKWRDDTVEQQ

SKLARSDPPVLVT

RTIFEKIQLIETETRILINKVKLWDQAMKAKSAQDATKNDTKKDKAGKKR

SKSKQTEGEQAKSEKGTADKEAGESVPETEPQVETPAESEPEQPLQEQPEK

VAEPSTPEAESSPSTPDSDPPKHAEL

>BtaHsp70-7

MAAMSVIGIDFGNESCYVAVARAGGIETIANDYSLRATPSCVAFSEKTRIL

GVAAKNQLVTNMKNTVYGFKRLLGRQYKDPFIQKDLQSLTYQTVETPSG

GVGIKVNYLNEPHVFHPEQITAMLLTKLKAISEEALNTKINDCVISVPSYF

TNTERKALLNAASIAGLNVLRLFNETTATALAYGIYKQDLPPPEEKPRNV

VFVDCGYSSLQVSACAFHKGKLKMLACAADPDLGGHSFDVILAEHLSKD

LKARYNINPMTNARAYTRLLTEVDKLKKQMSANSTKLPLNIECFMEDKD

VHCDMKRADMEELCAGLFQRVEKTLRQCLEDSKLKLEDIHAVEIVGGSS

RVPAIKQLIEVVFNKPASTTLNQDEAVARGCALQCAMLSPAVRVRDFAV

TDIQMYPVHINWDGHDAQDQGEMEVFSKNHAAPFSKVLTLYKKESFNL

KAFYPPNSVPYPDPRIGEYCIKNIKPTAQGGTQKVKLKIRINIHGIVSVSSAS

LIEQIKGSAEPMDVEVNEEEHKEKEQPAQETTQEQQQPPEANANSQDAQ

PNGPAEDDDAEKKEKKRKTVKSVDLPIEEFLPGFSSSEVSSFFEAEGQMM

AADRQEKDRVDARNSLEEYVYELRGKLSEELSAYVVEKDRSSLVEQLDQ

MENWLYEEGEECVKQVYMDKLGSLRRKMEYEARPKALEELSVAIQLAFK

NLEQYKAREDKLKNLDPATVEKSEQNIRNLHHWCEEKRAILSATPLHQN

PPVSVAEILAEKEKAVSEIKLLSTMPKVAPEPAKQQPPPEQSQESTQDEKM

KTENGEQD

>BtaHsp67B2

MQANLFRRFLPALVSCSSIKRGTFLTLKQENLNFAYNTRVQLFQSSVPMS

ACKNVGFAEFKELLENPQNIVIDVREKQELVDDGRIEKTHNIPLGEVTQA

LQLEPSAFEAKYGFPKPSPDSNLIFSCRSGKRSLNACGIAEKQGFKNLWNY

SGGFLDWSEQTKGK

>BtaHsp60

MAAKDLKFGNDARKKMLKGVNILANAVKVTLGPKGRNVVLDKSYGAP

SITKDGVSVAREIELEDKFENMGAQMVKEVASKANDAAGDGTTTATVL

AQSIVTEGLKAVAAGMNPMDLKRGIDKAVEAAVEELKIISKPCKDSKEIA

QVGTISANADAKVGKLISDAMKRVTNEGVITVEEASGLEDDLIVVEGMQ

FDRGYLSPYFINKQESSSIEFDNPYILLVDKKISNIRDMLSILEVVAKEGKPL

LIIAEDVEGEALATLVVNTMRGIVKVSAVKAPG

FGDRRKEMLQDIAVLTHGHVISEETGDSLEKATKENLGNAKRVVITKDA

TTIIDGAGEKSKIEARIQNIKKQIENATSDYDKEKLQERVAKLSGGVAVIK

VGAPTEIAMKEKKARVEDALQATRAAVEEGVVPGGGVALIRVASKIANS

SLKGDNEDQNVGIRVALRAMESPLRQIVVNAGEEASVIANKIKENKGND

NYGYNAQTEAYGDMIEMGILDPTKVTRSALQYAASIAGLMITTECMVTD

LPKEEKASDMGAGGMGGIGGMGGMNGMM

>BtaHsp90A1

MQDQETRGFQSEVKQLLSLMIHSLYSNKEIFLRELISNASDAADKLRFRAL

SSPELYEGEAELRVRLSFDKDKRTLTLSDNGIGMTRDEVIDNLGTIAKSGT

KAFLDSLGSDQSKDSQLIGQFGVGFYSAFIVSDTVTVRTRGAGVASELGVF

WESKGEGDYNLATITKEDRGTEITLHLKEGEDEFLNDWRLRSIISKYSDHIS

LPVELECKNKDENQEGSETEKNNLPDKTQTVVTWEKINKAQALWTRPK

SDITDDEYKAFYKHITHDFTDPLNWSHNKVEGRQEYTSLLYIPAQAPWD

MWNRDHKHGLKLYVQRMFIMDDAEQFMPNYLRFIRGLIDSNDLPLNISR

EILQDNRVTQNLKSALTKRALQMLEKLSTEDSEKYQQFWQQFGMALKE

GPAEDFVNKEAVAKLLRFASTHTDSSLQSVSLEDYLGRMNEGQEKIYFIT

ADSYAAAKNSPHLELFRKKGIEVLLLSDRIDEWMMSKSFQSISKSDESLNK

LADEDHHGEQKEAEQALQPFIERVKTLLGERVKEVRFTHRLSDTPSIVTT

DTDEMSTQMAKLLAAAGQKAPEVKYIFELNPNHLLVKRASEVSDDTLFA

QWINLLLDQALLTERGTLDDPNQFLRAVNELLMA

>BtaHsp90A2

MFRGSKLIALSLGLLIFALAGARAEDEIIEEKTIDIDLGASREGSRTDAEVV

QREEERILIDGLNVAQLRELKEKAEKQTFQAEVNRMMKLIINSLYRNKEIF

LRELISNASDALDKIRLLSLTDPGVLDTNSELAIRIKADKDNKVLHITDTGI

GMTKGDLVKNLGTIAKSGTAEFLSQMNEKSEVKQDLNDMIGQFGVGFYS

AFLVADKVFVTTKHNEDQQYIWESDASSFTIAEDPRGTTLKRGTTISLQLK

DEAADFVEIDTLKNLIRKYSQFINFPIYIWSSKTVTEEVPVDEEKSEDSKDET

PTEKAEEDVEVEESKDEEPKTKKVEKTVWDWELLNDHKPIWTRKPNEIE

DAEYNEFYKSMTKDKNDPLAKTHFVAEGEVTFKSLLFVPNAQPADSFNK

YGTVSDNIKLYVRRVFITDEFTDLLPRYLNFLRGVVDSDDLPLNVSRETLQ

QHKLLKVIRKKLVRKALDMIKKLDPETYKKFYQEYSTNVKLGIVEDPANR

VRLAKLLRFYSSNQSGLTSLADYVTRMKPKQDHIYFIAGTSRDEVEKSPFV

ERLISKGYEVLYLIEAIDEYAVSSLPEFEGKKFQNVAKEGLTLDNPEKLKEV

SEQFEPLTKWLSENALKGKISKAVLSERLVDTPCALVAGMFGWTGNMER

LALSNAHQKSDDPQREHYMKQRKTLEVNHKHPLVRELLKRVKDDPEDE
KAKDIALMMFRTATLRSGFMLQDSAEFAQSVELLMRQSLGISPDEKVLED
EDDEETKNEDGSKSEEWL

>BtaHsp90A3

MTELSPSLIRNGIGMTKADLVNNLGTIAKSGTKAFMEALAAGADISMIGQ
FGVGFYSAFLVADTVTVVSKHNDDEQYVWESSAGGSFTIKSDHGEPLGR
GTKIIMHMKEDMTEFLEERKIKDIVKKHSQFIGYPIKLLVEKERDKELSEDE
EEEEVKKEDKEDKEEDKDTPKIEDVEDEEEGKEKKKKKKTIKEKYTEDEEL
NKTKPIWTRNPDDITTEEYGEFYKSLTNDWEDHLAVKHFSVEGQLEFKAL
LFVPRRAPFDLFENKKKKNNIKLYVRRVFIMDNCEDLIPEYLNFIKGVVDS
EDLPLNISREMLQQNKILKVIRKNLVKKCLELFEELAEDKENFKKFYEQFS
KNLKLGIHEDTQNRKKLADLLRYQTSATGDDVCSFKDYVARMKENQKH
IYYITGESKDQVANSSFVERVKKRGFEVIYMTEPIDEYVVQQMKDYDGKN
LVSVTKEGLELPEDEEEKKKYEEDKVKFETLCKVMKDILDKKVEKVIVSN
RLVESPCCIVTSQYGWTANMERIMKAQALRDSSTMGYMAAKKHLEINP
DHPVMDALRVKAEAEKNDKSVKDLVMLLFETALLSSGFALEDPQVHAG
RIHRMIKLGLGIDEDEPVMVEEEKPDTAMPAADGDAEDASRMEEVD

>BtaHsp90A4

MSSWCTLGLFRSRSEMPEDATMEQAETFVFQAEIAQLMSLIVNTFYSNKEI
FLRELISNSSDALDKIRYESLTDPSRLESGKELFIKIIPNKNDNTLTLIDSGIG
MTKADLVNNLGTIAKSGTKAFMEALAAGADISMIGQFGVGFYSAFLVAD
TVTVVSKHNDDEQYIWESSAGGSFTIRPDHSEPLGRGTKIIMHMKEDMTE

FLEERKIKEIVKKHSQFIGYPIKLLVEKERDKELSEDEEEEEEKKEDKEEDK

DTPKIEDAEDDEGKEKKKKKKTIKEKYTEDEELNKTKPIWTRNPDDITTEE

YGEFYKSLTNDWEDHLAVKHFSVEGQLEFKALLFVPKRAPFDLFENKKK

KNNIKLYVRRVFIMDNCEDLIPEYLNFIKGVVDSEDLPLNISREMLQQNKI

LKVIRKNLVKKCLELFEELAEDKENYQKFYEQFSKNLKLGMHEDTQNRK

KLSDLLRYQTSASGDESSSLKDYVARMKENQKHIYYITGESKDQVANSSF

VERVKKRGFEVVYMTEPIDEYVVQQLKDYDGKNLVSVTKEGLELPEDEEE

KKKFEEDKVKFETLCKVMKDILDKKVEKVVVSNRLVESPCCIVTSQYGWT

ANMERIMKAQALRDSSTMGYMAAKKHLEINPDHAVIDALRVKAEAEK

NDKSVKDLVMLLFETALLSSGFALEDPQVHAARIHRMIKLGLGIDEDETV

HVEEEKPDAAMPAADGDAEDASRMEEVD

>BtaHsp100

MKALLNQDTGTVRPLLVATGVDINRLNAELDKALSDLPVVEGIGGDVRL

SNASIRILNICNKLAQQRGDKYISSEWFILAALEERGALKEILKSCGISEQPV

KQEIERLRSGDNVNDQGAEDHRQALKKFTIDLTERAEQSKLDPVIGRDEE

IRRAIQVLQRRTKNNPVLIGAPGVGKTAIVEGLAQRIVNGEVPEGLKKKR

VLSLDMGALLAGAKYRGEFEERLKGVLKDLSKQGGSVILFIDELHTMVG

AGKAEGAMDAGNMLKPALARGELHCVGATTLDEYRQYIEKDPALERRF

QKVYVAEPTVEDTIAILRGLKERYELHHHVQITDPAIVAAASLSQRYIADR

QLPDKAIDLIDEAAASIRMQIDSKPEALDKLERRIIQLKLEQQALNKEQDE

ASKKRLKMLNDELEKKERQYSELEEEWKAEKASLTGTQNIKAQLEEAKIT

LEQARRVSDLAKMSEMQYGKIPALEKQLQAATEKEMSSMKLLRNRVTDI

EIAEVLARWTGIPVDRMLESEREKLLRMEENLHQQVIGQNEAVTAVSNA

LRRSRAGLSDPNRPIGSFLFLGPTGVGKTELCKALAYFLFSSKDAMVRIDM

SEFMEKHTVSRLLGAPPGYVGYEEGGYLTEAVRRRPYSVVLLDEVEKAHQ

DVFNILLQVLDDGRLTDGQGRTVDFRNTVIIMTSNLGSDLIQGRFSQADY

SDMKKSVMEVVQRYFRPEFINRIDEVVVFHPLEEQHLESIAQIQLKQLYQR

LEEKGYTAKITPSALGFLTKEGYDPVYGARPLKRAIQQEIENPLAQQILSG

QLIPGMPIVLDLNEGHILARQ

>BtaHsp23.8

MADGIKRDIPIRLGDFSVIDSEFSNIRERFDAEMKKMEDEMTKFRSELMNR

ESNFFKTTTRIVTKKTRDKKLIGSAFLLCCSSTNSITSNSNNTLSEPKPNLW

LESINSPLIQEDGDNKMLKLRFDVSQYQPEEIIVKTVDNKLLVHAKHEEKT

DSKSVYREYNREFLLPKGTNPESIKSSLSKDGVLTVEAPLPALGAPEKLVPI

AHH

>BtaHsp40-1

MDLMKNLKLPALQLWYFLFCLSLMVVVCLTEDDYYEVLNVKKNASEKEI

KKAFRHLAKRWHPDKNKEEGAEQKFQKIAQAYEVLSDPDKRKQYDQFG

SASFETGGQPRHHTFNFDDIFSAFDDDFGSGQTFFFSSHGNGGGGSFHHH

GHAEKHRQHFSFDDFFNEGMFDQFHMQDQEQFGDGGSFFGGHFGGGG

GNHHHHHHQAHHQAHHQAHHHAQQFHHPHESSGGYQRTCRTITQR

VGNTVATYKQCS

>BtaHsp40-2

MASAGKLLKLFPRTSSNYLFSFNPIANIKYLFVFQHQRSLFHTSSRLMKRD

YYEVLGISKNASAKEVKRAYYELAKKYHPDSNKSDPNASKKFQEATEAY

EVLSDDTKRKQYDQWGSTSEQMGGPGPGAGAGAGNFNSWNFSSMNPE

DIFRKIFGDQFRGDEDFGDSDSDFGVNTAQEIIMKLTFAQSVRGCNKDIVL

NVTDTCPRCQGKRSEPGTKPVKCTQCNGTGMEVVNSGPFMVRSTCRRC

HGAKVHNPYPCTECEGKGTVNQRKKITVPVPAGVDDGQTVRMVVGRK

ELFITFRIERSDYFRREGADIYTDAVISISQAVLGGNIRVRGVYEDQNILIAP

GTSSHTKVRLSGKGLKKVNVNGYGDHYINLRVQAPKRLSDKQKALMQA

YAELEDTTEGTVHGFVITKDGGKQCWDSSLFQEILVQSFQIKWSPIAKRKI

KLTF

>BtaHsp40-3

MGKDYYSILGISKGASDDEIKKAYRKLALKYHPDKNKAKSAEEKFKEVAE

AYEVLSDKKKRDIYDKFGEDGLKGNVGGGGGQGGPGNFQYTFHGDPQ

ATFAQFFGTSNPFQFFDLGGGGTRMFFGGDDDDDDPFFSTGGLHRSGPG

GGAFRSQSFNIHGSPHRDKDKQQDSPIEHDLYVSLEDILKGCTKKMKISRR

VLQPDGTSKKEDKVLTINVKPGWKAGTKITFQKEGDQVRNKIPADIVFIIR

DKTHPLFKREGSDIRYTAKITLKQALCGTNIEIPTLDNYKVPLQLTHKIIKP

TTVERIPGKGLPFPKEPSRRGDLLVSFDIKFPETISPSAKELLNDILPNS

>BtaHsp40-4

MVVETTFYDILGVKPNCNPDELKKAYRKLALKYHPDKNPNEGEKFKQIS

QAYEVLSNPEKRSLYDRGGEQALKEGGIGGGGFTSPMDIFETIFGTSRSRG

PARGKDVHHQLSVTLEELYKGCERKLSLEKNIICEDCKGRGGKKGANEE

VCSPCRGTGRITEILQLRPGMIQQSQSICGECRGQGRKFSHKDMCRTCMG

RKLVRTRQIIKINIEKGMVDNQKIVLSGEGDQAPGLEAGDVVIILDEKKH

DVFKRNGSNLIMMMHISLVESLCGFQKVIHTLDDRDLVITAIPGEVTKHG

EVKCIMGEGMPQYKNPFEKGQLIVQFLVDFPDSIAADKIAKLEACLPPRP

EVIIPDDAEECTLLPMDPNADARRQDSRNAYDEDEAHDGQGFRTMQCA

SQ

>WangBtHsp90A1

MPEDATMEQAETFVFQAEIAQLMSLIVNTFYSNKEIFLRELISNSSDALDKI

RYESLTDPSRLESGKELFIKIIPNKNDNTLTLIDSGIGMTKADLVNNLGTIA

KSGTKAFMEALAAGADISMIGQFGVGFYSAFLVADTVTVVSKHNDDEQY

IWESSAGGSFTIRPDHSEPLGRGTKIIMHMKEDMTEFLEERKIKEIVKKHSQ

FIGYPIKLLVEKERDKELSEDEEEEEEKKEDKEEDKDTPKIEDAEDDEGKEK

KKKKKTIKEKYTEDEELNKTKPIWTRNPDDITTEEYGEFYKSLTNDWEDH

LAVKHFSVEGQLEFKALLFVPKRAPFDLFENKKKKNNIKLYVRRVFIMDN

CEDLIPEYLNFIKGVVDSEDLPLNISREMLQQNKILKVIRKNLVKKCLELFE

ELAEDKENYQKFYEQFSKNLKLGMHEDTQNRKKLSDLLRYQTSASGDES

SSLKDYVARMKENQKHIYYITGESKDQVANSSFVERVKKRGFEVVYMTEP

IDEYVVQQLKDYDGKNLVSVTKEGLELPEDEEEKKKFEEDKVKFETLCKV

MKDILDKKVEKVVVSNRLVESPCCIVTSQYGWTANMERIMKAQALRDSS

TMGYMAAKKHLEINPDHAVIDALRVKAEAEKNDKSVKDLVMLLFETAL

LSSGFALEDPQVHAARIHRMIKLGLGIDEDETVHVEEEKPDAAMPAADG

DAEDASRMEEVD

>WangBtHsp90A2

MPEDVNMEQAETFVFQAEIAQLMSLIVNTFYSNKEIFLRELISNSSDALDK

IRYESLTDPSRLESGKELYIKIIPNKNDRTLTIIDTGIGMTKADLVNNLGTIA

KSGTKAFMEALAAGADISMIGQFGVGFYSAFLVADTVTVVSKHNDDEQY

VWESSAGGSFTIKSDHGEPLGRGTKIIMHMKEDMTEFLEERKIKDIVKKHS

QFIGYPIKLLVEKERDKELSEDEEEEEVKKEDKEEKEEDKDTPKIEDVEDEE

EGKEKKKKKKTIKEKYTEDEELNKTKPIWTRNPDDITTEEYGEFYKSLTND

WEDHLAVKHFSVEGQLEFKALLFVPRRAPFDLFENKKKKNNIKLYVRRV

FIMDNCEDLIPEYLNFIKGVVDSEDLPLNISREMLQQNKILKVIRKNLVKK

CLELFEELAEDKENFKKFYEQFSKNLKLGIHEDTQNRKKLADLLRYQTSA

TGDDVCSFKDYVARMKENQKHIYYITGESKDQVANSSFVERVKKRGFEVI

YMTEPIDEYVVQQMKDYDGKNLVSVTKEGLELPEDEEEKKKYEEDKVKF

ETLCKVMKDILDKKVEKVIVSNRLVESPCCIVTSQYGWTANMERIMKAQ

ALRDSSTMGYMAAKKHLEINPDHPVMDALRVKAEAEKNDKSVKDLVM

LLFETALLSSGFALEDPQVHAGRIHRMIKLGLGIDEDEPVMVEEEKPDTA

MPAADGDAEDASRMEEVD

>WangBtHsp90B1

MFRGSKLIALSLGLLIFALAGARAEDEIIEEKTIDIDLGASREGSRTDAEVV

QREEERILIDGLNVAQLRELKEKAEKQTFQAEVNRMMKLIINSLYRNKEIF

LRELISNASDALDKIRLLSLTDPGVLDTNSELAIRIKADKDNKVLHITDTGI

GMTKGDLVKNLGTIAKSGTAEFLSQMNEKSEVKQDLNDMIGQFGVGFYS

AFLVADKVFVTTKHNEDQQYIWESDASSFTIAEDPRGTTLKRGTTISLQLK

DEAADFVEIDTLKNLIRKYSQFINFPIYIWSSKTVTEEVPVDEEKSEDSKDET

PTEKAEEDVEVEESKDEEPKTKKVEKTVWDWELLNDHKPIWTRKPNEIE

DAEYNEFYKSMTKDKNDPLAKTHFVAEGEVTFKSLLFVPNAQPADSFNK

YGTVSDNIKLYVRRVFITDEFTDLLPRYLNFLRGVVDSDDLPLNVSRETLQ

QHKLLKVIRKKLVRKALDMIKKLDPETYKKFYQEYSTNVKLGIVEDPANR

VRLAKLLRFYSSNQSGLTSLADYVTRMKPKQDHIYFIAGTSRDEVEKSPFV

ERLISKGYEVLYLIEAIDEYAVSSLPEFEGKKFQNVAKEGLTLDNPEKLKEV

SEQFEPLTKWLSENALKGKISKAVLSERLVDTPCALVAGMFGWTGNMER

LALSNAHQKSDDPQREHYMKQRKTLEVNHKHPLVRELLKRVKDDPEDE

KAKDIALMMFRTATLRSGFMLQDSAEFAQSVELLMRQSLGISPDEKVLED

EDDEETKNEDGSKSEDGSEDQDEADSTDDHEHEEL

>WangBtHsp70-1

MAAMSVIGIDFGNESCYVAVARAGGIETIANDYSLRATPSCVAFSEKTRIL

GVAAKNQLVTNMKNTVYGFKRLLGRQYKDPFIQKDLQSLTYQTVETPSG

GVGIKVNYLNEPHVFHPEQITAMLLTKLKAISEEALNTKINDCVISVPSYF

TNTERKALLNAASIAGLNVLRLFNETTATALAYGIYKQDLPPPEEKPRNV

VFVDCGYSSLQVSACAFHKGKLKMLACAADPDLGGHSFDVILAEHLSKD

LKARYNINPMTNARAYTRLLTEVDKLKKQMSANSTKLPLNIECFMEDKD

VHCDMKRADMEELCAGLFQRVEKTLRQCLEDSKLKLEDIHAVEIVGGSS

RVPAIKQLIEVVFNKPASTTLNQDEAVARGCALQCAMLSPAVRVRDFAV

TDIQMYPVHINWDGHDAQDQGEMEVFSKNHAAPFSKVLTLYKKESFNL

KAFYPPNSVPYPDPRIGEYCIKNIKPTAQGGTQKVKLKIRINIHGIVSVSSAS

LIEQIKGSAEPMDVEVNEEEHKEKEQPAQETTQEQQQPPEANANSQDAQ

PNGPAEDDDAEKKEKKRKTVKSVDLPIEEFLPGFSSSEVSSFFEAEGQMM

AADRQEKDRVDARNSLEEYVYELRGKLSEELSAYVVEKDRSSLVEQLDQ

MENWLYEEGEECVKQVYMDKLGSLRSIGDAIYQRKMEYEARPKALEELS

VAIQLAFKNLEQYKAREDKLKNLDPATVEKSEQNIRNLHHWCEEKRAIL

SATPLHQNPPVSVAEILAEKEKAVSEIKLLSTMPKVAPEPAKQQPPPEQSQ

ESTQDEKMKTENGEQD

>WangBtHsc70-1

MRLLFLSGAFCLLACVAFAKEEKKDKEDFGTVVGIDLGTTYSCVGVYKN

GRVEIIANDQGNRITPSYVAFTAEGERLIGDAAKNQLTTNPENTVFDAKR

LIGRDWNDPTVQQDIKFFPFKVKEKNSKPHIEVSTSQGVKIFAPEEISAMV

LSKMKETAEAYLGKPVTHAVVTVPAYFNDAQRQATKDAGAIAGLVVM

RIINEPTAAAIAYGLDKKEGEKNVLVFDLGGGTFDVSLLTIDNGVFEVVST

NGDTHLGGEDFDQRVMDHFIKLHKKKKGKDVRKDNRAVQKLRREVEK

AKRALSSAHQVRIEIESFFDGEDFSETLTRAKFEELNLDLFQSTLKPVQKVL

EDADMNKKDVDEIVLVGGSTRIPKVQQLVKEYFNFKEPSRGINPDEAVA

YGAAVQAGVLSGEQDTDAIVLLDVNPLTLGIETVGGVMTKLIPRNTVIPT

KKSQIFSTASDNQHTVTIQVYEGERPMTKDNHLLGKFDLTGVPPAPRGVP

HIEVTFEIDANGILQVSAEDKGTGNREKIVITNDQNRLTPDDIERMIKEAE

KFADDDKKLKERVEARNELESYAYSLKNQLADKEKLGSKLSDEEKTKME

EAIDEKIKWLEENQDTDGAEYQKQKKDLEQIVQPIIAKLYAGQGPPPPAG

GDSDDLKEEL

>WangBtHsp70-2

MPGKTPAIGIDLGTTYSCVGVWQQGKVEIIANDQGNRTTPSYVAFTDTER

LIGDAAKNQVAMNPKNTVFDAKRLIGRKFDDPKIQADMKHWPFTVVN

DCSKPKIQVEFKGETKRFAPEEISSMVLTKMKETAEAYLGGKVRDAVITVP

AYFNDSQRQATKDAGAIAGLNVLRIINEPTAAALAYGLDKNLKGERNVL

IFDLGGGTFDVSILTIDEGSLFEVRSTAGDTHLGGEDFDNRLVNHFVEEFK

RKYRKDLRTNPRALRRLRTAAERAKRTLSSSTEASIEIDALMDGIDYYTKV

SRARFEELCADLFRSTLHPVEKALNDANMDKGSIHDVVLVGGSTRIPKVQ

SLLQNFFGGKTLNFSINPDEAVAYGAAVQAAILSGDTSSAIQDVLLVDVA

PLSLGIETAGGVMTKIVERNARIPCKQSQTFTTYADNQPAVTIQVYEGERA

MTKDNNLLGRFDLTGIPPAPRGVPKIDVTFDLDANGILNVSAKENSTGRE

RNIVIKNDKGRLSREEIDRMVNEAEQYKEEDDKQRARVAARNQLESYIFN

VKQAAEEAGDKLSAADKQSILEDCQAALQWLDNNTLADVEEFNYKLQE

LQKKCSPIMSKMHQSSQNSQGMPGKGGMPGHGMPGPGGMPSYGMPG

QGGMPSYGMPGQGGMPSYGMPGQGGMPSYGMPGQSRTPGYGGYHQN

TSGGPTIEEVD

>WangBtHsp70-3

MSRFTVFGIYVGNTSASIAICKEDGKVEVLANAAGERSTPAVVAVTEKEQI

VGNAAKSSMITQTNAGTIITNNKLLMDLSLDESDLESIIAKNPCTIIQKSN

HVAYEINFGNKTKTFTPAMINTSIYSLLYNIAKSAIHGECDEICCVLVVPTY

YSAESRQYIKQSAVEAGWKVLQIVNQPSVAPLAYHSFKEASPETKYICVYR

VGGVSCDAAILRLRNGFIQILASETNFDIGGHELVSKLIQCFCEEMKRKYK

VDPNESKRSIRKLRSAAETCIKVLSTLSSASVSVDSLFEGIDFNYQMSRARF

ESLIPSLFGPLDAPINDALQSAQLQATDINEVILCGEPLKTPRLQSHVKSLF

PNAEVLSSINPDEVLARGAARQAGFITEFCDLHKSIPEPVTDVTFITEPITV

QIQSSNNEEIVFEKFSSLPISKTVHLKDNKSEIIVTAAQSCSAESKLNGKVTI

PSSKDSSDLYLDVEINAEQAIEIHLLNKEMEELQMYTMDLVAVPAS

>WangBtHsc70-2

MAKAPAIGIDLGTTYSCVGVFQYGKVEIIANDQGNRTTPSYVAFTDTERLI

GDAAKNQVAMNPNNTIFDAKRLIGRRFDDATVQADMKHWPFTVINDN

TKPKIQVEYKGEAKQFFPEEISSMVLIKMKETAEAYLGKTVTNAVITVPAY

FNDSQRQATKDSGAIAGLNVLRIINEPTAAAIAYGLDKKASGSGERNVLIF

DLGGGTFDVSILTIEDGIFEVKSTAGDTHLGGEDFDNRMVNHFAQEFKRK

YKKDLTTNKRALRRLRTACEKAKRILSSSSQTSIEIDSLFEGIDFYTSITRARF

EELNADLFRSTMEPVEKSLRDAKMDKAQIHDIVLVGGSTRIPRVQKLLQD

FFNGKELNKSINPDEAVAYGAAVQAAILHGDKSEEVQDLLLLDVTPLSLG

IETAGGVMTVLIKRNTTIPTKQTQTFTTYSDNQPGVLIQVYEGERTMTKD

NNLLGKFELTGIPPAPRGVPQIEVTFDIDANGILNVTAIEKSTGKENKITIT

NDKGRLSKEDIEKMVSDAEKYKHEDEKQRQVIAAKNSLESYCFNMKSTM

EDDKLKDKISESDKTAIMEKCNEVIRWLDSNQLADKEEFEHKQKELEALC

NPIITKLYQGAGGAEGMPGFPGGMPGAPGAGGAGAAAGGPTIEEVD

>WangBtHsp70-4

MAKAPAVGIDLGTTYSCVGVWQQGKVEIIANDQGNRTTPSYVAFSDTER

LIGDAAKNQVAMNPQNTIFDAKRLIGRRFDDPKIQADMKHWPFKVIND

CGKPKLQVEFKGETKTFAPEEVSSMVLTKMKETAEAFLGTKVKDAVITVP

AYFNDSQRQATKDAGAIAGLNVLRIINEPTAAALAYGLDKNLKGERNVL

IFDLGGGTFDVSILTIDEGSLFEVRATAGDTHLGGEDFDNRLVNHLAEEFK

RKYRKDLRGNNRALRRLRTSAERAKRTLSSSTEASIEIDALMDGIDFYTKV

SRARFEELCSDLFRSTLHPVEKALADAKMDKSSIHDVVLVGGSTRIPKIQS

LLQNFFCGKALNFSINPDEAVAYGAAVQAAILSGDTSSAIQDVLLVDVAP

LSLGIETAGGVMTKIVERNARIPCKQSQTFTTYSDNQPAVTIQVYEGERA

MTKDNNLLGTFDLTGIPPAPRGVPKIDVTFDLDANGILNVSAKENSTGKS

KNIVIKNDKGRLSREEIDRMVNEAEKYKEEDEKQRAKIAARNQLESYVFN

VKQAVDEAGDKLPESDKQLVHDECQAALSWLDNNTLADVEEFNYKLQ

EVQKKCSPIMSKMHGAGQGGMPGGMGGMPGGMGGMPGGMGGMPGY

GGQGPTVEEVD

>WangBtHsp70-5

MPGKTPAIGIDLGTTYSCVGVWQQGKVEIIANDQGNRTTPSYVAFTDTER

LIGDAAKNQVAMNPKNTVFDAKRLIGRKFDDPIIQADMKHWPFTVVND

CSKPKIQVEFKGETKRFAPEEISSMVLTKMRETAEAYLGGKVQDAVITVPA

YFNDSQRQATKDAGAIAGLNVLRIINEPTAAALAYGLDKNLKGERNVLI

FDLGGGTFDVSILTIDEGSLFEVRATAGDTHLGGEDFDNRLVNHFVEEFK

RKYRKDLRTNPRALRRLRTAAERAKRTLSSSTEASIEIDALMDGIDYYTKV

SRARFEELCADLFRSTLHPVEKALNDAKMDKGLIHDVVLVGGSTRIPKVQ

SLLQNFFGGKTLNLSINPDEAVAYGAAVQAAILSGDTSSAIQDVLLVDVA

PLSLGIETAGGVMTKIVERNARIPCKQSQTFTTYSDNQPAVTIQVYEGERA

MTKDNNLLGRFDLTGIPPAPRGVPKIDVTFDLDANGILNVSAKENSTGRE

RNIVIKNDKGRLSREEIDRMVNEAERYKEEDDKQRAKVAARNQLESYIFN

VKQAAEEAGDKLSATDKQSIQEECQAALQWLDNNTLADVEEFNYKLQE

LQKKCSPIMSKMHQSGQNSQGMPGGQGMSRQGGMPGQGGMPGYGMP

GQGGMPGYGMPGQGGMPGYGMPGQGGMPGHGGPTIEEID

>WangBtHsp70-6

MPGKTPAIGIDLGTTYSCVGVWQQGKVEIIANDQGNRTTPSYVAFTDTER

LIGDAAKNQVAMNPKNTVFDAKRLIGRKFDDPIIQADMKHWPFTVVND

CSKPKIQVEFKGETKRFAPEEISSMVLTKMRETAEAYLGGKVQDAVITVPA

YFNDSQRQATKDAGAIAGLNVLRIINEPTAAALAYGLDKNLKGERNVLI

FDLGGGTFDVSILTIDEGSLFEVRATAGDTHLGGEDFDNRLVNHFVEEFK

RKYRKDLRTNPRALRRLRTAAERAKRTLSSSTEASIEIDALMDGIDYYTKV

SRARFEELCADLFRSTLHPVEKALNDAKMDKGLIHDVVLVGGSTRIPKVQ

SLLQNFFGGKTLNLSINPDEAVAYGAAVQAAILSGDTSSAIQDVLLVDVA

PLSLGIETAGGVMTKIVERNARIPCKQSQTFTTYSDNQPAVTIQVYEGERA

MTKDNNLLGRFDLTGIPPAPRGVPKIDVTFDLDANGILNVSAKENSTGRE

RNIVIKNDKGRLSREEIDRMVNEAERYKEEDDKQRAKVAARNQLESYIFN

VKQAAEEAGDKLSATDKQSIQEECQAALQWLDNNTLADVEEFNYKLQE

LQKKCSPIMSKMHQSGQNSQGMPGGQGMSRQGGMPGQGGMPGYGMP

GQGGMPGYGMPGQGGMPGHGGPTIEEID

>WangBtHsp70-7

MEKPAIGIDLGTTHSCVGVWQHGKIEIIANDQGMRITPSYVAFADGVRLI

GDSAKIQAATNPENTIFDAKRLIGRKFNDSVVQADIEHWPFAVIDDGGN

PKVRVDHDGEVKEFAPEEISSMVLSKLKETAEAYLDCEVTDAVITVPAYF

NDSQRQATKDAGAIAGLNVLRIINEPTAAALAYGLDMKGKRNILIFDLG

GGTFDVSILTIDEGSLFEVKSTAGDTHLGGEDFDSLLVNYLIEEFKRRYKKD

FAPNPRTLSRLRAHAERAKCTLSAAPTTNISIDALFDGIDFHTLVTRAKFE

NLCADLFQRTLTIVEKALMDAELGKDAIDDVVVVGGSSRIPKVQSLLQDF

FGGKTLKHTIHPDEAVAYGAAVQAASLSGNEDLDIKDIVLHDVTPLSLGI

DIENDVFSVLIKRNTRIPCKITRNYTTGVDNQVNIRVEIYEGERAVASENH

LLGKLLLQGIKKAPRGVPNVDVSFDLNAEGILSVSAEESGTGNSANITITN

EKGRLSKEDIIRMIREAKLFKEKDEKHLARSRARNQLEDYTYKMMQELK

AAESELSQADKGCMKEESDKIWDWLKKNPKAGLGEYKQKLQDIQERCQ

PIMKKIHTEDALLD

>WangBtHsp70-8

MEKPAIGIDLGTTHSCVGVWQHGKIEIIANDQGNRITPSYVAFSDERLIGD

AAKTLAPSNAKNTIYDAKRLIGRKFDDPLVQADMKHWPFTVINDAGNA

KLRVEYNGMERVFAPEEISSMVLSKLKDTAKVYLDCEITDAVITVPAYFN

DSQRQATKDAGTIAGLNVLRIINEPTAAAIAYGLDQNLKGKRNILVFDLG

GGTFDVSILTIDEGSLFEVKSTAGDTHLGGEDFDSLLVNHLAKEFKLKHKI

DLTSNSRALCRLRSAAERAKCTLSAASTANIFIDGLFDGIDFHSFLTRAKFE

DLCGDLFRKTLTIVEKAISDAKLEKDAIDDVVLVGGSSRIPKVQSLLQNFF

DGKSLKHSIHPDEAVAHGAAVLAASLSGNNDLKIKDVVLRDVTPLSLGI

HMKNDVFSVIIERNSRIPCKKTKTYYTTEDNQYTAGIDIYEGERTVASQNH

LLGKLTLEGIQKAPKGETKVAVTFDLNAEGILSVSAEERGTDKSENITITNE

KGRLSKEDIRRMIKEAKRYKEEDDKHLARSRARNQLEDYTYKMMQELEA

AESELSQADKSCLKDESDKIWDWLKENPKAGLGEYKQKLQDIQEKCQPI

MKKIHNEDALLD

>WangBtHsp70-9

MEKRAIGIDLGTTHSCVCVWQNGKMEVISNEQGNRVTPSYVAFSEVRLIG

DTAKMLAPINPKNTIFDVKRLIGRKFDDPLVQADMKHWPFTVINDAGNP

KVSVEYQGVDKVFAPEEISSMVLSKLKETAEVYLNRKVTDAVITVPAYFN

DSQRQATKDAGAIAGLNVLQIINEPTSAAIAYGLDQNLKSKRNILVFDLG

GGTFDVSVLTVDEGSLFEVKSTAGDSHLGGEDFDSLLMNYLIEEFKLKHS

KDLASNPRALSRLRAAAERAKCTLSSAPTANIMIDSLFDGIDFYTQVTRPK

FEDLCADLFQRTLTTVEQALTDAKIEREAIDDVVLVGGSTRIPKVQLLLQN

FFDGKTLNHSIHPDEAVAYGAAVQAASLSGNEDLEIKDIVLRDVTPLSLG

VFTKNDIFSVIIQRNTRIPCKIRREFVTVGDNQESVAIHIYEGERTVATENH

LLGKVLLQGIKRAPKGVTKVDVSFDLNEEGILSVSVEEKGTNKSEQITITNE

KGRLSKGDIRRMIKEAKSFKEKDEKHLARSRARNQLEDYTYKMMQDFKV

AESQLSQADKSSMKEESDKIWDWLKKNPKAGLGQYKRKLQDIQEKCQPI

MKKIHKEDALLD

>WangBtHsp70-10

MEKRAIGIDLGTTHSCIGVWQNGKMEIISNEQGNRITPSYVAFSDERFIGD

TAKMQAPINPKNTIFDVKRLIGRKFDDPLVQADMKHWPFTVINDAGNP

KVRVEYQGVEQVFAPEEISSMVLSKLKETAEVYLNREVTDAVITVPAYFN

DSQRQATKDAGTIAGLNVLRIINEPTAAAIAYGLDQNLKSKRNILVFDLG

GGTFDVSVLTVDEGSLFEVKSTAGDTHLGGEDFDSLLVNYLIKEFKLKYK

KDLQSNPRALSRLRAAAERAKCTLSSALSANIMIDSLFDGIDFYTLVIRPKF

EDLCADLFQRTLTTVEQALTDAKLEREAIDDVVLVGGSTRIPKVQSLLEDF

FGGKSLKHSIHPDEAVAHGAAVLAASLSGNDDIKIKDVVLRDITPLSLGIN

TKNNVFSVIIERNTRIPCKKTRTYHTTEDNQDGLEFSIYEGERAVASENHL

LGELTLEGIQKAPKGETKVAVTFDLNEEGILSVSVAEEGTDKSEHITITNEK

GRLSDKEIRRMIQEAKRFKEEDDKHLARSRARNQLGDYTYKMTQELKAA

ESKLSKTDKSRLKEESDKIWDWLQKNPKAGLGEYKQKLQDIQEKCQSIM

MKIHKDDALLD

>WangBtHsp70-11

MPGKTPAIGIDLGTTYSCVGVWQQGKVEIIANDQGNRTTPSYVAFTDTER

LIGDAAKNQVAMNPRNTVFDAKRLIGRKFDDPKIQADMKHWPFTIVSD

CGKPKIQVEFKGEAKKFAPEEISSMVLTKMKETAEAFLGGTVRDAVITVP

AYFNDSQRQATKDAGAIAGLNVLRIINEPTAAALAYGLDKNLKGERNVL

IFDLGGGTFDVSILTIDEGSLFEVRATAGDTHLGGEDFDNRLVNHFVEEFK

RKYRKDLRTNPRALRRLRTAAERAKRTLSSSTEASIEIDALLDGIDYYTKVT

RARFEELCADLFRSTLHPVEKALNDANMDKGTIHDVVLVGGSTRIPKIQS

LLQNFFGGKTLNLSINPDEAVAYGAAVQAAILSGDTSSAIQDVLLVDVAP

LSLGIETAGGVMTKIVERNARIPCKQSQTFTTYSDNQPAVTIQVYEGERA

MTKDNNLLGRFDLTGIPPAPRGVPKIDVTFDLDANGILNVSAKENSTGRE

RNIVIKNDKGRLSREEIDRMVNEAERYKEEDDKQRAKVAARNQLESYIFN

VKQAAEEAGDKLSAEDKQSVQEECQAALQWLDNNTLADVEEFNYKLQ

ELQKKCSPIMSKMHHSGQNGQGMPGQGGMPGYAGHQRSTSGGPTIEEV

D

>WangBtHsp70-12

MVKAPAIGIDLGTTYSCVGVWQQGKVEIIANDQGNRTTPSYVAFSDTERL

IGDAAKNQVAMNPQNTIFDAKRLIGRRYDDPKIQDDMKHWPFKVINDC

GKPKLQVEFKGETKTFAPEEVSSMVLTKMKETAEAFLGGQVKDAVITVP

AYFNDSQRQATKDAGAIAGLNVLRIINEPTAAALAYGLDKNLKGERNVL

IFDLGGGTFDVSILTIDEGSLFEVRATAGDTHLGGEDFDNRLVNHLAEEFK

RKYRKDLRGNNRALRRLRTAAERAKRTLSSSTEASIEIDALMDGIDYYTK

VSRARFEELCSDLFRSTLHPVEKALADAKMDKGSIHDVVLVGGSTRIPKIQ

SLLQNFFCGKTLNLSINPDEAVAYGAAVQAAILSGDTSSAIQDVLLVDVA

PLSLGIETAGGVMTKIVERNARIPCKQSQTFTTYSDNQPAVTIQVYEGERA

MTKDNNLLGTFDLTGIPPAPRGVPKIDVTFDLDANGILNVSAKENSTGKS

KNIVIKNDKGRLSREEIDRMVNEAEKYKEEDERQRAKIAARNQLESYVFN

VKQAVDEAGDKLPESDKQLVRDECQAALSWLDNNTLADVEEFNYKLQE

VQKKCSPIMSKMHGAGQGGMPGGMGGMPGGMGGMPGGFPGGMPGQ

GGQGPTVEEVD

>WangBtHsp70-13

MKTPAIGIDLGTTYSCVGVWQQGKVEIIANDQGNRTTPSYVAFTDTERLI

GDAAKNQVAMNPKNSVFDAKRLIGRKFDDPKIQADMKHWPFTVINDC

GKPKIQVEYKGEVKKFAPEEVSSMVLTKMKETAEAFLGDKVRDAVITVP

AYFNDSQRQATKDAGAIAGLNVLRIINEPTAAALAYGLDKNLKGERNVL

IFDLGGGTFDVSILTIDEGSLFEVKSTAGDTHLGGEDFDNRLVNHLADEFK

RKYKKDLKTNPRALRRLRTAAERAKRTLSSSTEASIEIDALFDGVDFYTKV

SRARFEELCSDLFRSTLAPVEKALTDAKMDKSAIHDVVLVGGSTRIPKVQS

LLQNFFCGKSLNLSINPDEAVAYGAAVQAAILSGDTSSQIQDVLLVDVAP

LSLGIETAGGVMTKIIERNARIPCKQSQTFTTYADNQPAVTIQVYEGERAM

TKDNNLLGTFDLTGIPPAPRGVPKIDVTFDLDANGILNVAAKENSTGKSK

NIVIKNDKGRLSKEEIDRMVNEAERYKEEDDKQRARVNARNQLEAYIFN

VKQAVEEAGSKLSEVDKNSVKEQCDNTLRWLDNNSLAEKDEYDHKLQE

VQKVCMPIMSKLHGAGGGAGAPGAGGPTVEEVD

>WangBtHsc70-3

MLYVARVVGRKTLHCCLFNPSYTTSENFSIYLSKTPVIFNSRIYEFQRRFKSE

QVKGTVIGIDLGTTNSCVAVMEGKTPKVIENSEGSRTTPSVVAFSKDGERI

VGMPAKRQAVTNSANTFYATKRLIGRRFDDAEVKKDMKTLSYKIVKASN

GDAWVQATDGKMYSPSQIGAFVLVKMKETAESYLGTPVKNAVITVPAYF

NDSQRQATKDAGQIAGLNVLRVINEPTAAALAYGMDKTDDKIIAVYDL

GGGTFDISILEIQKGVFEVKSTNGDTFLGGEDFDNVLVNHLVTEFKKEQGI

DVTKDAMAMQRLKEAAEKAKIELSSSLQTDINLPYLTMDASGPKHMNL

KLTRSKFESLVADLIKRTVAPCQKAIQDAEVGKSDISEVLLVGGMTRMPK

VQQTVQEIFGRQPSRAVNPDEAVAVGASVQGGVLAGDVTDVLLLDVTP

LSLGIETLGGVFTRLISRNTTIPTKKSQVFSTAADGQTQVEIKVYQGEREMA

SNNKLLGQFTLVGIPPAPRGVPQIEVTFDIDANGIVHVSARDKGTGKEQQ

IVIRSSGGLSKDEIENMVKSAEEYAQSDKIKKERVEAINQAEGIIHDTESKM

TEFKDQLPSEECDKLREQMSKVRDILTKGEEADPEEIRTATNELQQASLKL

FEMAYKKMAADRDGSSSSSSSEEQSSEEKGEEKEKKENKQ

>WangBtHsp70-14

MGKQVPAVGIDLGTTYSCVGVWQHGKVEIIANDQGNRTTPSYVAFNDT

ERLIGDAAKSQVAMNPKNTVFDAKRLIGRRFDDPKIQDDIKHWPFKVISD

SGKPKIQVEFKGEQKIFAPEEISSMVLTKMKEVAEVYLGGKVSEAVITVPA

YFNDSQRQATKDAGAIAGMNVLRIINEPTAAALAYGLDKNLKGERNVLI

FDLGGGTFDVSILSIDEGSLFEVKSTAGDTHLGGEDFDNRLVTHFSEEFKR

KYKKDLRGNARALRRLRTACERAKRTLSSSTEASLEIDALHEGVDFYAKIT

RARFEELCMDLFRNTLAPVERALTDAKMDKGSIHDVVLVGGSTRIPKVQ

KMLQDFFCGKSLNLSINPDEAVAYGAAVQAAILSGDQSAEIQDVLLVDV

TPLSLGIETAGGVMTKIVERNARIPCKQTQTFTTYSDNQPAVTIQVFEGER

SMTRDNNLLGTFNLSGIPPAPRGVPKIEVTFDLDANGILNVSAKDNSSGK

MERITIKNDKGRLSKEDIEKMLADAEKYKHEDEKQKERVTARNQLEGYT

FNVKQAVEEAGSKLSETDKKAVLDKCSAVLTWLDTNSLAEKEEYEEKLK

DLQKDCAPVMTKLHQQGGKGPTVEEVD

>WangBtHsp60

MYRLPSVLRSSAARQLNPSLARFYAKDVRFGPEVRGLMLQGVDILADAV

AVTMGPKGRNVILEQSWGSPKITKDGVTVAKGVELKDKFQNIGAKLVQ

DVANNTNEEAGDGTTTATVLARAIAKEGFEKISKGANPIEIRKGVMLAVE

TVKNNLKSLSKQVTTPEEIAQVATISANGDGAIGNLISDAMKKVGKEGVI

TVKDGKTLEDELEVIEGMKFDRGYISPYFINTTKGAKVEFQDALLLLSEKKI

SSIQSIIPALELANSKRRPLVIIAEDIDGEALSTLVVNRLKIGLQVAAVKAPG

FGDNRKSTLQDLAIATGGLVFGDEGTAIKLEDVQLHDLGEVGEIVITKDD

TLILKGKGKKEEIDRRADQLRDQIADTTSEYEKEKLQERLARLASGVALLK

VGGSSEVEVNEKKDRVTDALNATRAAVEEGIVPGGGLALLRCIPKLDAIS

SKNEDQKTGIEIVKRALRMPCMTIASNAGVDASMVVAKVLESPPEIGYDA

LNNEYVNMIEKGIIDPTKVVRTALTDAAGVASLLTTAEAVVTEIPKEEPP

MGGMGGMGGMGGMGGMGGMGGMM

>WangBtsHsp21.5

MQSALRAALLNELVDELTNPLSALTDQNFGIGLLTDELNSRPRYQHTALS

LPLAAGYVRPWRISPAQQSGVSNIHHDKAAFKVNLDVQQFQPEEVSVKV

VDGFLVVEAKHEERQDKHGYISRSFTRRYKLPKDINEDAIVSSLSSDGVLTI

SATVKNQLPSGERQIPITQTNQPALKKAKSDAPQENGKGDKMES

>WangBtsHsp22.6

MADGIKRDIPIRLGDFSVIDSEFSNIRERFDAEMKKMEDEMTKFRSELMNR

ESNFFKTTTRSTKIGSWDYSSTNSITSNSNNTLSEPKPNLWLESINSPLIQED

GDNKMLKLRFDVSQYQPEEIIVKTVDNKLLVHAKHEEKTDSKSVYREYN

REFLLPKGTNPESIKSSLSKDGVLTVEAPLPALGAPEKLVPIAHH

>WangBtsHsp21.6

MSLAPYVFRDWWDDLEFGRPSRLIDQHFGLGLRKDDLFADWPSLVRPSG

AGYLRPFRNLLRQTSGSSNITADDKSVQVILDVQQFAPNEIVVKTSNGAIV

VEGKHEEKQDEHGFVSRQFTRRYVLPKDVEIENITSSLSSDGVLTISVPKKN

PAIPGGEKVVPIIQTHAPALKPAPQPSSSPASSPNVTEIKVQHQ

>WangBtsHsp19.4

MALLPYLLEELNRPTIYDQNFGLGLFNDDFPSAVGSLRPYYLRPWRLFPH

DESGISSVQHDKDGFKVNLDVQQFKPEEVNVKIADNYVIVNAKHEERSD

EHGFISREFTRRYLLPKDVNAEALTSSLSSDGVLSIQAPPKAITNDKGNER

QIPVTKTNAPAIKQQQQQKK

>WangBtsHsp19.5

MSLLPLLLSELEDTRRPSIYDPYYGLGLGDLLAVPARSGYLRPWRLAPHN

DSGISNIQNDKKEFKVNLDVQQFKPEEINVKTAGDYITVEAKHEERSDEH

GFVSRQFTRRYLVPQDVNKDAITSTLSSDGVLTIHAPKKAIAAKDERNISIT

QTNKPALKQGKKDGKPEKMET

>BaiBtsHsp19.5

MALLPYLLEELNRPTIYDQNFGLGLFNDDFPSAVGSLRPYYLRPWRLFPH

DESGISVQHDKDGFKVNLDVQQFKPEEVNVKIADNYVIVNAKHEERSDE

HGFISREFTRRYLLPKDVNAEALTSSLSSDGVLSIQAPPKAITNDKGNERQI

PVTKTNAPAIKQQQQQKK

>BaisBtHsp19.2

MSLLPYLLEELNRPTVYDQNFGVGLWNDDLPSITAIRPFYMRPWRLLPQN

ESGTSVQHDKDGFKVNLDVQQFKPEEVNVKVSDNYVTIHAKHEERSDE

HGFISREFTRRYLIPKDVNAEALTSSLSSDGVLSVHAPPKAITNENGSERQI

PITKTNAPAIKQEQAKKK

Athalia rosae

>ArHsp90|XP_012250991.1

MPEQMETAGEVETFAFQAEIAQLMSLIINTFYSNKEIFLRELISNSSDALDK

IRYESLTDPSRLDSGKDLHIKIIPNKNDGTLTLIDTGIGMTKADLVNNLGTI

AKSGTKAFMEALQAGADISMIGQFGVGFYSAYLIADKVTVVSKHNDDEQ

YLWESSAGGSFTVRPDPGEPLGRGTKIVLHIKVDQTEFLEESKIKEIVKKHS

QFIGYPIKLLVEKEREKELSDDEAEAEADPEVKKEEDDGKPKIEDVGEDG

DEDKKEKPKKTKTITEKYTEDEELNKTKPIWTRNADDISQEEYGEFYKSLT

NDWEDHLAVKHFSVEGQLEFRALLFVPRRMPFDLFENKKRKNNIKLYVR

RVFIMDNCEELIPEYLNFMKGVVDSEDLPLNISREMLQQNKILKVIRKNLV

KKCLELFEELTEDKESFKKFYEQFSKNIKLGIHEDSTNRSKLADLLRYHTSA

SGDEVCSLKDYVGRMKPNQKHIYYITGESKEQVANSSFVERVKKRGFEVI

YMTEPIDEYVVQQMKEFDGNQLVSVTKEGLELPEDDAEKKKREDDQAKF

ENLCKIMKNILDTKVEKVVVSNRLVDSPCCIVTSQYGWTANMERIMKAQ

ALRDTSTMGYMAAKKHLEINPDHSVIETLRQKAEADKNDKAVKDLVVL

LFETSLLSSGFSLDEPQVHAARIYRMIKLGLGIDEEEPTADEEPVAAEVTPV

EGDNEDASRMEEVD

>ArHsp90|XP_012255700.1

MAAALRVSCGIQRAIKFGRFLTRPSSFRPKNLTNSNRYREPSAFSQNAHIR

YLSSHAATAESSPDPEHHSIIKDTEKATGQSERHEFQTETRQLLDIVAKSLY

SDKEVFIRELISNASDALEKLRYLGLSDSANVPAAASQNLEIHIATDKQNR

ILTIQDTGVGMTREELTSNLGTIARSGSKAFLEQVKNKQGSPGDPSAIIGQF

GVGFYSAFMVADKVEVFTKSYQKDSEGLHWVSDGSGTYEISAAEGVQSG

TKIVLHLRTDCREFSDDATINNVIKKYSNFVGSPIFVNGKRANVIQPLWM

LEPKDVKPEDHVQFYRFVGNSFDEPRFTLHYSTDVPLSIRALLYFPEGKPG

LFEMSRDTDIGVSLYSRKILIKSRADNILPKWLRFVKGVVDSEDIPLNLSRE

LLQNSALIGKLRNVLTSRILRFLYDRSTKQPEDYAKFYKDYGLFLKEGIVTS

HDQQEKEEISKLLRFESSTSKPGELVSIPQYLSRLAEGQRDVYYLAAPSRTL

AEQSPYYEALKKRNVEVLFCYEPYDELVLMQLGQFNSHPLTSVEKEMRH

NKDDESQPEFGGLEKSEADRLVNYIEKTLKGKAFSVKVTNRLESHPCVVT

VEDMAAARHFIRTQSHQLNSESRFTLLQPRLEINPKHPIIKKLAQLTNSDQ

KLADLVTEQLFANAMVGAGLIEDPRTLLSSMNELLTLALEKH

>ArHsp90|XP_012258880.1

MPEEDSSMAENGEVETFAFQAEIAQLMSLIINTFYSNKEIFIRELISNSSDAL

DKIRYESLTDPSKLESCKELYIKLIPNKNDRTLTIIDTGIGMTKADLVNNLG

TIAKSGTKAFMEALQAGADISMIGQFGVGFYSAYLVADKVTVSSKHNDD

EQYLWESSAGGSFTVRPDTGEPLYRGTKIVLHIKEDQSEYLEESKIKEIVKK

HSQFIGYPIKLVVEKERDKELSEEEEEEEEDEKPAKDEKDKPKIEEVEDDED

EEKSTDKKKKKKTVKEKYTEDEELNKTKPIWTRNPDDITQEEYGEFYKSLT

NDWEDHLAVKHFSVEGQLEFRALLFVPRRAPFDLFENKKKKNNIKLYVR

RVFIMDNCEDLIPEYLNFIKGVVDSEDLPLNISREMLQQNKILKVIRKNLV

KKCLELFDELAEDKESYKKFYEQFSKNLKLGIHEDSQNRKKLSELLRYYTS

ASGDEKCSLKDYVGRMKEHQKHIYFITGESKEQVSNSAFVERVKKRGFEV

VYMTEPIDEYVVQQMKEYDGKQLTSVTKEGLELPEDDAEKKKREDDKAK

FESLCKVMKDLLDKKVEKVVVSNRLVDSPCCIVTSQYGWTANMERIMKA

QALHDTSTMGYMAAKKHLEINPDHPIIESLRQKAEADKNDKAVKDLVM

LLFETALLASGFGLEDPQVHASRIHRMIKLGLGLDEDEAPEEEEKMVEEV

PPLEEDADEAMRMEEVD

>ArHsp70|XP_012253227.1

MPAIGIDLGTTYSCVGVWQQGKVEIIANDQGNRTTPSYVAFTDTERLIGD

AAKNQVAMNPSNTVFDAKRLIGRRFDDPKVQADAKHWPFRVVDEAGK

PKIQVEFRGETKRFNPEEISSMVLTKMKETAEAYLGQKVRDAVVTVPAYF

NDSQRQATKDAGAIAGLNVLRIINEPTAAALAYGLDKNLKGERNVLIFD

LGGGTFDVSILSIDEGSLFEVRATAGDTHLGGEDFDSRLVDHFAAEFERKY

RKNLRKNPRAVRRLRTAAERAKRTLSSSTEASVEIDALFEGIDFYTKVSRA

RFEELCADLFRGTLAPVEKALADAKMDKGAIHDIVLVGGSTRIPKIQSLL

QNFFCGKQLNLSINPDEAVAYGAAVQAAILSGEGDTCSKLQDVLLVDVA

PLSLGIETAGGVMTNIIERNARIPCKQTQTFTTYADNQPGVSVQVFEGERA

MTKDNNLLGRFELSGIAPAPRGLPKIEVTFDLDANGILHVTAKDTASGRS

SDVRITNDKGRLSREEIDRMLAEADRYKEQDREQRERVAARNELEGYVFT

VKQALQEHAERLPQEDREAVATLCEETVRWLDNNSLAEKDEYRDKLEA

LQKRCSAVMAKLHQGAGNAAPGAQDCGAQFRQAGGNHSGPTVEEVD

>ArHsp70|XP_012253231.1

MPAIGIDLGTTYSCVGVWQQGKVEIIANDQGNRTTPSYVAFTDTERLIGD

AAKNQVAMNPSNTVFDAKRLIGRRFDDPKVQADAKHWPFRVVDEAGK

PKIQVEFRGETKRFNPEEISSMVLTKMKETAEAYLGQKVRDAVVTVPAYF

NDSQRQATKDAGAIAGLNVLRIINEPTAAALAYGLDKNLKGERNVLIFD

LGGGTFDVSILSIDEGSLFEVRATAGDTHLGGEDFDSRLVDHFAAEFERKY

RKNLRKNPRAVRRLRTAAERAKRTLSSSTEASVEIDALFEGIDFYTKVSRA

RFEELCADLFRGTLAPVEKALADAKMDKGAIHDIVLVGGSTRIPKIQSLL

QNFFCGKQLNLSINPDEAVAYGAAVQAAILSGEGDTCSKLQDVLLVDVA

PLSLGIETAGGVMTNIIERNARIPCKQTQTFTTYADNQPGVSVQVFEGERA

MTKDNNLLGRFELSGIAPAPRGLPKIEVTFDLDANGILHVTAKDTASGRS

SDVRITNDKGRLSREEIDRMLAEADRYKEQDREQRERVAARNELEGYVFT

VKQALQEHAERLPQEDREAVATLCEETVRWLDNNSLAEKDEYRDKLEA

LQKRCSAVMAKLHQGAGNAAPGAQDCGAQFRQAGGNHSGPTVEEVD

>ArHsp70|XP_012255909.1

MPAIGIDLGTTYSCVGVWQQGKVEIIANDQGNRTTPSYVAFTDTERLIGD

AAKNQVALNPANTVFDAKRLIGRRFDDPKIQDDMKLWPFRVIEEGGKP

KIQVEYRGEVKRFNPEEISSMVLTKMKEIAEAFLGQKIKDAVITVPAYFND

SQRQATKDAGAIAGINVLRIINEPTAAALAYGLDKNLKGERNILIFDLGG

GTFDVSILTIDEGSLFEVKSTAGDTHLGGEDFDSRLVEFFAKEFERKYKKN

LRNNPRALRRLRTASERAKRTLSSSTEASIEIDALFDGIDFYTKVSRARFEEL

CADLFRATLEPVEKALTDAKMDKRSIHDIVLVGGSTRIPKIQNLLQNFFSG

KMLNLSINPDEAVAYGAAVQAAILSGEGDTSTSLQDVLLVDVAPLSLGIE

TAGGVMTNIIDRNARIPCKQTQIFTTYADNQPGVSIQVFEGERALTKDNN

LLGRFELSGISPAPRGVPKIEVTFDLDANGILHVTAKDTASGRSSDVRITND

KGRLSREEIDRMLAEADRYREQDKQQRERIATRNQLESYIFSVKQAVQEQ

GSKLSQEDKNMVFNLCEETIRWLDSNTLAEKEEYQHKLDVVQKQCQPIM

TKLHQGGGTGDRAPNYGSQPDCSSDYSGPTVEEVD

>ArCpn60|XP_012257851.1

MYRLPNMLRGVALRQMNLHSRSYAKDVRFGAEVRALMLQGVDILADA

VAVTMGPKGRNVILEQSWGSPKITKDGVTVAKGVELKDKFQNIGAKLVQ

DVANNTNEEAGDGTTTATVLARAIAKEGFEKISKGANPVEIRRGVMLAV

EKVKDELKNLSKPVTTPEEIAQVATISANGDTAIGNLISDAMKKVGKEGV

ITVKDGKTLIDELEVIEGMKFDRGYISPYFINSSKGAKVEFQDALVIFSEKKI

SSVQSIIPALELANAQRKPLVIIAEDIDGEALSTLVVNRLKIGLQVAAVKAP

GFGDNRKATLQDMAISTGGIVFGDDANLIKLEDVQLSDLGHIGEIVITKD

DTLMLKGKGKKEDVDRRADQIRDQIADTTSDYEKEKLQERLARLASGVA

VLRVGGSSEVEVNEKKDRVHDALNATRAAVEEGIVPGGGTALLRCVPAL

LKMKAANADQETGIKIVADALRMPCLQIAQNAGVDGSVVVAKVSEGTL

GYDAMNNEYVDMIEKGIIDPTKVVRTALTDAAGVASLLTTAEAVVTEIPK

EDPPMPAMGGMGGMGGMGGMGGMGM

>ArsHsp25.7|XP_012255770.1

MADSGIRRDIPIKLREISVLDSEFANIRERFDAEMRKMEDEMSKFRSKLMD

RESNNFFKSTTSTHHMTSEHRTSKSTTGDWETLDSAVPTSKSAFDSFSSKT

TQEQTQNSSLTPHHDSNTWLDGLDSPLIQDEGGNKSLKLRFDVSKYTPEE

IVVKTVDNKLLVHAKHEEKSESKSVYREYNREFLLPKGTNPESIKSSLSKD

GVLTVEAPLPLASGETRIPITHK

>ArsHsp21.6|XP_012255771.1

MADSGIRRDIPIKLREISVLDSEFANIRERFDAEMRKMEDEMSKFRSKLMD

RESNNFFKSTTSKTTQEQTQNSSLTPHHDSNTWLDGLDSPLIQDEGGNKS

LKLRFDVSKYTPEEIVVKTVDNKLLVHAKHEEKSESKSVYREYNREFLLPK

GTNPESIKSSLSKDGVLTVEAPLPLASGETRIPITHK

>ArCpn10|XP_012257765.1

MSVAAAAKRLIPLFDRVLIQRAEAVTKTKGGIVLPEKAQAKVLKGTVVA

VGPGSRNNNGDHVPLSIKVGDTVLLPEYGGTKVELEENKEYHLFRETDIL

AKLES

Drosophila ananassae

>DaHsp90|XP_001956817.1

MPEEAETFAFQAEIAQLMSLIINTFYSNKEIFLRELISNASDALDKIRYESLT

DPSKLDSGKELYIKLIPNKTAGTLTIIDTGIGMTKSDLVNNLGTIAKSGTKA

FMEALQAGADISMIGQFGVGFYSAYLVADKVTVTSKNNDDEQYIWESSA

GGSFTVRADNSEPLGRGTKIVLFIKEDQTDYLEESKIKEIVNKHSQFIGYPIK

LLVEKEREKEVSDDEAEDEKKEDEKKEMDTDEPKIEDVGEDEDADKKDK

DAKKKKTIKEKYTEDEELNKTKPIWTRNPDDISQEEYGEFYKSLTNDWED

HLAVKHFSVEGQLEFRALLFIPRRTPFDLFENQKKRNNIKLYVRRVFIMD

NCEDLIPEYLNFIKGVVDSEDLPLNISREMLQQNKVLKVIRKNLVKKTME

LIEELTEDKENYKKFYDQFSKNLKLGVHEDSNNRAKLADFLRFHTSASGD

DFCSLSDYVSRMKENQKHVYFITGESKDQVSNSAFVERVKARGFEVVYM

TEPIDEYVIQHLKEYKGKQLVSVTKEGLELPEDETEKKKREEDKAKFESLC

KLMKSILDNKVEKVVVSNRLVDSPCCIVTSQFGWSANMERIMKAQALRD

TATMGYMAGKKQLEINPDHPIVETLRQKADADKNDKAVKDLVILLFETS

LLSSGFSLDSPQVHASRIYRMIKLGLGIDEDEPMTTEDNQSAGDAPSLVED

TEDASHMEEVD

>DaHsp90|XP_001961320.1

MSVRAIGVLGQARHLGRLAGTMRQCLRPAWGALHRAVDSKRSPAGFQ

MRGFATETEAKTGSVVDKHEFQAETRQLLDIVARSLYSDHEVFVRELISN

ASDALEKFRYTLLSSGEAELAGMDRPLEIRITTDKPQMQLIIQDTGIGMTK

EELISNLGTIARSGSKQFLEQMKDSQKGASSEASSNIIGQFGVGFYSAFIVA

NKVEVYTRAAVSNAPGLRWSTDGSGSYEIEETSDAELGTKIVLHLKTDCR

EYADEERIKAVIKKYSNFVGSPILLNGKQANEIKPLWLLDPQSISKEQHHD

FYRFISNSFDIPRFTLHYNADVPLSIHALLYFPEGKPGLFEMSRDGNTGVAL

YTRKVLIQSKTEHLLPKWLRFVKGVVDSEDIPLNLSRELLQNSSLIRKLSSVI

STRVIRFLQERAKKQPEEYEAFYRDYGLFLKEGIVTSNDPAEKEEIAKLLRF

DSSKSETQSGRMSLEEYYKTVPADQQNIYYLAAPNRVLAESSPYYESLKKR

NELVIFCYEPYDELVLMQLGKFKSKNLVSVEKEMREESKDVNAATDFGE

GSLLRSELDSLIPWLEGELKGHVVKVKATSRLDTHPCVITVEEMAAARHFI

RTQSHQVPEQNRFALLQPELEINPKHPIIKKLNKLRESDQELAQLIAKQLF

ANAMVGAGLAEDPRMLLTNMNTLLARALEKY

>DaHsp70|XP_001953834.1

MPAIGIDLGTTYSCVGVFQHGKVEIIANDQGNRTTPSYVAFTDSERLIGDA

AKNQVAMNPKNSVFDAKRLIGRKYDDPKIQEDIKHWPFKVISDCGKPK

MSVEFKGEEKRFAPEEISSMVLIKMKETAEAYLGTSVRDAVITVPAYFNDS

QRQATKDAGAIAGLNVLRIINEPTAAALAYGLDKNLKGERNVLIFDLGG

GTFDVSILTIDEGSLFEVRSTAGDTHLGGEDFDNRLVNHLAEEFKRKFKK

DLRSNPRALRRLRTAAERAKRTLSSSTEASIEIDALFEGHDFYTKVSRARFE

ELCGDLFRNTLQPVEKALNDAKMDKGQIHDIVLVGGSTRIPKVQNLLQG

FFGGKTLNLSINPDEAVAYGAAIQAAILSGDESSQIKDVLLVDVAPLSLGIE

TAGGVMTKLIERNSRIPCKQSKTFTTYADNQPAVTIQVFEGERALTKDNN

VLGTFNLTGIPPAPRGVPKIDVTFDLDANGILNVTAKEQGTGNARNITIK

NDKGRLSQADIDRMLNEAEKYAEEDERHRQRIAARNQLEGYVFSVKEA

AEQGGDRISQSDKSSVLEKCNEAVKWLDSNTTAEKEEFEHKLEELTKFCS

PIMTKMHQQGAGAGGDGAGANCGQQSRNYNNGGFKGPTVEEVD

>DaCpn60|XP_001962681.1

MDTGKGSEDWSPSMPRGMNLAAGINKFGLDVGQYCVRQSLVTDEESPL

VMIRMFSTYSTTLQRGIRINRHMWGTRFYAKDVKFGPEVRAMMLQGVD

VLADAVAVTMGPKGRNVIIEQSWGSPKITKDGVTVAKSIALKDKFQNIG

AKLVQDVANNTNEEAGDGTTTATVLARAIAKEGFEKISRGANPVEIRRG

VMLAIDSVKDNLRKMSRPVSTPEEICQVATISANGDKSVGNLISEAIKKVG

RDGVITVKDGKTLCDELEVIEGMKFDRGYISPYFINASKGAKAEFQDALLL

FCEKKKIKSAPSIVPALELANAQRKPLIIIAEDVEGEALSTMVVNRLKVGLQ

VCAVKAPGFGDNRKETMEDMAIATGGIVFGDEANLVRLEDVKLSDFGR

VGEVVVTKDDTMLLKGKGQKEQIDKRVENLREAIKESTSTYEKEKMQER

LARLSSGVALLRVGGSSDIEVSEKKDRVNDALNATRAAVEEGIVPGGGTA

LLRCIQKLNDINGANEDQKLGVEIIRRALRMPCLTIAKNAGVDGAMVVA

KVEILDGDYGYDALKGEYGNMIERGIIDPTKVVRTAITDAAGVASLLTTA

EAVVTELPLEEAAAAGAAAGLGALGGMGGMGM

>DaCpn60|XP_001963430.1

MFRLPVSLARTSISRQLAMRGYAKDVKFGPEVRAMMLQGVDVLADAVA

VTMGPKGRNVIIEQSWGSPKITKDGVTVAKSIELKDKFQNIGAKLVQDVA

NNTNEEAGDGTTTATVLARAIAKEGFEKISKGANPVEIRRGVMLAVETV

KDNLKAMSRTVKTPEEIAQVATISANGDQAIGKLISDAMKKVGRDGVIT

VKDGKTLNDELEVIEGMKFNGGYFFPYFINSTKGAKVEFQDALLLLSEKKI

SSVQSIIPALELANAQRKPLVIIAEDIDGEALSTLVVNRLKIGLQVAAVKAP

GFGDNRKSTLTDMAIASGGIVFGDDADLVKLEDVKISDLGQVGEVVVTK

DDTLLLKGKGKKEDVARRVDQLKDQITETTSEYEKEKLQERLARLASGV

ALLRVGGSSEVEVNEKKDRVHDALNATRAAVEEGIVPGGGTALLRCIEK

LDDVKTQNEDQNLGVEIVRRALRMPCMTIAKNAGVDGSMVVAKVETQ

KGDYGYDALKGEYGNLIEKGIIDPTKVVRTAITDASGVASLLTTAEAVVTE

IPKEDAGPGMGGMGGMGGMGGMGGMGGMM

>DaCpn60|XP_001965046.1

MLHRVGLKGYALVRTYAKDIRFGLEARSMLLHGVNVMANAVATTLGPK

GRNVLIEQLLVSPKITKDGITVANNVELKDRRCNLGAQLIRQATNKTNNE

VGDGTTTATILARGMTCRGMQILRQPNINVQLLREGMLSAARVVSDALQ

DMSQTIDTIGEVEAVAKVALNGDESLSGLIGDVILELGESGVILVKDSSTG

QDEVIFQDGVHISSSYASPFFALNMKDGLLEFRDCLLLITLAKIKAVSQILP

ALEMAKAKDQPIVIIAKEFSADVLKALVVNQLQETVQVCAINAPGCVDE

MREDMQDLAICTGATLLEDESWLADLTEDHFGEVEEVFVNAKETHLLRP

LNVDKKLKKRRFSEIAELKDQAFTDEEMERLHQREGRLMGRMAVIHVG

GVSEMEIVERKERLKDCINAVRKAMSDGVLPGGGTAYLRCIPILDELKPA

KTMEQQMGREIVRDALRLPCYTIARNAGVDPDEVTHNVLEGNGNFGYD

AAAGEYGDLMERQIMDPTDVMRHAVVGATGIAALMATTEVLITEEPVK

PRIPKNQVTRDLANLVGM

>DaCpn60|XP_001965381.1

MFRSCVRDAIRSSRFFTRMYSKEVRFGPEVRAMMIRGVDVLADAVAVTM

GPKGRSVIVERPWTSPKITKDGFTVARSIALKDQHMNLGAKLVQDVADN

TNQAAGDGTTTATVLARAIAKEGFNQITMGANPNEIRRGVMMAVDVV

KEMLKAMSKSVESSEEIQQVATISANGDTDIGRLIAEATEKVGAKGTITVK

DGKRLKDELTIIQGLRFDTGYVSPFFVNSTKGSKVEFSNALVLITLKKITAL

SQIVKGLEQSLRERRPLVIIAEDISGEALNALVLNKLRMGLQVCAVKSPSY

GEHRKELIGDISAATGATIFGDDINYSKIENAKLQDMGQVGEAVITKDST

MLLEGKPKTGQLELRIQQIQDELADKQTKPEQKDRLRQRLSALTKGVAV

LHIGGISEVEVSEKKDRVVDALHATRAAIEEGIVPGGGTAFLRCIPHLERM

EVESKDLKKGVEIICNALRMPCQTIAQNAGVDGAMVVAKVMTKSGDYG

YDAMGGQYGRLIEKGIIDPTKVVRTAISDAAGVASLLSTTEVVITDTRNED

LLAKLAGMGGGGGMDGLDMNMGGLDELAALAGMGGMGGMGGMGG

MGGLGGLGGLGDMGDMTGRISASVPKEDDGPTAEEMNEMVKAIPGME

QVEVKDIDAGLM

>DaCpn10|XP_001955477.1

MASAIKKVIPMLDRILIQRFEVKTTTAGGILLPEESVPKEMQGVVVAVGPG

ARNPAGAGHLSVAVKEGDRVLLPKYGGTKVDMDDKHEYVLFRESDILA
KLE

>DaCpn10|XP_001956372.1

MAAAIKKIIPMLDRILIQRAEALTKTKGGIVLPEKSIGKVLEGTVVAVGPG
ARNASTGNHIPIGVKEGDRVLLPEFGGTKVNLEGDEKQELILFRESDILAK
LE

>DasHsp18.1|XP_001957242.1

MALVPATNSTNDWDYWDYRRRLWRDWDLADWDLPYWKRSLSRVGSA
PDLSRVIVGKDGFEANVDVHLFKPYEITVKTTGDTVVVEAKHEKRRDGD
TFVGRHIVKRFVLPRGYYPNDVRSELSSDGILTVRCPPYLTNERSVYVRQV
GPSYLSIKN

>DasHsp21.7|XP_001957989.1

MPDIPFVLNLDSPDSMYYGHDMFPNRLYRRLHSRQHHDLDLHTLGLIAR
MGAHAHHLVAKKHSGELGALGVSGPLNKQGNFEVHLDVGLFEPGEISV
KLVNDCIVVEGKHEEREDEHGHVFRHFVRRYPLSKEFDADAIASTLSEDG
VLNITVPPLVSKEETKERVIPIKHVGPSDLFLNGNGKKEAAAPAPDSK

>DasHsp19.8|XP_001957992.1

MRSLPMFLRMAEEMTRMPRISSPFHAFFHEPPVWSVALPRNWQQIARW
QEQEFAPPATVSKEGYKLTLDVKDYNELNVKVLDESVVLVEGKSEQKDD
EHGGFSSRHFLRRFVLPEGYEADKVTSSLSSDGVLTINVPNPPAVQEALKE
RVVPIEKTGEPAKKLEAESNSEATKK

>DasHsp23.6|XP_001957993.1

MSLVPTTYRDLSRELDRPRPIYQPPYDFQLYPYLWDDPRVWWPAHTSRSD

MMRPLDELVTRRVRNQLIQSTPYDWSYPMRWDNYYSGERVHVDEKGFR

IDIDVRQFHPHEIVVKTNDDYIIVQGNHNRRSEGSNGLVERHFVRKYLLP

RGYNANEVISDISSDGVLTIKAPPPPPAKYYAPGERLVRVHETGKLALPW

K

>DasHsp22.7|XP_001957994.1

MSLSTLLSLVEDLQEPRNSIYDWGVSPNIRYLQAHTPNRRSINGCPCASPI

CPSSPAGQVMALRREMNNHNNIHWPAVKQVGKDGFQVCMDVAQFKP

SELNVKVVDNSILVEGKHEERQDDHGHIMRHFVRRYTVPEGYEADKVVS

QLSSDGVLTVSIPKPQAIEDKSKERIIQIQQVGPAHLNIKANASEQKGSEN

GTSNGSAK

>DasHsp21.1|XP_001957996.1

MATLPLLLSLADDLGRMSMVPFYEPYYCQRQRSPYLALAGPLEHQLRQL

EKQVGAAGGAKAIVPKVGKDGFQVCMDVSHFKPSELVVKVQDNSILVE

GKHEEREDDHGYITRHFVRRYALPEGYEADKVASTLSSDGVLTVNVPKPP

AIEDKASERIVQIQQVGPAHLNVKQNTKEQLEQGQEQDNGNEK

>DasHsp24.5|XP_001957997.1

MSIIPLLNLARELDHDYRGAFNNDWDHFLEDDFGFGVHAHDLFHRPRL

MMPHGSIGRRRFLPYERNHHHGHHQLVPRRQSGGQNSLLPAVGKDGFQ

VCMDVSQFKPNELTVKVVDKTVVVEGKHEEREDGHGLIQRHFVRKYTLP

KDFDPNEVVSTVSSDGVLTLKAPPPPSKEQAKPERIVQIQQTGPAHLSVK

APEAAAAADGKAENGAGEKMETGK

>DasHsp21.9|XP_001963454.1

MAEANKRNIPIKLGDFSVIDTEFSNIRERFDSEMRKMEEEMAKFRHELMN

REANFFESTSSTKKTTTTTSSTTNSALPSRIPKQQNYVSDISSPLIQDEGDNK

VLKLRFDVSQYAPEEIVVKTVDQKLLVHAKHEEKSDTKSVYREYNREFLL

PKGVNPESIRSSLSKDGVLTVDAPLPALTAGETLIPIAHK

>DasHsp24.4|XP_001963037.1

MPIHWDWDWEHEHEHVGAHYHWPPRRHWSTGEPKCRQRRYYLSHEL

DVCARDFHLRMDDSAWCHGSCLVGRVVIETGTEPDSLGRGTFKVVLDV

HHFQIGELTVKAKNSDTVCVEGKQADDRAEKGQLCITREFTRSYKLPRH

YDATQARATFSADGILMITVPAPPKLDDVERVVEIEPTGNYFGSVADPSA

AKAIEQAELEGGGEDPAGTAMEK

Plutella xylostella

>PxHsp90|Px010238.1

MPEEMQAQSGEVETFAFQAEIAQLMSLIINTFYSNKEIFLRELISNSSDALD

KIRYESLTDPSKLDSGKELYIKIIPNKAEGTLTIIDTGIGMTKADLVNNLGTI

AKSGTKAFMEALQAGADISMIGQFGVGFYSCYLVADRVTVTSKHNDDE

QYMWESAAGGSFTIRSDASEPLGRGTKIVLHIKEDLTEYLEEHKIKEIVKK

HSQFIGYPIKLMVEKEREKELSDDEAEEEKKEEGEDDKPKIEDVGEDEDED

AKDKKKKKKTIKEKYTEDEELNKTKPIWTRNADDITQEEYGDFYKSLTND

WEDHLAVKHFSVEGQLEFRALLFVPRRAPFDLFENKKRKNNIKLYVRRV

FIMDNCEDLIPEYLNFIKGVVDSEDLPLNISREMLQQNKILKVIRKNLVKK

CLELFEELAEDKENYKKYYEQFSKNLKLGIHEDSQNRNKLADLLRFHTSA

SGDEACSFKEYVSRMKENQKHIYYITGENRDQVSNSSFVERVKKRGYEVV

YMTEPIDEYVVQQMREYDGKTLVSVTKEGLELPEDEEEKKKREEDKVKFE

NLCKVMKNILDNKVEKVVVSNRLVESPCCIVTAQYGWSANMERIMKAQ

ALRDTSTMGYMAAKKHLEINPDHSIVETLRQKAEVDKNDKAVKDLVILL

YETALLSSGFSLDEPQVHASRIYRMIKLGLGIDEDEPIQVEEASAGDVPPLE

GDGDDASRMEEVD

>PxHsp70|Px012982.1

MRAKMRWSLVTLVCVLAAVCADEKKEKDKDIGTVIGIDLGTTYSCVGVY

KNGRVEIIANDQGNRITPSYVAFTADGERLIGDAAKNQLTTNPENTVFD

AKRLIGREWSDATVQHDVKFFPFDVVEKNSKPHVSVQTAQGKKVFAPEE

ISAMVLTKMKETAEAYLGKKVTHAVVTVPAYFNDAQRQATKDAGVIAG

LNVMRIINEPTAAAIAYGLDKKEGEKNVLVFDLGGGTFDVSLLTIDNGVF

EVVATNGDTHLGGEDFDQRVMEHFIKLYKKKKGKDIRKDNRAVQKLRR

EVEKAKRALSSSHQVKIEIESFFEGDDFSETLTRAKFEELNMDLFRSTLKPV

QKVLEDADMNKKDVDEIVLVGGSTRIPKVQQLVKEFFNGKEPSRGINPD

EAVAYGAAVQAGVLSGEQDTDAIVLLDVNPLTMGIETVGGVMTKLIPRN

TVIPTKKSQIFSTASDNQHTVTIQVYEGERPMTKDNHLLGKFDLTGIPPAP

RGIPQIEVTFEIDANGILQVSAEDKGTGNREKIVITNDQNRLTPEDIERMIK

DAEKFADDDKKLKERVEARNELESYTYSIKNQLQDKEKLGAKLSDDEKT

KMEEAIDAAIKWLEDNQDVDAEEYKKQKKSVEDVVQPIIAKLYQGQPG

APPPAGEPDDDFKDEL

>PxHsp70|Px002762.1

MRAKMRWSLVTLVCVLAAVCADEKKEKDKDIGTVIGIDLGTTYSCVGVY

KNGRVEIIANDQGNRITPSYVAFTADGERLIGDAAKNQLTTNPENTVFD

AKRLIGREWSDATVQHDVKFFPFDVVEKNSKPHVSVQTAQGKKVFAPEE

ISAMVLTKMKETAEAYLGKKVTHAVVTVPAYFNDAQRQATKDAGVIAG

LNVMRIINEPTAAAIAYGLDKKEGEKNVLVFDLGGGTFDVSLLTIDNGVF

EVVATNGDTHLGGEDFDQRVMEHFIKLYKKKKGKDIRKDNRAVQKLRR

EVEKAKRALSSSHQVKIEIESFFEGDDFSETLTRAKFEELNMDLFRSTLKPV

QKVLEDADMNKKDVDEIVLVGGSTRIPKVQQLVKEFFNGKEPSRGINPD

EAVAYGAAVQAGVLSGEQDTDAIVLLDVNPLTMGIETVGGVMTKLIPRN

TVIPTKKSQIFSTASDNQHTVTIQVYEGERPMTKDNHLLGKFDLTGIPPAP

RGIPQIEVTFEIDANGILQVSAEDKGTGNREKIVITNDQNRLTPEDIERMIK

DAEKFADDDKKLKERVEARNELESYTYSIKNQLQDKEKLGAKLSDDEKT

KMEEAIDAAIKWLEDNQDVDAEEYKKQKKSVEDVVQPIIAKLYQGQPG

APPPAGEPDDDFKDEL

>PxHsp70|Px009079.1

MLCATRSFGRKALECSGVTSDLYTQRNFSTLLRNTASPTVPIYRHGLQSRH

KSEGVRGAVIGIDLGTTNSCVAVMEGKTPKVVENSEGSRTTPSHVAFSKE

GERLVGMPAKRQAVTNSGNTFYATKRLIGRRFDDPEVQKDLKNLSYKVV

KASNGDAWVQGTDGKMYSPSQIGAFVLIKMKETAEAYLNTTVKNAVVT

VPAYFNDSQRQATKDAGQISGLNVLRVINEPTAAALAYGMDKTDDKIIA

VYDLGGGTFDISVLEIQKGVFEVKSTNGDTLLGGEDFDNVLVNFLVDEFK

KDQGIDLRKDAMAMQRLKEAAEKAKIELSGSVQTDINLPYLTMDAAGP

KHMNLKMTRSKLESLVGDLIKRTVAPCQRALQDAEVTRTDIGEVLLVGG

MTRMPKVQSTVQEIFGRAPSRAVNPDEAVAVGAAVQGGVLAGDVTDIL

LLDVTPLSLGIETLGGVFTKLITRNTTIPTKKSQVFSTAADGQTQVEIKVHQ

GEREMAADNKMLGQFSLVGIPPVEVTFDIDANGIVHVSARDKGTGKEQQ

IVIQSSGGLSKDEIENMVKAAEKFAAADKQRRERVEVSNQAEGVLHDTE

TKMDEYKAQLPQEECDKLKTEITALRDLLAKKDEADPEDIRKATGALQQ

ASLKLFEQASLKLFEQAYKKMAAEREGNNQQQTETPPSEDDKKEEKKN

>PxHsp70|Px003972.1

MPAVGIDLGTTYSCVGVWQHGNVEIIANDQGNRTTPSYVAFTDTERLIG

DAAKNQVALNPNNTVFDAKRLIGRKFDDPKIQADMKHWPFKVVSDCG

KPKIQLEYKGETKRFAPEEISSMVLTKMKEIAEAYLGTSVRDAVVTVPAYF

NDSQRQATKDAGAIAGLNVLRIINEPTAAALAYGLDKNLKGERNVLIFD

LGGGTFDVSILSIDEGSLFEVKSTAGDTHLGGEDFDNRLVNHLVQEFKRK

YHKDLTGNARALRRLRTAAERAKRTLSSSEATIEIDALYDGIDYYTRVSR

ARFEELNADLFRGTLEPVEKALKDAKLDKSQIDDVVLVGGSTRIPKIQTM

LQNFFCGKKLNLSINPDEAVAYGAAVQAAILTGNTDTRIQDVLLVDVAP

LSLGIETAGGVMTKIIERNSKIPCKQSQTFTTYADNQPAVTIQVFEGERALT

KDNNLLGTFDLTGIPPAPRGVPKIDVTFDMDANGILNVSAKDNSSGRSK

NIVIKNDRGRLSQAEIDRMLSDAEKYKDEDEKQRKRIAARNQLEAYIFSV

KQAVEESGDKLSDDDKGTARRECEEALKWLDNNNLADQEEYEHKLKEL

QRVCSPIMTRMHGGGNACNGGGMPGGMPGGGMGGMPGGMGGMPG

GMGGMPGGMGGMPGDYGNNSGPTIEEVD

>PxHsp70|Px007715.1

MPAIGIDLGTTYSCVGVWQHGNVEIIANDQGNRTTPSYVAFTDSERLIGD

AAKNQVALNPNNTVFDAKRLIGRKFDDPKIQADMKHWPFRVVSDCGK

PKIQVEFKGESKTFAPEEISSMVLTKMKETAEAYLGTAVRDAVVTVPAYF

NDSQRQATKDAGAIAGLNVLRIINEPTAAALAYGLDKNLKGERNVLIFD

LGGGTFDVSILTIDEGSLFEVKATAGDTHLGGEDFDNRLVNHLAEEFKRK

YKKDIRANSRALRRLRTAAERAKRTLSSSSEATIEIDALYEGVDFYTRVSRA

RFEELNSDLFRGTLEPVEKALRDAKLDKSSIHDVVLVGGSTRIPKIQSMLQ

SFFNGKQLNLSINPDEAVAYGAAVQAAILSGEQHSKIQDVLLVDVAPLSL

GIETAGGVMTKIIERNAKIPCKQSQTFTTYADNQPAVTIQVYEGERAMTK

DNNLLGRFDLTGIPPAPRGVPKVDVTFDLDANGILNVGRTKNIVIKNDK

GRLSQAEIERMLAEAERYRDEDERQRERVAARNQLETYIFGVKQALDDA

GDRLSEQDKQAARGRCDDALRWLDNNTLAERDDKLTARTDVKIALAAS

AALSAWYVRNSFKTKGKSSDGQLSTLSPEERIQYMIKEKNKKGPKAQVD

ARFFAELKALWKIMVPGLWTKEAGYMALIAFSLISRSMCDLWLIQHTTLI

EGSIISMNKKEFVRLLTQFFTAMPLISLVNNILKWSIGEMKLNIRTNLTMH

LYENYMKGFTYYQVTNLDNRISNADQLLTTDVEKFCETVVDLYSNISKPM

LDIGIYLYRLTMNLGATKFCETVVDLYSNISKPMLDIGIYLYRLTMNLGGT

TPGIMMAYLFLSGVLLTHLRKPTARLTVEEQKLEGEFRYVNSRLITNSEEIA

FYQGNHREQLTLLASFYKLTRHLRRFLNFRVGMGFVDNIIAKYIAITVGFF

AVSRPFFVKDHNLLTTGSDNDRFKHYYTYGRMLVKMAEAIGRLVLSGRE

LTKLAGLTSRVTQLRVVLDEVNKGKYTRTMLDNSTANGHGNGPPMILA

PGAGRIIYQDNIIKFDKVPLVTPNGDVLIKELCFEVKSGINVLVCGPNGCG

KSSMFRTLGELWPVFGGALTKPPRGKLFYVPQRPYMTLGTFRDQIIYPQTR

EEMLRRGRSDTELTKFLDIVQLSYLSQRDGGWDAVEDWMDVLSGGEKQ

RVAMARLFYHEPQFAILDECTSAVSVDVEGQMYQYCREMGISLFTVSHR

KSLWKHHDHFLQMDGLGGYVFGEIDGETQEFGS

>PxHsp70|Px010237.1

MPAIGIDLGTTYSCVGVWQHGNVEIIANDQGNRTTPSYVAFTDSERLIGD

AAKNQVALNPSNTVFDAKRLIGRKFDDPKIQADMKHWPFKVISDGGKP

KIQVEFKGEVKKFAPEEVSSMVLTKMRETAEAYLGTSVKDAVITVPAYFN

DSQRQATKDAGAIAGLNVLRIINEPTAAALAYGLDKNLKGERNVLIFDL

GGGTFDVSVLTIDEGALFEVRSTAGDTHLGGEDFDNRLVNHLADEFKRK

YKKDLRQSPRALRRLRTAAERAKRTLSSSTEATIEVDALYDGEDFYTRVSR

ARFEELNADLFRGTLEPVEKALKDAKLDKSQIHDVVLVGGSTRIPKIQSLL

QNLFCGKKLNLSINPDEAVAYGAAVQAAILSGSTDARIQDVLLVDVAPLS

LGIETAGGVMTKIIERNSKIPCKQSQTFTTYSDNQPAVTIQVYEGERAMTK

DNNLLGTFDLTGIPPAPRGVPKIDVTFDMDANGILNVSAKENSSGRTKNI

VIKNDKGRLSQAEIDRMLADAERYRDEDDKQRQRVAARNQLESYVFSVR

QALDDAGDKLSDSDKTAARKECEDALKWLDNNTLAEQDEYEHRLKEV

QRVCSPIMSKMHGAGAAGMNPGPQGQGPTVEEVD

>PxsHsp21.6|Px005794.1

MADSGLKRNIPIKLGDFSVIDTEFSSIRERFDAEMRKMEEEMSKFRSDLMN

RESNNFFKSTTSSTTSSQHSDSRQLAEPANTHWDSLNSPLIQDEGDGKSLK

LRFDVSQYTPEEIVVKTVDNKLLVHAKHEEKSDTKSVYREYNREFLLPKG

TNPEAIKSSLSRDGVLTVEAPLPQLAITDRHIPIQKH

>PxsHsp20.1|Px003977.1

MSLLPFLFDYEIERPRRLMDQHFGLALTPQDFLAMAAPAPQLSRDYYRP

WRHMAAALRDVGSNIKADKDKFQVNLDVQHFAPEEISVKTADGYIIVEG

KHEEKQDDHGFVSRQFTRRYALPEGCQAETVESRLSSDGVLTIVAPRKVP

DAVKGERAVPIAQTGPVRKEIKDQTNGAEK

>PxsHsp19.9|Px003978.1

MSLLPYVLGYNYPRRLLDQDFGLALTPDDLLPVVAAPMMSADYYRPWR

QRAAAARDVGSTLKADKDKFQVTLDVQHFSPDEIAVKTADGYVVGEGR

HEEKKDEHGYICRQFSRRYALPEGCKPEAVEARLSSDGVLTIGAPRKCPD

AIKGERAVPIAQTGPVRKEITDNTNEKSAQNGSK

>PxsHsp19.9|Px003979.1

MSLLPYVLGYNCPRRLLDQDFGLALTPDDLLTVVAAPMMSADYYRPGR

QLAAAARDVGSAIKADKDKFQVNLDVQPFSPDEISVKTADGYVVGEGR

HEEKKDEHGYLSRQFSRRYALPEGCKPEAVESRLSSDGVLTIVAPRTCPDA

IKGERAVPIAQTGPVRKEITDNTNEKASGDKKKK

>PxsHsp23.5|Px003980.1

MGDFGRKTFLLAAAVTFLIISPRHIMAEETCAGKEKTDKAETAANTLYDQ

DFGMALTPEDLLTTMLSPFSSPWMMRDYFRPWRLSAVNDLGSTIKTDKD

KFQVNLDVQHFTPDEITVKTDDGFIIVEGKHEEKKDDHGMVARQFVRKY

SLPRGTRPETVSSSISADGVLTVTAPLDPSAAVGLRVVPIEKTGPVRKVKK

AEGDGVDGSCNGQTG

>PxsHsp27.5|Px005412.2

MLSSRTLLTLALGLAVAAAVPVDKSTIDVSDKPALRPITTIEADDGDEGT

WFSFPLFGNFGSFGNLGSLFTPIWKLFPNFADLGPRINSDENKFDIIVNVKE

YKKDELKVKVKGFLIIVQGVHEAKTDSDIFDRQFFHTYTLPVNASGTDVT

ADLFNNGFLVVSCPLNGKSDKDEEVNRDVEIKETGTAYELQDKTTVGSS

QPEGTKAPVEVSTAGLVENEDDRKEPTTPSSERELAKDNIIPHGQTNEALP

>PxsHsp22.1|Px010239.1

MALLPYLLDYDIRPRRTRDFFGLGGFDLEDLLLDRPQLRPRRRLGHPALRS

LSSLTLPDVGSTIKADKDKFQVNLDVQHFAPEEITVKTVDGCVVVEGKHE

EKEDDHGYVSRSFKRRYTLPEDCNPEAVESRLSTDGVLTILAPKASTGKGE

RAVPITHTGPVRRKQVEDQEVEDQANGDAEEDVKTPQQKRRKR

>PxsHsp14.4|Px014444.1

YYRPWRHLAAAARDLGSNIKADKDQFQVNLDVQHFSPEEISVKTADGFI

VIEGKHEEKKDQHGFVSRQFTRRYSLPEGCDPDAVESKLSSDGVLTVVAP

RKPQAVQGERSVPIAHTGPVRKEVNANGDK

>PxsHsp19.7|Px014445.1

MSLLPYFYDYDFQFPRRAFDQSFGHGLAPELLMAASPLRHRLPYWPRLPA

DVGSSIKTDKDSFQVNIDVQHFAPEEITVKTADGFIVVEGKHEERKDEHG

FISRQFVRKFKLPEGCDLEAVQSKLSSDGVLSVVAPKKVEAVKGERSVPIS

HTGPVRKEVKEDLTVEPKDEGKK

>PxsHsp21.6|Px015010.1

MADSGLKRNIPIKLGDFSVIDTEFSSIRERFDAEMRKMEEEMSKFRSDLMN

RESNNFFKSTTSSTTSSQHSDSRQLAEPANTHWDSLNSPLIQDEGDGKSLK

LRFDVSQYTPEEIVVKTVDNKLLVHAKHEEKSDTKSVYREYNREFLLPKG

TNPEAIKSSLSRDGVLTVEAPLPQLAITDRHIPIQKH

>PxsHsp13.0|Px013294.1

MAARPLRPRLPYWPRLPADVGSSIKTDKDSFQVNIDVQHFAPEEITVKTA

DGFIVVEGKHEERKDEHGFISRQFVRKFKLPEGCDLEAVQSKLSSDGVLSV

GAPKKVEAVKGERRG

>PxsHsp21.9|Px011945.2

MSVLPYYDFDRMMQRMEREFFNSPFHQLFPRDLYRPMGDFLKPWDNVF

RPWEDIMKPLDNAFSHLALQDAGSSITADDEKFQVNVDVQHFAPEEVQ

VKVKDREVMIEGRHEEKRDDHGYISRQFIRRYTLPEGCLPDTVVSRLSSDG

VLTVTAPKVLPLPSAGERIIPITHTGPVQKRIGQGATVHEVK

>PxsHsp19.2|Px012965.1

MSLIPYWARHLRNLACRDPVMRVLEDPFTALSRDPFFRDPLKYMKQMSA

LDHDHSHGIDGVLSETEVKADGKKVEVHLDVQNFTPDQIQVKTVGNEIT

VEGKKEVKKEGGWSRSHFERRFLLPEGFPPERVECHLDKGRLVLVAFRAQ

PVEERTVPIQDKTKDAEKS

Nilaparvata lugens

>NlHsp90|BPHOGS10016311-TA

MGLTLVYTDSDPNRQPLGPVLQKHKFVGNAYDKPRFTLHYKTDSPLMIQ

ALLYFPETKPDMFDLSSDKSSQVALYSRRVLIQSKADSLLPKWLRFVKGEK

LRIVLTNKILRFLNDKSTQQPQEYADFYKDYNIFLKEGVITSQDQNEKKEV

AKLLRYESSLKPAGETRHAERHEERLHQHDNVEAIQLLDNGGLVRRLKR

RKPFELV

>NlHsp70|BPHOGS10001502-TA

MPVKTPAIGIDLGTTYSCVGVFQQGKVEIIANDQGNRTTPSYVAFTDSERL

IGDAAKNQVAMNPKNTIFDAKRLIGRRFDDPKITQDMKHWPFSVFNDC

GKPKLQVEFKGEVKKFSPEEISAMVLTKMKETAETYLGAPVKDAVITVPA

YFNDSQRQATKDAGAIAGLNVLRIINEPTAAALAYGLDKNLKGERNVLI

FDLGGGTFDVSILTIDEGSLFEVRSTAGDTHLGEASIEIDALCEGVDFYTKV

SRARFEELCADLFRSTLHPVEKALNDAKMDKSSIHDVVLVGGSTRIPKIQS

LLQNFFNGKSLNLSINPDEAVAYGAAVQAAILSGDQSSQIQDVLLVDVTP

LSLGIETAGGVMAKIIERNCRIPCKQSQTFSTYSDNQPAVTIQVFEGERAM

TKDNNLLGTFDLTGIPPAPRGVPKIEVTFDLDANGILNVSAKETGTGITKN

IVIKNDKGRLSKADIDRMVNEAEQFKEEDEKQRQKVSARNQLENYIFSVK

QALEEAGSKLSDGDK

>NlHsp70|BPHOGS10005135-TA

MPVKAPAIGIDLGTTYSCVGVFQQGKVEIIANDQGNRTTPSYVAFTDSER

LIGDAAKNQVAMNPKNTIFDAKRLIGRRFDDPKITQDMKHWPFNVFND

CGKPKLQVEFKGEVKKFSPEEISAMVLTKMKETAETYLGAPVKDAVITVP

AYFNDSQRQATKDAGAIAGLNVLRIINEPTAAALAYGLDKNLKGERNVL

IFDLGGGTFDVSILSIDEGSLFEVRSTAGDTHLGGEDFDNRLVDHLSEEFKR

KYRKDVKSNPRALRRLRTAAERAKRTLSSSTEASIEIDALCEGVDFYTKVS

RARFEELCADLFRSTLHPVEKALNDAKMDKSSIHDVVLVGGSTRIPKIQSL

LQNFFNGKSLNLSINPDEAVAYGAAVQAAILSGDQSSQIQDVLLVDVTPL

SLGIETAGGVMTKIIERNCRIPCKQSQTFTTYADNQPAVTIQVFEGERAMT

KDNNLLGTFDLTGIPPAPRGVPQIDVTFDVSAKENGTGITKNIVIKNDKG

RLSKTDIDRMVNEAEQFKEEDEKQRQKVSARNQLENYIFSVKQALDEAG

SKLSDGDKSAARQRCDECLQWLDNNTLAEKEEFEHKLKELQQLCSPIMA

KIYSSQGGGDGRRSQTCGQQFNQGQSFNNNGPTIEEVD

>NlHsp70|BPHOGS10006669-TA

MPGGSPTIGIDLGTTYSCAGAFLMGEVRILVNDQGNKTTPSYVAFTDHAR

FIGEAAKNYVTRNPQNTVFDAKRLIGRRYDDPKITQGMRHWQFGVLND

CGKPMIEVDFKGAKKRFYPEEISAMILAKMKEIAENNLGTAVNNAVITVP

AYFNDSQRQATKDAGTIAGFNVLRIINEPTAAALAYGLDKHVKGERNVL

IFDLGGGTFDVSILTIDECSVFEVQSTAGNTYLGGEDFDYRLVDYLCEEFRR

KHGMDLRTNRRAMARLKTAAERAKRSLSSSTEASIEIDSLIEGIDFFTRVSR

TRFEELCADLFLSTLLPVERALECARMDKSEIHDVVLVGGSTRIPKIQSLLQ

NFFNGKPLNVAINPDEAVAYGAAIYAAILTGDNGAFIKDILLVDVTPLSL

GIETGVDIMATVIERNTQIPCKRSRIFTTHEDNQPDVEIQIYEGERVLAKDN

NLLGTFDLIGIPPAPRGVPQIKVTFDINADGILNVEAEDIGTGFSESITIKNN

KGRLSPAQINRMLAEAEQFKKEDEKLRQRVSIMNQMESYIFSTKQAVNE

AGNKITEIEKIETLQCCDEYLQWLEGKPTAEKDELQLKFNELQIQCAVVL

VKIHQDDPNGKFINIDHLKC

>NlHsp70|BPHOGS10007039-TA

MGKPQPAIGIDLGTTYSCVGVWQHGKVEIIANDQGNRTTPSYVAFTDTE

RLIGDAAKSQVAMNPKNTVFDAKRLIGRKFDDPKIQDDMKHWPFAVVD

HSGKPKIRVEYKGEQKTFAPEEISSMVLTKMKEISETYLGTKVKDAVITVP

AYFNDSQRQATKDAGAIAGLNVLRIINEPTAAALAYGLDKNLKGEKNVL

IFDLGGGTFDVSILAIDEGSLFQVKSTAGDTHLGGEDFDNRLVKHFADEF

QRKNKKDLTSNVRALRRLRTACERAKRTLSSSTEASLEIDALHDGIDFYA

KITRARFEEMCMDMFRSTLAPVERALTDAKLDKGAIHDVVLVGGSTRIPK

IQKMLQDFFCGKALNLSINPDEAVAYGAAVQAAILSGDTSSAIQDVLLVD

VAPLSLGIETAGGVMTKLVERNSRIPCKQQQTFTTYSDNQSAVTIQVFEGE

RAMTKDNNLLGTFNLTGIPPAPRGIPKIEVTFDLDANGILNVSAKDSSTG

KSEKITIQNDKGRLSKEEIDRMLKEADKYKAEDDKLRERVAARNQLEGY

AFSVKQAAEDAGAKLSEAEKKQVQDKCAAVLAWLETNTQAEKDEFED

KLKELQKECTPIMTKIHQGAGGGAGANAKGPTVEEVD

>NlHsp70|BPHOGS10007752-TA

MPVKTPAIGIDLGTTYSCVGVFQQGKVEIIANDQGNRTTPSYVAFTDSERL

IGDAAKNQVAMNPKNTIFDAKRLIGRRFDDPKITQDMKHWPFTVYSDC

GKPKIQVDFKGEVKKFSPEEISAMVLTKMKETAETYLGAPVRDAVITVPA

YFNDSQRQATKDAGAIAGLNVLRIINEPTAAALAYGLDKNLKGERNVLI

FDLGGGTFDVSILTIDEGSLFEVRSTAGDTHLGGEDFDNRLVDHLSEEFKR

KYRKDIKSNPRALRRLRTAAERAKRTLSSSTEASIEIDALCEGVDFYTKVSR

ARFEELCADLFRSTLHPVEKALNDAKMDKSSIHDVVLVGGSTRIPKIQSLL

QNFFNGKSLNLSINPDEAVAYGAAVQAAILSGDQSSQIQDVLLVDVTPLS

LGIETAGGVMAKIIERNCRIPCKQSQTFSTYSDNQPAVTIQVFEGERAMTK

DNNLLGTFDLTGIPPAPRGVPKIEVTFDLDANGILNVSAKETGTGITKNIVI

KNDKGRLSKADIDRMVNEAEQFKEEDEKQRQKVSARNQLENYIFSVKQ

ALEEAGSKLSDGDKSAARQRCDECLQWLDNNTLAEKEEFEHKLKELQQ

LCSPIMAKIYSSGAANGPTSCGQQFNQSQSFPRSGPTVEEVD

>NlHsp70|BPHOGS10010728-TA

MPVKTPAIGIDLGTTYSCVGVFQQGKVEIIANDQGNRTTPSYVAFTDSERL

IGDAAKNQVAMNPKNTIFDAKRLIGRRRLSKADIDRMVNEAEQFKEEDE

KQRQKVSARNQLENYIFSVKQALEEAGSKLSDGDKSAARQRCDECLQWL

DNNTLAEKEEFEHKLKELQQLCSPIMAKIYSSGAANGPTSCGQQFNQGQ

SFPRSGPTVEEVD

>NlHsp70|BPHOGS10017149-TA

MPLAIGIDLGTTYSCVGVWQQGKVEVIANDQGNRVTPSYVAFTETERLIG

DAAKNQVAMNPENTVFDAKRLIGRRYDDPKIAEDLKHWPFKVISESGKP

KIQVQYKGVDKTFTPEEISSMGKHPQHTFIFFKLISLFYSFIDQNNKVNMPL

AIGIDLGTTYSCVGVWQQGKVEVIANDQGNRVTPSYVAFTETERLIGDAA

KNQVAMNPENTVFDAKRLIGRRYDDPKIAEDLKHWPFKVISESGKPKIQ

VQYKGVDKTFTPEEISSMVLTKMKATAEAFLGRKISDAVITVPAYFNDSQ

RQATKDAGAIAGLNVLRIINEPTAAALAYGLDKNLSGEKKVLIFDLGGGT

FDVSILTIDEGSLFEVKATAGDTHLGGEDFDNRLVDHFSNEVKKKYGKDL

RKNARALRRLRTAAERAKRTLSSSTEATLEIDALIDGVDYHTRISRARFEEL

CSDLFKKTLAPVEKALQDAEMGKSDIDDVVLVGGSTRIPKVQSLLQNFFN

GKTLNLSINPDEAVAYGAAVQAAILSGDRSSEIQDVLLVDVTPLSLGIETA

GGVMSTIIERNTRIPGKKSQTFTTYSDNQSAVTIQVFEGERAMTVDNNLL

GTFDLTGIPPAPRGVPHIDVTFDLDANGILNVSAKENSTGKTRNIVIKNDK

GRLSKEEIDRMVRDAEKYKAEDDRQRKRVAARNRLESYALSVKQALAEA

GSRLTPADISLAGDKCEAALEWLENNPQATCDECDVRMSELQRVCSPIM

TKLHRGRGDSNEGPTIEEAD

>NlHsp70|BPHOGS10021590-TA

MAAMSVIGIDFGNQSSYVAVARAGGIETIANDYSLRATPSCVAFSDKSRIL

GVAAKNQQITNLDNTISGFKRLLGRKYNDPAVQAELRELSFKTVQQKNG

GIGIKANFQGETHVFSPEQITAMFFTKLKDTSEIALKTKVNDCVISVSSVIL

HGRRTQSTVGRGIHSRAERAATDERDDGHRTLVRHLQRGLAAARGEAA

QRDLRRLRLTXXXXXXXXXXGASPPRRVSAGAFHKGKLKMLACASDPNF

GGRDIDLILAEEFNKNFQQKFKVDARSNPRAFLRLMNEVEKFKKQMSAN

STKLSLFIDCFVDDKDVSGDMKRADMETLCSHLLQRVEKTLKQCLDDSN

SWRLDAYSGDQAADHERVREDANTTLNQDEAVARGCALQCAMLSPAV

RVREFNVTDIQTYPIKLVWDAAMGENGEMEVFSLHHAVPFSKMLTFYRK

EPFSIKAYYSGQIPYPDPYIGEFVVKEVKPTSTGESAKVKVKVRVNVNGVL

TVTSARREAPAEAGDQQEEAAGQPDAPMDASPKQDAPAPASVPDNEN

NTEEEQEKKEEKKSGKKITITELPIESFTNGLSVVEINNLLEMECKMIASDK

QEKERVDTRNALEEYIYELRGQLNEEPVSDPNGRLSLCQFVKEADHQSLI

KQLDGLETWLYEDGDECTRQIYAEKLSSLKNIGEPIKMRQQEREQRPIAF

NEFVASIQQALGVTEQFRAGSKLYNHLVEADVAKVEQSVNTARKWVED

KLAESKNCPLIENPAVTVAQIRQERLNFELEVSPILKKAKRKPAPAASEAP

NENGQQPTSGSGQADQPAAGDQKQQTEQNSAEKMDVE

>NlHsp70|BPHOGS10023721-TA

MPVKTPAIGIDLGTTYSCVGVFQQGKVEIIANDQGNRTTPSYVAFTDSERL

IGDAAKNQVAMNPKNTIFDAKRLIGRRFDDPKITQDMKHWPFSVFNDC

GKPKLQVEFKGEVKKFSPEEISAMVLTKMKETAETYLGAPVKDAVITVPA

YFNDSQRQATKDAGAIAGLNVLRIINEPTAAALAYGLDKNLKGERNVLI

FDLGGGTFDVSILTIDEGSLFEVRSTAGDTHLGGEDFDNRLVDHLSEEFKR

KYRKDIKSNPRALRRLRTAAERAKRTLSSSTEASIEIDALCEGVDFYTKVSR

ARFEELCADLFRSTLHPVEKALNDAKMDKSSIHDVVLVGGSTRIPKIQSLL

QNFFNGKSLNLSINPDEAVAYGAAVQAAILSGDQSSQIQDVLLVDVTPLS

LGIETAGGVMAKIIERNCRIPCKQSQTFSTYSDNQPAVTIQVFEGERAMTK

DNNLLGTFDLTGIPPAPRGVPKIEVTFDLDANGILNVSAKETGTGITKNIVI

KNDKGRLSKADIDRMVNEAEQFKEEDEKQKHKVSARNQLENYIFSVKQ

ALEEAGSKLSDGDKSAARQRCDECLQWLDNNTLAEKEEFEHKLKELQQ

LCSPIMAKIYSSGAANGPTSCGQQFNQGQSFPRSGPTVEEVD

>NlsHsp15.9|BPHOGS10024450-TA

MDKLFSRITGEKPLSDVPSYNLYQKDKENLELTVAVPGYKQHELDISVLN

NKLTISGKSESEKEEESNDSKTEKWLHKGIEKKGFSMSFDLDHRIRIQNAN

LADGLLTLTFNYEIPEEQKPQKIAIGQTSQPEVIEHKK

>NlsHsp16|BPHOGS10024467-TA

MDKLFSRITGEKPLSDVPSYNLYQKDKENLELTVAVPGYKQHELDISVLN

NKLTISEKSESEKEEESNDSKTEKWLHKGIEKKGFSMSFDLDHRIRIQNAN

LADGLLTLTFNYEIPEEQKPQKIAIGQTSQPEVIEHKK

>NlCpn60|BPHOGS10000283-TA

MFRLPLALRSTAIRQLKRNYAKDVRFGPEVRGLMLQGVDILADAVAVTM

GPKGRNVILEQSWGSPKITKDGVTVAKGVELKDKFQNIGARLVQDVAN

NTNEEAGDGTTTATVLARAIAKEGFDKISKGANPIEIRRGVMLAVDAIKE

HLKTLSKPVTTPEEIAQVATISANGDKAIGELISAAMKKVGKEGVITVKDG

KTLEDELEVIEGMKFDRGYISPYFINTAKGAKVEFQDALLLLSEKKISSIQAI

IPALELANSQRKPLVIIAEDVDGEALSTLVVNRLKIGLQVAAVKAPGFGD

NRKNTLQDMAIATGGIVFGDEGANLKLEDITPTDLGQVGEVVITKDDTL

LLKGKGKKSDIDRRAELLRDQIADTTSEYEREKLQERLARLASGVAVLKV

GGSSEVEVNEKKDRVTDALNATRAAVEEGIVPGGGTALLRCAAKLKQV

VTANADQATGIDIVQKALRMPCMTIAQNAGVDASVVVSKVAEGTGDM

GYDALNNEYVNMIEKGIIDPTKVSNYKCYSLKNHQCICTN

>NlCpn60|BPHOGS10009278-TA

MYRLPLALRSTAIRHLNRNYAKDVRFGPEVRGLMLQGVDILADAVAVT

MGPKGRNVILEQSWGSPKITKDGVTVAKGVELKDKFQNIGAKLVQDVA

NNTNEEAGDGTTTATVLARAIAKEGFDKISKGANPIEIRRGVMLAVDAIK

EHLKALSKPVTTPEEIAQVATISANGDKAIGELISAAMKKVGKEGVITVKD

GKTLEDELEVIEGMKFDRGYISPYFINTSKGAKVEFQDALVLLSEKKISSIQS

IIPALELANSQRKPLVIIAEDVDGEALSTLVVNRLKIGLQVAAVKAPGFGD

NRKSTLQDMAIATGGIVFGDEGANLKLEDLTPTDLGQVGEVLITKDDTLL

LKGKGKKSDIDRRAEQLRDQIQETTSEYEREKLQERLARLASGVAVLKVG

GSSEVEVNEKKDRVTDALNATRAAVEEGIVPGGGTALLRCAAILKQVITA

NADQATGIDIVQKALRMPCMTIAQDAGGDASVIVSKVAEGTGDMGYDA

LNNEYVNMIEKGIIDPTKVRPHSIIKLNLM

>NlCpn60|BPHOGS10035223-TA

MFRLPLALRSTAIRQLKRNYAKDVRFGPEVRGLMLQGVDILADAVAVTM

GPKGRNVILEQSWGSPKITKDGVTVAKGVELKDKFQNIGARLVQDVAN

NTNEEAGDGTTTATVLARAIAKEGFDKISKGANPIEIRRGVMLAVDAIKE

HLKTLSKPVTTPEEIAQVATISANGDKAIGELISAAMKKVGKEGVITVKDG

KTLEDELEVIEGMKFDRGYISPYFINTAKGAKVEFQDALLLLSEKKISSIQAI

IPALELANSQRKPLVIIAEDVDGEALSTLVVNRLKIGLQVAAVKAPGFGD

NRKNTLQDMAIATGGIVFGDEGANLKLEDITPTDLGQVGEVVITKDDTL

LLKGKGKKSDIDRRAELLRDQIADTTSEYEREKLQERLARLASGVAVLKV

GGSSEVEVNEKKDRVTDALNATRAAVEEGIVPGGGTALLRCAAKLKQV

VTANADQATGIDIVQKALRMPCMTIAQNAGVDASVVVSKVAEGTGDM

GYDALNNEYVNMIEKGIIDPTKVSNYKCYLLKIYPCICTN

>NlCpn10|BPHOGS10034737-TA

LITTSNLLLTLTTHLCRRRCLSDSCRPLSAPIETGGCLFSRVSPSSLSFSLSLSP

SLSLLIALFLPRLRAKRRTMVSAIRSIRNLTPLLDRVLVQRVKAQTKTAGGI

ILPEASMEKLNEAKVLAVGPGALDKDGKRIPMSVTTGDHVLIPQYGGAP

VKAGDEEYQLFRDSESRIPREAKKATRSGYVCTIRQEPVGDEACGQSPRW

LKEEGRNRGLCMRACCVGNGSLTGRWPDKDTGLASRGTC

>NlCpn10|BPHOGS10035224-TA

MAFAKQAIKKLVPMFDRVLVLRADAVTKTTGGILIPEKSQGKVLHGTVIA

VGPGARNNNGELVPTQIKVGDQVLLPEYGGTKVELEENKEYHLFRESDIL

AKLEV

Tribolium castaneum

>TcHsp90|TC011024-PA

MANLGRIALQRVFLSGRRHLTCAPIVFLRPIRKKTGAASFVRCTSTSPNAS

KASDEEHHSIIKDTEQPKGEPTKHEFQAETRMLLDIVARSLYSDKEVFVRE

LISNASDALEKFRYLTVSDREGLRDPNRVLEIHISTDKQARTLTIQDTGIGM

SKEELIANLGVIARSGSKAFIEQLKSKELTSDSSNNIIGQFGVGFYSVFMVA

DRVDVYTRSSMEDQGYKWSSDGTGSYQIQDAEGVQCGTKIVLHLKTDCR

EFCDETTVENVINKYSNFIGSPIYLNGKKANIVQPVWLSEPKSVTVQQHN

EFYRFISGSYDVPRYILHYKTDVPLSIHALLYFPEGKPGLFEMSREVESGVA

LYTRKVLIKNKTDNILPKWLRFVKGVVDSEDIPLNLSRELLQNSALISRSLA

EASPYYESLKSKHQEVLFCYEPYDELVLMQLQQFKGFKLVSVEKDMRDD
KAANDLSNLGEDSLKRSEVNELSEWIKSKLSGKAVDVNATTRLENHPCV
VTVEEMAAARHFIRTQSHQLSEDRRYAILQPKFEINPRHPIIKKLHKLMTS
DPHLAELLANQLFANSMVGAGLVEDPRVLLTSINALLTKALEKH

>TcHsp90|TC014606-PA

MPEENQNGDVETFAFQAEIAQLMSLIINTFYSNKEIFLRELISNSSDALDKI
RYESLTNPSRLDSGKELYIKIIPNKNDGTLTIIDTGIGMTKADLVNNLGTIA
KSGTKAFMEALQAGADISMIGQFGVGFYSAYLVADKVTVVSKNNDDEQ
YVWESSAGGSFTVTQDRGEPLGRGTKIVLHMKEDQTEFLEEHKIKEIVKK
HSQFIGYPIKLVVEKEREKELSDDEAEEEKKEEEGEDKDKDKPKIEDVGED
EDEDTKKEDKKKKKTIKEKYTEDEELNKTKPIWTRNADDISQEEYGEFYK
SLTNDWEDHLAVKHFSVEGQLEFRALLFVPRRVPFDLFENKKRKNNIKL
YVRRVFIMDNCEELIPEYLNFIKGVVDSEDLPLNISREMLQQNKILKVIRK
NLVKKCLELFEELAEDKDGYKKFYEQFSKNIKLGIHEDSQNRAKLSELLRY
HTSASGDEACSLKDYVSRIKPNQKHIYYITGESKEQVANSSFVERVKKRGF
EVVYMTEPIDEYVVQQMKEFDGKTLVSVTKEGLELPEDEEEKKKREEDKA
KFEGLCKVMKSILDNKVEKVVVSNRLVESPCCIVTSQYGWTANMERIMK
AQALRDTSTMGYMAAKKHLEINPDHSIIENLRQKAEADKNDKAVKDLV
ILLFETALLSSGFTLDEPQVHASRIYRMIKLGLGIDEEEAMITEDAQGGDAP
SADAAESEDASRMEEVD

>TcHsp90|TC012185-PA

MKQLIVLAINLLLLIGYTRADESIGEGTVEKVDLDLGASREGSRTDDEVVK

REEEAIKLDGLNVAQLKELRDKAEKFTFQTEVNRMMKLIINSLYRNKEIFL

RELISNASDALDKIRLLSLTDKNVLDSNPELNIRIKADKEAGMLHITDTGI

GMTKQDLVNNLGTIAKSGTAEFLSKMQDASTAQDMNDMIGQFGVGFYS

AFLVADKVVVTTKHNDDKQYIWESDSSSFSIVDDPRGDSLKRGTTVSLQL

KPEAKDFLEHDTVRSLVKKYSQFINFPIYMWTSHTEQVEEPIEEDEKSEEK

EKPDTEDEAAVEEEKEEEKPKTKKVDKTVWDWELLNDSKPIWTKKPSEV

DDKEYDEFYKSLTKDSKQPLAKVHFIAEGEVTFKALLYVPEVQPSESFNRY

GTKTDNIKLYVRRVFITDEFNDMIPSYLSFVRGVVDSDDLPLNVSRETLQQ

HKLIKVIKKKLVRKVLDMLKKISEEDYEKFWKEYSTNIKLGTIEDPANRTR

LAKLLQFHSSNSDKMTSLADYVKRMKPKQERIFYIAGSSKDEVQKSPFVE

RLLRKGYEVLYLVEAVDEYAISAIPEFEGKKFQNVAKEGFSLTESEGGKEQ

LEQLKTTFEPLTKWLSDEALKDHVAKATVSERLSDSPCALVASMFGWTG

NMERLAISNAHQKSDDPQRSYYLNQKKTLEINPRHPLMRELLKRVNDDP

SDPTAKDMALMLFRTATLRSGYMLRETADFAQSIEAMMRKTLGVPLDEG

VEEEEDLPEDGIPEEESEEIKDDDGDHDEL

>TcHsp70|TC002822-PA

MATGLVSLPIGIDLGTTYSCVGVFSNGTVNIIANDQGNRTTPSFVAFTDVE

RLVGDPAKGQVALNPENTVYDAKRLIGRKFNDPAVQHDMKLWPFQVI

NDNGKPKIRVRYKGETKTFFPEEISSMVLGKMKETAEAYLGTHVTGAVIT

VPAYFNDSQRQATKDAGTIAGLNVLRIINEPTAAAIAYGLDKKGSTERNI

LIFDFGGGTFDVSILNLADGIFEVKSTAGDTHLGGEDIDCRMVDFFTEEFK

RKHKIDLKNNKRALRRLQTACERAKRTLSSATQASVEIDSLANGVDFYT

NISRAKFEELNGDIFRRTLEPVEKALRDAKIDKSKIDDIVLVGGSTRIPKIQS

LLQSLFQGKELNKSINPDEAVAYGAAVQAAILSGDNSEAVRDILLLDVTP

LSLGIETAGGVMAVLIQRNTTIPVKHSQIFSTFADNQPGVSIQVYEGERSM

TRDNNLLGKFELMGIPPAPRGVPQIEVSFDIDANGILNVSAVENATGKTN

QITIRNDKGRLTKEQIEKMVTEAEKFKEQDQQMKATVASKNDLEAYVYQ

MKNLADDPTVASKLSPADKTLLSNTCKEAVEWMGKNQSATEEQNVRK

KEEIENKLKPIIMKLYAGPGQSSQRQFPSHDNNNRGNGPIIDEVD

>TcHsp70|TC007793-PA

MRLIFAILSTLLALATVCESLAVMSVDLGSEWMKIGIVSPGVPMEIALNKE

SKRKSPAVISFRDNVRSFGEEAQTIGTRFPKNAYMYLLDLLGKSINHPLVK

LYKERFPYYEIVEDPERNTILFKHDDNVFYSPEELIAQLLGKAKEFAEQGA

RQPIKECVLTVPGYFNQIERKSLLQAANLAGLKVLQLINDYTAVALNYGI

FRSKDFNETAQYVMFYDMGATSTTATLVSYQTVKTKDKGFVETHPQLSVI

GVGFDRTLGGLEIQLRLRDHLARKFNEMKKTKNDVFANARSMAKLFKE

AGRVKNILSANAEHYAQIEGLLDEEDFKVLVTRDELEQLAGDLFERVGRP

VELALQSAHLTKDIIGQVVLVGAGTRVPKVQEKLQGVVGQDLAKNLNT

DEAATMGAVYKAADLSTGFQVKKFLTRDAVLYPIQVVFERETPEGVKQV

KRTLFSLMNPYPQKKIITFNKYNDDFNFEVNYADLDYLPPNEIANVGQV

NLTEVSLVGVAEALKKNSGENVETKGIKAHFSMDESGILNLVNVELVVE

KTVSETDEEGTFSKLGNTISKLFGGEEKTETGEEPVKNDTEPQKNTTVPKP

EIKPKVITVKEPITTKQKPLTITPLSKKQFDDSLEKLSKLDKVEKELNRRAT

ALNNLESFVIEVQNKLDEDDFVASASQDEVETIRKSCSEVSDWLYEDGSD

ADADTYEKKLDGLKTLTSDLFKRVWEHNERPEALNALNTMLNQSSQFLT

VAKNLTKSTNPERDVFTDGEVEALEKIIKETEEWKSKMIEEQNKLKKYES

VKLTVKSITEKMGAIDREVKYLVNKHRLWRPKKVEKKEESKQEETVEAP

GNTTTESAPEDIKPTESEKDTHTEL

>TcHsp70|TC007965-PA

MSETVIGIDLGTTNSCVCVHLNNKLKILENKEGGRTTPSYVFFTEHSFIVG

QYAKRMADAKPEYGIYEIKRLVGRKYDDPYVKKNLNYLPFKVTNISNEP

VVVVQTKNQVLKKSPQELCAYILGKIKSDVEAKLGHPVDKAVITVPAYF

NIAQREVTLAAAQTAGFSVLKLLNEPTAAALSYYYENKSNVDGYSLVYD

LGGGTFDVAILQRSGSDITIVGVDGETHLGGHDFDNLLVEHVCQVLINQ

HNYNPKNDRRNMRRLNNECEEVKKILSEAEETNIILNAFVPNQNTVDIPI

TRAQFEAKAEQLFQKTIEIVTRCLEKVSLEKSDIKEVILSGGSTRIPKIQSLIS

AYFGGKILNKFINPDECVAEGAAIQAALLSKDPAQAISQIKITDVTPLSLGI

ADFVDVMTFMIKRNTPIPVTRTTNRVTVYNNQANMSFHIYEGERLDAKK

NYFLGRLEITDLTPAPPGQCSVTVVMTVDQNGILTVKATETVSNRTKDLK

IVYTRGSRSDSDIKSVVVEAEENKKEDELFKQFAEVKGYVVKYCIRAMYN

FENKGLVATHKDAYDKCNDVLNRLKDLDVDDEKEVKKLKVEVLALCQ

PLQKKYAFKHMP

>TcHsp70|TC007971-PA

MAAMSVIGIDLGNESCYVAVAKAGGIETIANDYSLRATPSFIAFSDKNRIL

GVAAKNQQITNMKNTVYGLKRLIGRKYRDPHVQRELQMLPFNVIEVAQ

GNIGIKVNYLNEEHIFSPEQCLAMLLTKLKDISSTALQTPINDCVISVPSYFT

NNERKALMDSAAIAGLNVLRLFNETTATALSYGIYKQDLPGPDDKSRNV

VFVDCGHSSLQVFACAFNKDKLRMIATASDPYLGGRDFDLALADHFCKE

FQTKYHIDAKSNARAFSRLLGEVEKVKKQMSANSTSLPLNIECFMDDKD

VHSSIKRTDMEQLCAPLFQRVETTLKQCLDASGLKLDEIYSVEIVGGSSRIP

AIKQLIEKVFKKVPSTTLNQDEAVSRGCALQCAMLSPAVRVREFGVKDV

QNYAVSVSWDASTDGEAAGEVEAFPVNHQVPYSKMLTFYRQEPFSIKAM

YSGNVPYPDKNIGTWIVKDIRPNAEGKPQKVKVKVRINLHGIMTVSSASL

FEAKESSEAENEETQKVQQQEEQEKPTNQNSTDQQNDVDAPMADGVN

NAAPEDGDKDKKKKQVLKSIELPIESLTAGFSQVEINQYTEQEFKMIAAD

RQEKERADARNAFEEYVYELRGKISSDDELGAFILENDRSALMQQLDDM

ENWLYEDGAECNRQVYQDKLAELKSKGEPIQTRKVEFELRPHVIEDFAKS

LQLTMKALEAIKGNDPKFAHLTEDEVKKVDNAFKNSFQWLEQTRAKLV

NAPKHVAPPVTVSQIRQEKNDFECTVSPILNKPPPKAPSPPKEEKSPTDEK

AQNAQTEQNQQNQQNQNQQENMDWSSAN

>TcHsp70|TC009445-PA

MQAKTDLVIGIDLGTTNSSAAYYFKEKVRVVENKEGDRITPSCVYFRDQN

TVIVGKYARKMAEQSNQSEVFGIKRFIGKQFDDPDLRNDLRHVPFTIESIE

NKPIVTINHKSGVCKKTPEEVSALVLQKVKTDVESKLGERVNKAVITVPA

YFNVSQREATLEAAQKAGFTVLKLLNEPTAAAFCYYVDQNWGEESYSLV

YDLGGGTFDVAILKNCRQNIDIVGVDGDTHLGGHDFDNLIIDYVCDILLK

EYDYNPKDDRRNMRRLRSICEEAKQTLSDLEETIIILPAFTKKHDIININITR

EQFESMAQMLFQRTIDIVDKCLTTCNIAKTEIKEVILSGGSTRIPEIQNLLSS

YFGGKELCKFTHPGECVAEGAAIQAAILSTNPDQKINTIQIKDVISLSLGID
VHFNLMFFIIKRNRSIPIKKTKSLITIFNQQSAMSINIYEGERTDVRKNRHLG
TLKITNLTPAPPGQCEVHVIMSVDQNGILTFRAKEKFRNNEKDLKLLYTR
GGRSDSEVKSILQKVEDQAEEDERFEKFAMKKTVLFNYCETVIYNLESKN
LSSSYKEVYDLCKDTQNKLESLELGSEDQVNPLVEAAKSKCDSLVRQYNF
DYMFDL

>TcHsp70|TC009126-PA

MVKSPAVGIDLGTTYSCVGVWQHGKVEIIANDQGNRTTPSYVAFTDTER
LLGDAAKNQVAMNPSNTIFDAKRLIGRKFDDPKIQQDIKHWPFKVINDC
GKPKIQVEHKGEVKKFAPEEISSMVLTKMKETAEAYLGTSVRDAVITVPA
YFNDSQRQATKDAGVIAGLNVMRIINEPTAAALAYGLDKNLKGERNVLI
FDLGGGTFDVSILTIDEGSLFEVRATAGDTHLGGEDFDNRLVNHLADEFK
RKYKKDLRSNPRALRRLRTAAERAKRTLSSSTEASIEIDALFDGIDFYTKVS
RARFEELNADLFRGTLQPVEKALTDAKMDKGMIHDIVLVGGSTRIPKIQQ
LLQNYFNGKSLNLSINPDEAVAYGAAVQAAVLSGETDSKIQDVLLVDVT
PLSLGIETAGGVMTKIIERNARIPCKQTQTFTTYADNQPAVTIQVFEGERA
MTKDNNLLGTFDLTGIPPAPRGVPKIEVTFDLDANGILNVSAKDTSSGNS
RNITIKNDKGRLSQKDIDRMVSEAEQYKEEDEKQRQRIAARNHLEGYIFQ
LKQAVSDCGDKLSSADKETITRECDSCLQWLDANTLAEKEEYEDRQKQL
TQICSPIMAKLYQQGAPQGGQMPGSCGQQAGGFGGQRQGGPTIEEVD

>TcHsp70|TC009706-PA

MVKSPAVGIDLGTTYSCVGVWQHGKVEIIANDQGNRTTPSYVAFTDTER

LLGDAAKNQVAMNPSNTIFDAKRLIGRKFDDPKIQQDIKHWPFKVINDC

GKPKIQVEHKGEVKKFAPEEISSMVLTKMKETAEAYLGTSVRDAVITVPA

YFNDSQRQATKDAGVIAGLNVMRIINEPTAAALAYGLDKNLKGERNVLI

FDLGGGTFDVSILTIDEGSLFEVRATAGDTHLGGEDFDNRLVNHLADEFK

RKYKKDLRSNPRALRRLRTAAERAKRTLSSSTEASIEIDALFDGIDFYTKVS

RARFEELNADLFRGTLQPVEKALTDAKMDKGMIHDIVLVGGSTRIPKIQQ

LLQNYFNGKSLNLSINPDEAVAYGAAVQAAVLSGETDSKIQDVLLVDVT

PLSLGIETAGGVMTKIIERNARIPCKQTQTFTTYADNQPAVTIQVFEGERA

MTKDNNLLGTFDLTGIPPAPRGVPKIEVTFDLDANGILNVSAKDTSSGNS

RNITIKNDKGRLSQKDIDRMVSEAEQYKEEDEKQRQRIAARNHLEGYIFQ

LKQAVSDCGDKLSSADKETITRECDSCLQWLDANTLAEKEEYEDRQKQL

TQICSPIMAKLYQQGAPQGGQMPGSCGQQAGGFGGQRQGGPTIEEVD

>TcHsp70|TC010172-PA

MVKSPAVGIDLGTTYSCVGVWQHGKVEIIANDQGNRTTPSYVAFTDTER

LLGDAAKNQVAMNPSNTIFDAKRLIGRKFDDPKIQQDIKHWPFKVINDC

GKPKIQVEHKGEIKKFAPEEISSMVLTKMKETAEAYLGTSVRDAVITVPAY

FNDSQRQATKDAGVIAGLNVMRIINEPTAAALAYGLDKNLKGERNVLIF

DLGGGTFDVSILTIDEGSLFEVKATAGDTHLGGEDFDNRLVNHLADEFKR

KYKKDLRSNPRALRRLRTAAERAKRTLSSSTEASIEIDALFDGIDFYTKISR

ARFEEMNADLFRGTLQPVEKALTDAKMDKGMIHDIVLVGGSTRIPKIQQ

LLQNYFNGKPLNLSINPDEAVAYGAAVQAAVLSGETDSKIQDVLLVDVT

PLSLGIETAGGVMTKIIERNARIPCKQTQTFTTYADNQPAVTIQVFEGERA

MTKDNNLLGTFDLTGIPPAPRGVPKIEVTFDLDANGILNVSAKDTSSGNS

RNITIKNDKGRLSQKDIDRMVAEAEQYKEEDEKQRQRIAARNQLEGYIFQ

LKQTISEQGSKLSEADKETVTRECDSCLQWLDANTLAEKEEYEDKQKQLS

SICAPIMAKLYQQGGQGGMPGAGGSCGQQAGGFGGQRQGGPTIEEVD

>TcHsp70|TC004425-PA

MYSYLHVALATLTTERKKSKEMKLHLALGALCLVLSVWAKDDKKEKED

VGTVIGIDLGTTYSCVGVYKNGRVEIIANDQGNRITPSYVAFTADGERLIG

DAAKNQLTTNPENTIFDAKRLIGRDWSEQSVQNDIKFFPFKVIEKNSKPHI

AVETSQGNKVFAPEEISAMVLGKMKETAEAYLGKKVTHAVVTVPAYFN

DAQRQATKDAGTIAGLVVMRIINEPTAAAIAYGLDKKDGEKNVLVFDLG

GGTFDVSLLTIDNGVFEVVATNGDTHLGGEDFDQRVMDHFIKLYKKKKG

KDIRKDNRAVQKLRREVEKAKRALSSAHQVRIEIESFFEGDDFSETLTRAK

FEELNMDLFRSTMKPVQKVLEDADMNKKDVDEIVLVGGSTRIPKVQQLV

KEFFGGKEPSRGINPDEAVAYGAAVQAGVLSGEQDTDAIVLLDVNPLTM

GIETVGGVMTKLIPRNTVIPTKKSQVFSTASDNQHTVTIQVYEGERPMTK

DNHLLGKFDLTGIPPAPRGVPQIEVTFEIDANGILQVSAEDKGTGNREKIV

ITNDQNRLTPEDIDRTS

>TcHsp70|TC000487-PA

MLAAARVLSRKAVECSKFSYDVVGKRNFSYLTNNTSSYTLHPKYDIQTRF

KSEGVKGAVIGIDLGTTNSCVAVMEGKQAKVIENSEGSRTTPSVVAFTKD

GERLVGMPAKRQAVTNSANTFYATKRLIGRRFDDSEVKKDMNNVSYKIV

KASNGDAWVQGSDGKMYSPSQIGAFILTKMKETAEAYLNTKVKNAVITV

PAYFNDSQRQATKDAGQIAGLNVLRVINEPTAAALAYGMDKTEDKIIAV

YDLGGGTFDISILEIQKGVFEVKSTNGDTFLGGEDFDNVLVNHLVSEFKKE

QGIDVTKDPMAMQRLKEAAEKAKIELSSSMQTDINLPYLTMDASGPKH

MNLKLSRSKFESLVGELVKRTVQPCQKALKDAEVAKNEIGEVLLVGGMT

RMPKVQSTVQDIFGKQPSRAVNPDEAVAVGAAVQGGVLAGDVTDVLLL

DVTPLSLGIETLGGVFTRLISRNTTIPTKKSQVFSTAADGQTQVEIKVHQGE

REMAADNKLLGQFSLVGIPPAPRGVPQIEVTFDIDANGIVHVSARDKGTG

KEQQIVIQSSGGLSKEEIENMVRRAEEYAKEDKIKKERVEAINQAEGIVHD

TETKMEEYKDQLPKEECDKLKEEIAKVREMLAKKDEADPEEIRKVTGTLQ

QSSLKLFEMAYKKMAADREGSGGSSSGSSEQQSEEPKEKKEDKN

>TcHsp70|TC000188-PA

MGKVPGIGIDLGTTYSCVGVWQHGKVEIIANDQGNRTTPSYVAFTDTERL

IGDAAKSQVAMNPKNTVFDAKRLIGRKFDDTKIQEDMKHWPFTVINDG

GKPKIQVEYKGEIKKFAPEEISSMVLTKMKEIADTYLGAKVNDAVITVPAY

FNDSQRQATKDAGAIAGLNVLRIINEPTAAALAYGLDKNLKGEKNVLIF

DLGGGTFDVSILSIDEGSLFEVKSTAGDTHLGGEDFDNRLVNHFIQEFKRK

HHKDLSSNTRAVRRLRTACERAKRTLSSSAEASIEIDALHEGIDFYSKVSR

ARFEEMCMDYFRSTLQPVERALADAKLDKGAIHDIVLVGGSTRIPKIQKM

LQDFFCGKPLNLSINPDEAVAYGAAVQAAILTGDTSSQIQDVLLVDVAPL

SLGIETAGGVMTKIVERNSRIPCKQQQTFTTYSDNQNAVTIQVFEGERAM

TKDNNLLGTFNLTGIPPAPRGVPKIEVTFDLDANGILNVSAKDTSTGKSER

ITITNDKGRLSKADIDKMLAEAEKYKAEDDKQKERIAARNQLEGYIFSVK

QAGEDAPADKLTEDDKKIIREKCSAALSWLDSNQLAEKEEFEDKLKELQ

KDCSPIMMKLHQGAQGGAAPGAKGPTVEEVD

>TcHsp70|TC005884-PA

MSELVVGIDLGTTNSCIAVERNGKIEVIPNREGNRTTLSYVYYGEDSILVG

KTAKYMASANPSNGIYEIKRLIGCLHDDPDIESERKSLNYELVRGTNGEILI

QVEQNSEKFHTLPEEVCARILHRLKIDAEMYLGQKVSKAVVTVPAYFNN

NQRAATRDAARIAGFEVLKLVNEPSAAALAYVRENRIKNGRVILIYDLGG

GTFDVSIVRTENGTIKVLSVDGDTHLGGQDFLNRLVDHVVDYVQTKHGI

KVRENKRLMMNILNSCEKTKKILTSANRTVIPLEFSGHFDQLEVTREQFEE

LNRDLFAKTVKILDNCIRNRRMSKEEIDEVLLVGGSSRIPRIETLLKAYFDK

PIQRNINADEAIAIGAALEAHHFAQTHHNSLLIDVLPLSIGTVFDEETIFFN

FARNTPLPANSKHVHVFKNRKQKNCILSVYEGGHLDCNKNVLLGAHEI

KWTATTKKNRNVEITMQINNYGIIWVTARGESIKTFSIALNKGRLEDDEIR

KLSRGLQVS

>TcsHsp21.8|TC010105-PA

MSVVPLLFRDWWDDDDFHFSRPSRLLDQQFGLGLRRDDLLNTFSSMPRS

SLFRNYVRPWRSTAIQRQDSGSTIQQDKDKFQVILDVQQFAPNEITVKTSG

NSIIVEGKHEEKQDEHGFISRHFVRRYLLPQDHDINDVVSSLSSDGILTVSA

PKKSLQQPAGERVVPITQTGPAKATVTPVAESQPKVEQPN

>TcsHsp23.6|TC001152-PA

MSEGIRRDIPIKLGDFSVIDTEFSSIRERFDAEMRKMEEEMSKFRSELMNRE

SNNFFRSTTRSYEYETVSGGNKSKSSSTTTQSSHNSGLDVAQRPSEVRTWY

DDLNSPLIQQDGNDKCLKLRFDVSQYAPEEIVVKTVDNKLLVHAKHEEK

TESKSVYREYNREFLLPKGTNPEQIKSSLSKDGVLTVEAPLPAITAGETLIPI

QH

>TcsHsp22.2|TC003541-PA

MALLSFVTDPLDYFRPSLLLDQQFGMGLDDDDFLQPCLPRKVRRMMLTS

PYARPWRSQASKKDKGSTLSVDKDKFQVSLDVQQFTPEEITVKASDDTITI

EGKHEEKEDEHGFISRHFIRKYKLPEGHDISQVTSKLSTDGVLTITAPKSEE

KIKERNIPISFTGQPSQIEATPTIEVGADDKKPEEKKEARKRK

>TcsHsp18.3|TC005338-PA

MALWLYTDPFDFYRPAHRFLERWFDPEDLFPRDFRLLQDHGSSDINFDK

DKFQANIDVQQFRPEEITVKVSDDTVTVEGKHEEKRDEHGYISRHFVRKY

VLPKGHDVNRVESKLSSDGVLTITAPKVGDGKEQEKSIPVVQTGQPTPAV

QQKQEEKK

>TcsHsp20.8|TC006793-PA

MSLLLFSDPFEYSRPSRILDQHFGLGLDPEDLLSPLIPREMRHLMRCPAGY

LRPWRSAASQRDTGSTVTFDKDKFQANLDVQQFKPEEISVKVNDNTITIE

GKHEEKEDEHGFISRHFVRRYVLPKDCDVSKVESRLSTDGVLSITAPKICA

SKETERSIPVVQTGQPSKAVENKEEKKKEK

Supplementary Tables

Table 1. Gene-specific primers for q-RT-PCR used in this study.

Gene name	Forward primer (5'-3')	Reverse primer (5'-3')
BtaHsp70-1	GTCTATGAAGGTGAGCGAGCAATGA	CGTTGAGGATACCGTTGGCATCA
BtaHsp70-2	GGAGACCAGAGTGCGGAAAT	AGCGTTCCTCTCGACGATCT
BtaHsp70-3	TGAAGGTGAGCGTGCAGTAG	CGTGACATCAACTGAAGGTACAA
BtaHsc70-1	ACAGCAAGCCTCACATCGAA	GGTTGAAACTACAGGTTTGCCA
BtaHsc70-2	CCCTCTGTCGTAGCATTCTCTA	TTCTTCACTTCAGCATCGTCAA
BtaHsp70-4	TGTGGTCACTGTGCCCGCTTA	CGTTCCGCCTCCGAGATCGTAA
BtaHsp70-5	AGTCGGTGGTCAAACACGCATG	TCGCCACCTAATACGCCTCCTT
BtaHsp70-6	ACTGAACGGAAAGATCAATGCAG	GGTTTCCGACTAAGGCCATCA
BtaHsp105/110	GCGAGTGGTTGGATGAAGAAGG	CAATGCCTCTGGTGTGGTCTCA
BtaHsp70-7	GGATCTTTAAGGCGGAAGATGG	TGAACGCAAGTTGGATGGCT
BtaHsp67B2	TCCGTCCCAATGAGTGCTTGT	GCTTGAGTGACTTCGCCTAAGG

BtaHsp60	ACAGGCAACAAGAGCAGCAGTA	TGGCACGAAGAGCAACACGAAT
BtaHsp90A1	CGCTCTCCTCGCCAGAATTGT	CCAACACCGAACTGACCGATGA
BtaHsp90A2	CCTCTTGCCACGCTACCTGAAC	CTTGGCGAGCCTCACTCTGTTG
BtaHsp90A3	ATTGGAATGACCAAGGCTGACC	AGCGACGAGGAAGGCTGAG
BtaHsp90A4	AGCTCATGGTGCACTTTAGG	GACATCAACTGGGCGATTTCC
BtaHsp100	AGCCGACGGTAGAAGACACCAT	TGCCGCCGCTTCATCAATCAA
BtaHsp23.8	TGTGTTGCAGCTCGACCAA	TGACGTCGAACCTCAGCTTG
BtaHsp40-1	GGTCGTTCTTCGGCGGACATT	TGGTGGAATTGCTGAGCGTGAT
BtaHsp40-2	AAGCGAAGTGAACCAGGAACCA	GCGACCGACTACCATCCTAACG
BtaHsp40-3	CGCCCTGAAATACCACCCTGAC	TCCTCCTCCACCTCCGACATTG
BtaHsp40-4	CGAAGGAATGCCTCAGTACAAGAA	TTATCCGCTGCTATCGAATCTGG
dsgfp	TAATACGACTCACTATAGGGAGAC	TAATACGACTCACTATAGGGAGAC
	CACTGACCCTGAAGTTCATCT	CACGTCTTGTAGTTGCCGTCGT

	GC	C
dsBtaHsp90A3	TAATACGACTCACTATAGGGTGGACAACTGCGAAGACCTCATC	TAATACGACTCACTATAGGGAGACCAGGTTCTTACCGTCGTAGT
